# Reduced methylation correlates with diabetic nephropathy risk in type 1 diabetes 

**DOI:** 10.1172/JCI160959

**Published:** 2023-02-15

**Authors:** Ishant Khurana, Harikrishnan Kaipananickal, Scott Maxwell, Sørine Birkelund, Anna Syreeni, Carol Forsblom, Jun Okabe, Mark Ziemann, Antony Kaspi, Haloom Rafehi, Anne Jørgensen, Keith Al-Hasani, Merlin C. Thomas, Guozhi Jiang, Andrea O.Y. Luk, Heung Man Lee, Yu Huang, Yotsapon Thewjitcharoen, Soontaree Nakasatien, Thep Himathongkam, Christopher Fogarty, Rachel Njeim, Assaad Eid, Tine Willum Hansen, Nete Tofte, Evy C. Ottesen, Ronald C.W. Ma, Juliana C.N. Chan, Mark E. Cooper, Peter Rossing, Per-Henrik Groop, Assam El-Osta

**Affiliations:** 1 Epigenetics in Human Health and Disease Laboratory and; 2 Department of Diabetes, Central Clinical School, Monash University, Melbourne, Victoria, Australia.; 3 Department of Clinical Pathology, The University of Melbourne, Parkville, Victoria, Australia.; 4 University College Copenhagen, Faculty of Health, Department of Technology, Biomedical Laboratory Science, Copenhagen, Denmark.; 5 Folkhälsan Institute of Genetics, Folkhälsan Research Center, Helsinki, Finland.; 6 Department of Nephrology, University of Helsinki and Helsinki University Hospital, Helsinki, Finland.; 7 Research Program for Clinical and Molecular Metabolism, Faculty of Medicine, University of Helsinki, Helsinki, Finland.; 8 Steno Diabetes Center Copenhagen, Herlev, Denmark.; 9 Department of Medicine and Therapeutics,; 10 Hong Kong Institute of Diabetes and Obesity,; 11 Li Ka Shing Institute of Health Sciences, The Chinese University of Hong Kong, Hong Kong, China.; 12 Department of Biomedical Sciences, City University of Hong Kong, Hong Kong, China.; 13 Diabetes and Thyroid Center, Theptarin Hospital, Bangkok, Thailand.; 14 Department of Anatomy, Cell Biology and Physiology, Faculty of Medicine, American University of Beirut, Beirut, Lebanon.; 15 Department of Clinical Medicine, University of Copenhagen, Copenhagen, Denmark.

**Keywords:** Metabolism, Nephrology, Diabetes, Epigenetics

## Abstract

Diabetic nephropathy (DN) is a polygenic disorder with few risk variants showing robust replication in large-scale genome-wide association studies. To understand the role of DNA methylation, it is important to have the prevailing genomic view to distinguish key sequence elements that influence gene expression. This is particularly challenging for DN because genome-wide methylation patterns are poorly defined. While methylation is known to alter gene expression, the importance of this causal relationship is obscured by array-based technologies since coverage outside promoter regions is low. To overcome these challenges, we performed methylation sequencing using leukocytes derived from participants of the Finnish Diabetic Nephropathy (FinnDiane) type 1 diabetes (T1D) study (*n* = 39) that was subsequently replicated in a larger validation cohort (*n* = 296). Gene body–related regions made up more than 60% of the methylation differences and emphasized the importance of methylation sequencing. We observed differentially methylated genes associated with DN in 3 independent T1D registries originating from Denmark (*n* = 445), Hong Kong (*n* = 107), and Thailand (*n* = 130). Reduced DNA methylation at CTCF and Pol2B sites was tightly connected with DN pathways that include insulin signaling, lipid metabolism, and fibrosis. To define the pathophysiological significance of these population findings, methylation indices were assessed in human renal cells such as podocytes and proximal convoluted tubule cells. The expression of core genes was associated with reduced methylation, elevated CTCF and Pol2B binding, and the activation of insulin-signaling phosphoproteins in hyperglycemic cells. These experimental observations also closely parallel methylation-mediated regulation in human macrophages and vascular endothelial cells.

## Introduction

The rise in prevalence of diabetes and the increased burden of morbidity and mortality are attributable to the development of complications and kidney disease remains the leading cause of end-stage renal disease (ESRD) worldwide (1). Though tight control of blood glucose can decrease the risk of diabetic nephropathy (DN) (2), many individuals despite adequate glycemic control develop DN, while others remain complication-free in spite of poor glycemic control (3). Furthermore, few family studies suggest a role for inherited factors in disease progression to DN (4), with more than 3 times greater incidence in children with diabetes of individuals with nephropathy than without renal disease (5, 6). The heritability of DN remains unexplained (7).

Despite the advances in high-throughput mapping using genome-wide association studies (GWAS) by international consortia, including the Finnish Diabetic Nephropathy (FinnDiane) study, only a few genes have been identified and account for less than 5% of susceptibility to DN (8). The SUMMIT Consortium, which consisted of the FinnDiane (9), the EURODIAB Family Study (10), the Scania Diabetes Registry (11), and the UK Nephropathy Family Study and Oxford Regional Prospective Study (12, 13), found no genetic variants at genome-wide significance (14). A larger and more recent meta-analysis examining 19,406 individuals of European descent with type 1 diabetes (T1D) identified 16 significant risk loci, showing the strongest association with the missense mutation in the collagen type IV alpha 3 chain (*COL4A3*) gene (15).

While there is increasing interest in epigenetics as it pertains to diabetes and its complications, methylation sequencing (methyl-seq) remains largely uncharted and is partly attributed to the popular use of arrays (16). Protocols that assess DNA methylation have their own technical merits, with no standard method nor consensus for methylation detection. The considerations of genomic coverage and resolution are arguably critical yet often compromised in population studies because of the costs associated with sequencing. Of the approaches for detecting DNA methylation, BeadChip arrays dominate T1D population studies (17–20). Relevant here and important to the field, the DCCT/EPIC study group has shown that peripheral blood DNA methylation and hemoglobin A1c (HbA1c) are associated with the risk of complications (21).

**Figure 1:**
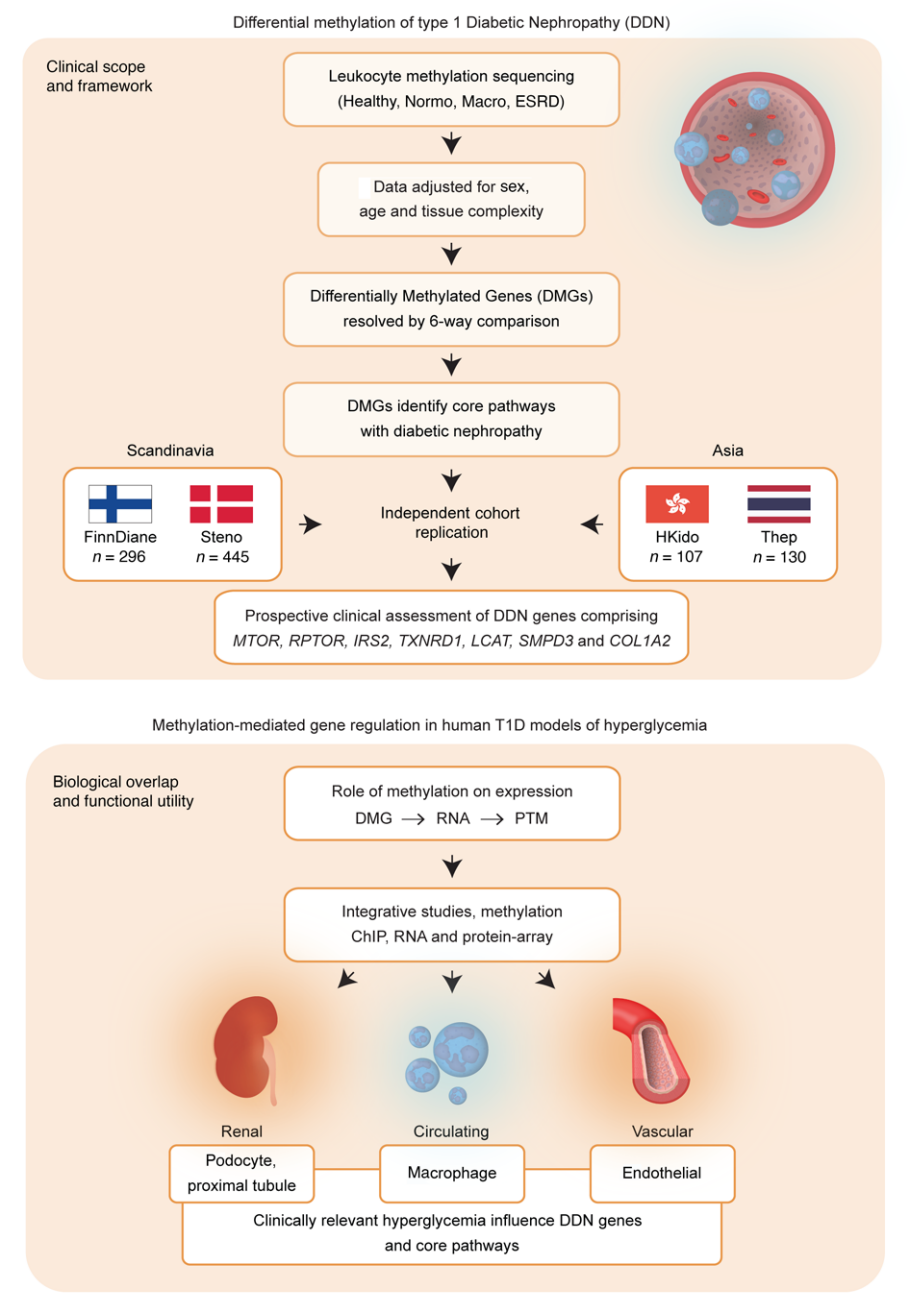
Clinical framework and experimental overlap. The clinical scope and framework for determining differential methylation in type 1 diabetic nephropathy using Scandinavian and Asian cohorts. Genome-wide DNA methylation was analyzed to identify leukocyte-based DMRs in T1D participants from the Finnish Diabetic Nephropathy (FinnDiane) study using methyl-capture coupled with sequencing. DMRs were annotated to differentially methylated genes (DMGs) associated with progression of diabetic nephropathy (DDN) and validated in a larger subset of the FinnDiane study (*n* = 296) and independent cohorts from Denmark (*n* = 445), Hong Kong (107), and Thailand (*n* = 130). Biological overlap and functional utility were assessed for methylation-mediated gene regulation in human cell models of hyperglycemia. We performed functional studies in human podocytes, proximal convoluted tubule cells, macrophages, and vascular endothelial cells and confirmed methylation-mediated regulation of core genes *MTOR*, *RPTOR*, *IRS2*, *COL1A2*, *TXNRD1*, *LCAT*, and *SMPD3*. PTM, posttranslational modification.

Constructing an epigenetic network for DN has its challenges, with no satisfactory array-based method to identify DNA methylation with sufficient coverage to be informative for transcription factor binding sites (TFBSs). With sequencing technologies to characterize DNA methylation, we assessed CTCF and Pol2B recognition sites to reveal network relationships that influence the progression of DN. We observed reduced methylation in 4 T1D cohorts and confirmed a set of core genes associated with disease development. Moreover, these studies show that the mechanisms dependent on methylation involve the assembly of chromatin to regulate gene function using clinically relevant models of hyperglycemia in human renal, circulating, and endothelial cells (Figure 1).

## Results

### DNA methylation profiling in T1D individuals with and without nephropathy.

The discovery group consisted of 25 FinnDiane (12 males, 13 females) participants with T1D characterized into normal albumin excretion rate (Normo), macroalbuminuria (Macro), and ESRD (Supplemental Table 1; supplemental material available online with this article; https://doi.org/10.1172/JCI160959DS1). Individuals with T1D were 27 to 61 years of age (average 49) with a mean duration of diabetes of 34 years and the HbA1c (at the time of sample collection) was approximately 8% and indicative of moderate glycemic control. The nondiabetic group consisted of 14 participants (7 males, 7 females) with HbA1c below 5.5% and the absence of renal disease (no evidence of proteinuria and normal estimated glomerular filtration rate, eGFR). Leukocyte DNA was isolated from all groups and characterized using methyl-seq (22). The mean average number of mapped reads was 27.7 million. Methyl-seq reads covered 35% (~10 million) of the 28 million CG sites in the human genome (Supplemental Table 2), with uniform distribution of CG site coverage between groups. We identified 800,021 variably methylated regions (mean region length 510 bp) that were adjusted for age, sex, cell heterogeneity, and library cluster concentration (Supplemental Figure 1, A–D). Principal component analysis (PCA) showed clustering for control and case groups. Based on methylation indices, the nondiabetic group was closer to T1D individuals without complications (Normo) than T1D with renal complications (Supplemental Figure 1E). Differentially methylated regions (DMRs) were identified using pairwise comparisons of case and control groups (edgeR; significance threshold of *P* < 0.01) (Supplemental Figure 2A). Analysis of T1D individuals with and without complications and the nondiabetic group using 6-way comparison identified 10,078 DMRs (*P* < 0.01 in all groups). We identified 1687 DMRs in the ESRD group with reduced methylation, 3227 DMRs in the Macro group with reduced methylation, and 1792 in the Normo group with reduced methylation, whereas 3362 DMRs showed increased methylation in all cases compared with controls (Supplemental Figure 2B).

### DMRs distinguish DN groups.

**Figure 2:**
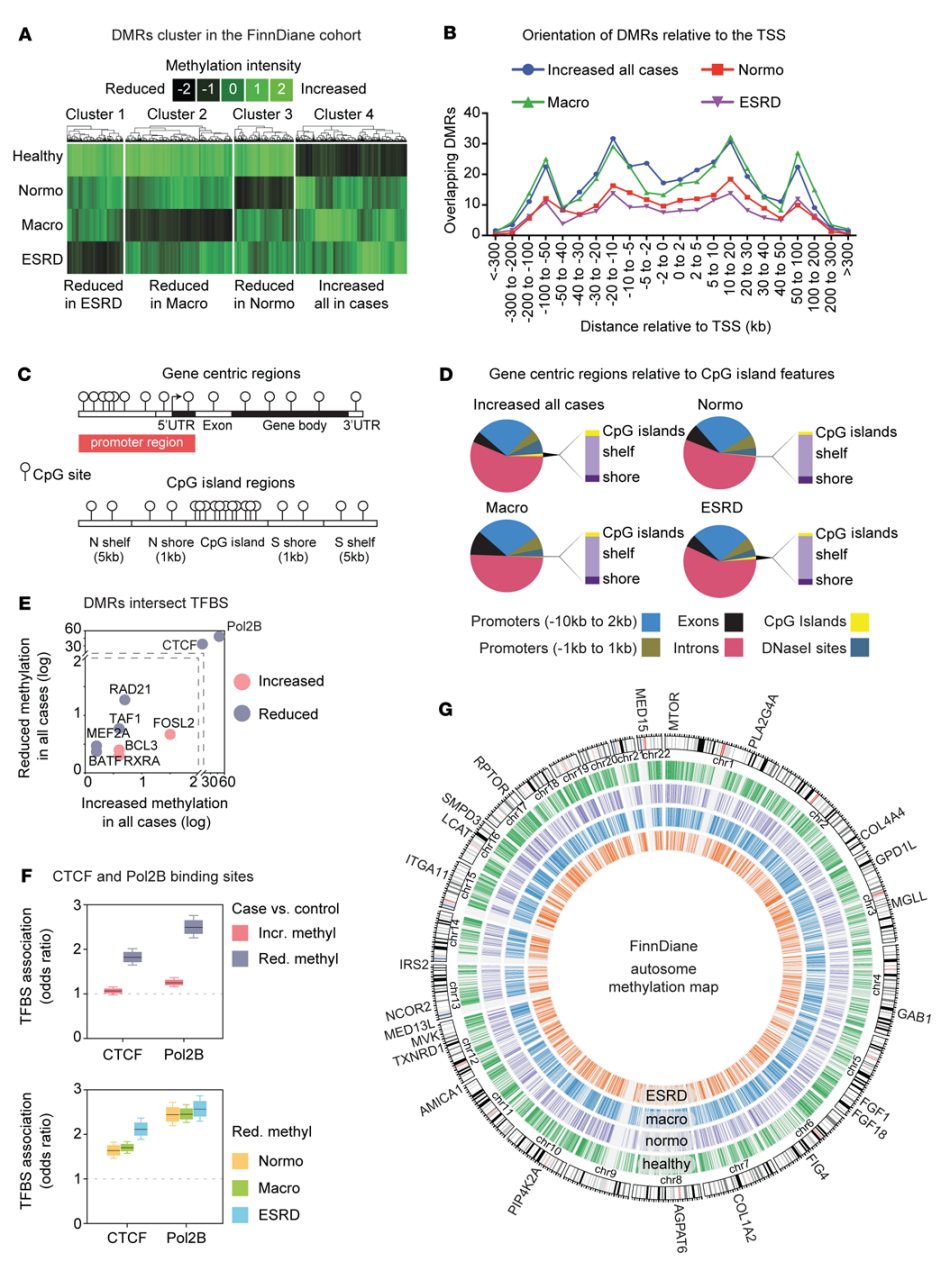
FinnDiane T1D methylome at sites of regulation. (**A**) Hierarchical profile-based clustering of DMRs. DNA methylation differences detected between controls and cases with normoalbuminuria (Normo), macroalbuminuria (Macro), and end-stage renal disease (ESRD). Heatmap shows loss (black) and gain (green) of methylation. We observed clustering of DMRs by the prevalence of DN (*P* value < 0.01). (**B**) Binned orientation and distance of DMRs relative to transcription start site (TSS). (**C**) Graphical representation of genomic features and CG islands, shelves, and shores. (**D**) Distribution of DMR clusters shown at gene promoters, exons, introns, CG islands (CGI), and CG island shores (±1 kb from CGI) and shelves (±1–5 kb from CGI). The majority of CG differences occur at gene intronic regions. (**E**) Scatterplot of DMR overlap with TFBSs (ENCODE data). The *x* axis shows increased methylation at sites compared with reduced methylation at sites in T1D cases (*y* axis). TFBSs that associate with DMRs are shown as increased in methylation (pink) circles and reduced in methylation (purple) in T1D cases when compared with healthy controls. Criteria for TFBS overlap with DMRs was set to 50%. (**F**) CTCF and Pol2B binding sites are overrepresented at DMRs with reduced methylation in T1D cases when compared with healthy controls. Within each box, horizontal black lines denote median values; boxes extend from the 25th to the 75th percentile of each group’s distribution and the whisker denote the 5th and 95th percentiles. CTCF and Pol2B binding sites were overrepresented at sites of reduced methylation in diabetics that developed renal complications. All *P* values < 0.001. (**G**) Atlas of DNA methylation from FinnDiane discovery cohort. Human chromosome ideogram of DMRs clustered by DN (*P* < 0.01). The outermost track is organized by autosomes, showing several methylation-dependent genes. The centromere of each chromosome (chr) is represented by a double red line. The second track represents a genomic view of 3362 regions with increased methylation in all cases (hypomethylated in healthy group) (green, *P* < 0.01). The second track represents 1792 regions with decreased methylation in the Normo group (purple, *P* < 0.01). The fourth track represents the Macro group with 3227 regions with decreased methylation (blue, *P* < 0.01). The fifth track represents the genomic view of 1697 regions with decreased methylation detected in the ESRD group (orange, *P* < 0.01).

Supervised hierarchical clustering shows reduced DNA methylation in individuals with T1D compared with healthy controls (clusters 1 to 4; Figure 2A and Supplemental Table 3). Sequencing identified a bimodal DMR distribution (Figure 2B). Gene body regions accounted for more than 60% of the methylation differences, with less than 10% localized to exons compared with other genomic sites such as promoters, exons, introns, DNase I–hypersensitive sites, CpG islands (CGIs), CpG shores (±1 kb from CGIs), and CpG shelves (± 1–5 kb from CGIs; Figure 2, C and D). CpG islands, shores, and shelves accounted for less than 5% of the total DMRs.

### TFBSs are subject to differential DNA methylation.

The most direct mechanism whereby DNA methylation regulates gene expression is by altering the binding of transcription factors (23). This methylation-dependent mechanism has the advantage of unifying polygenic pathways that are known to be important in T1D (24). To investigate this hypothesis, we integrated FinnDiane DMRs with current ENCODE data sets (25). We identified TFBSs that were weakly associated with methylation and a much smaller group of tightly associated sites that recognize CCCTC-binding factor (CTCF) and DNA polymerase epsilon catalytic subunit B (Pol2B) (Figure 2E). More than 35% of regions with reduced methylation overlap with CTCF and almost 50% with Pol2B sites in T1D cases. Reduced genomic methylation was a feature at CTCF and Pol2B binding sites (odds ratio testing *P* < 0.001; Figure 2F). Because of the close relationship between DNA methylation and gene regulation (26), we examined CTCF binding sites using ENCODE chromatin immunoprecipitation and sequencing (ChIP-seq) data (26, 27) prioritized from studies performed in clinically relevant human tissues, including heart, kidney, retina, immune cells, and the vasculature. To visualize the FinnDiane data set, we generated an epigenomic ideogram (clusters 1 to 4 at *P* < 0.01; Figure 2G) and showed that DNA methylation converges on CTCF and Pol2B binding sites with DN progression.

### Functional networks identify methylated genes with DN progression.

**Figure 3:**
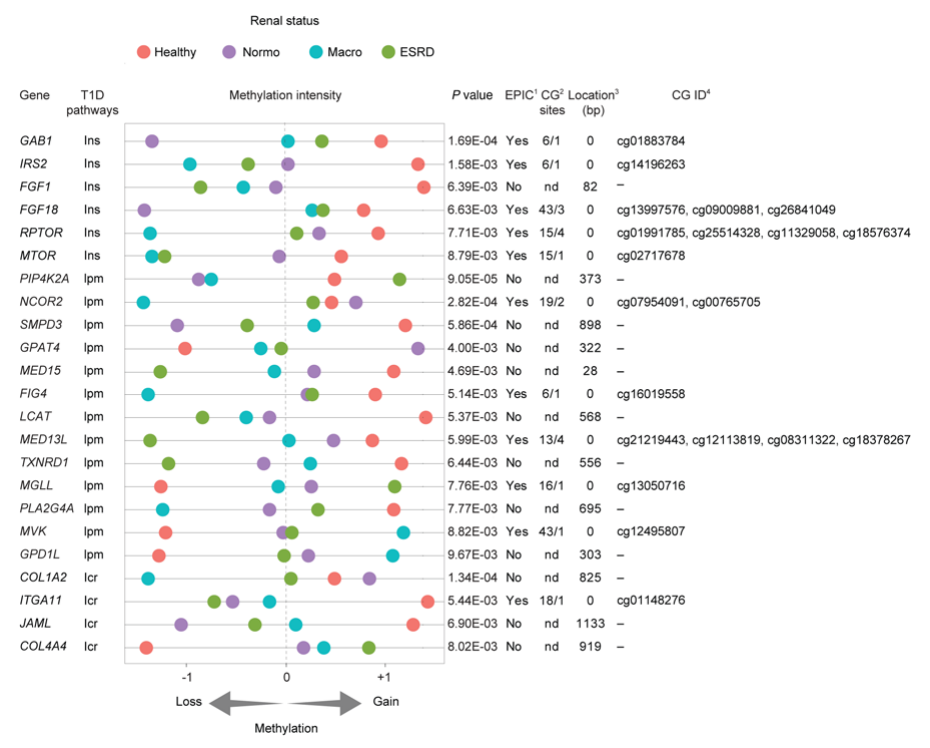
Functional analysis of FinnDiane cohort identified methylated genes associated with the progression of DN. Differentially methylated genes associated with progression of diabetic nephropathy (DDN) with overlapping CTCF sites. Gene - annotated gene name; T1D-associated pathways with insulin signaling (Ins), lipid metabolism (lpm), and integrin-cell interaction (Icr); *P* value; Region length (bp); Methylation Intensity - scaled sequence abundance (–1, unmethylated; +1, methylated), showing relative gene methylation in healthy, Normo, Macro, and ESRD. Table also shows methyl-seq genes identified in the FinnDiane cohort intersected with probes from the Infinium Methylation EPIC profiling BeadChip microarray (EPIC array). Presence on the EPIC array; Number of CG sites assessed by methyl-seq overlapping EPIC array probe; Distance (bp) of the closest EPIC array probe from methyl-seq identified sequences. ^1^DMRs detected by methyl-seq–overlapping Infinium methylation EPIC BeadChip array probes. ^2^Comparison of CG number detected by methyl-seq versus EPIC probe CG location; nd denotes no detected probes on EPIC array. ^3^Distance of nearest EPIC array probe; 0 denotes EPIC CG site that exists within FinnDiane DMR. ^4^EPIC CG ID listed.

Gene set enrichment analysis (GSEA) was used to examine DMRs annotated to genes (DMGs) with reactome pathways, consisting of 674 gene sets. Supplemental Table 3 shows the number of enriched reactome pathways (FDR < 0.05). We show that FinnDiane DMGs define clinically relevant networks (Supplemental Figure 3A) connecting 15 hubs and 125 nodes by DNA methylation. High-confidence mapping revealed 64 nodes associated with increased methylation and 61 nodes connected with reduced methylation. Supplemental Figure 3B shows reactome pathways that are distinguishable for DNA methylation also converge for DN progression. Reduced methylation is a feature of CTCF binding sites that include insulin signaling, lipid metabolism, and integrin-cell interactions or fibrosis (28). These 3 pathways were overrepresented in the T1D cases with overt proteinuria and ESRD. We also identified 23 DMRs with CTCF binding sites (Figure 3). Moreover, 12 of the 23 DMRs associated with DN were undetectable as probe sites by BeadChip array, emphasizing the importance of the methyl-seq approach (Figure 3 and Supplemental Table 4). We described 7 core genes with overlapping CTCF binding sites: *MTOR*, *RPTOR*, and *IRS2* that belong to the insulin signaling pathway; *TXNRD1*, *LCAT*, and *SMPD3* comprising lipid metabolism; and *COL1A2* involved in fibrosis. We defined these as DMGs associated with DN, or DDNs.

### Reduced methylation is a genomic feature of Scandinavian and Asian T1D registries.

**Figure 4:**
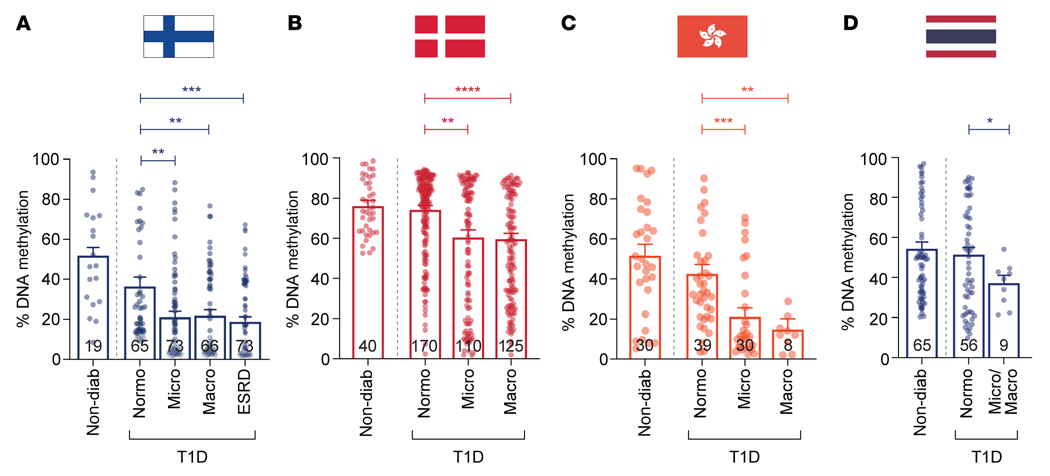
Validation of differentially methylated genes in replication cohorts. (**A**) DNA methylation analysis of core genes was performed in a larger FinnDiane replication cohort (*n* = 296: 19 healthy, 65 Normo, 73 Micro, 66 Macro, and 73 ESRD) using a highly specific methyl-qPCR assay. Data show combined DNA methylation (%) for the 7 core genes: *MTOR*, *RPTOR*, *IRS2* (insulin signaling), *TXNRD1*, *LCAT*, *SMPD3* (lipid metabolism), and *COL1A2* (integrin-cell interaction) using PCA loading analysis. Results show that reduced DNA methylation (%) is associated with DN. (**B**) Replication of FinnDiane-derived DDNs in samples from the Danish PROFIL study – Steno Diabetes Center Copenhagen. Methyl-qPCR methylation analysis includes 40 nondiabetic and T1D individuals with Normo (*n* = 170), Micro (*n* = 110), and Macro (*n* = 125). (**C**) Methylation analysis of the DDNs in 77 age-matched T1D individuals from the Hong Kong T1D registry and 30 healthy controls. Individuals with T1D include 39 Normo, 30 Micro, and 8 Macro. (**D**) Methylation analysis of the DDNs in age-matched nondiabetic and T1D individuals recruited from the Theptarin registry, Thailand. DNA methylation (%) was assessed for genes in 65 controls and 65 cases (56 without renal complications and 9 with renal complications). Significance was calculated using the Mann-Whitney *U* test by comparing T1D with no complications (Normo) to Micro, Macro, and ESRD (**A**) or by comparing T1D with Normo and 9 T1D with Micro/Macro (combined) (**B**–**D**). *P < 0.05; **P < 0.01; ***P < 0.001; ****P < 0.0001. Error bars are SEM.

We examined the DDNs in a larger FinnDiane replication cohort (*n* = 296; Supplemental Table 5) and confirmed that reduced gene methylation was a feature of DN progression using a gene-specific methyl-qPCR assay (Figure 4A). To assess the generalizability of these findings, we assessed the core DDNs using independent T1D registries from Denmark (PROFIL study, *n* = 445; Supplemental Table 6) and Hong Kong (*n* = 107; Supplemental Table 7) and confirmed that reduced methylation was also associated with DN progression (Figure 4, B and C). Next, we examined DNA methylation of core genes in the T1D (*n* = 130; Supplemental Table 8) cohort from the Diabetes and Thyroid Center, Theptarin Hospital, Thailand and reveal reduced methylation in the combined Micro/Macro groups (Figure 4D).

**Figure 5:**
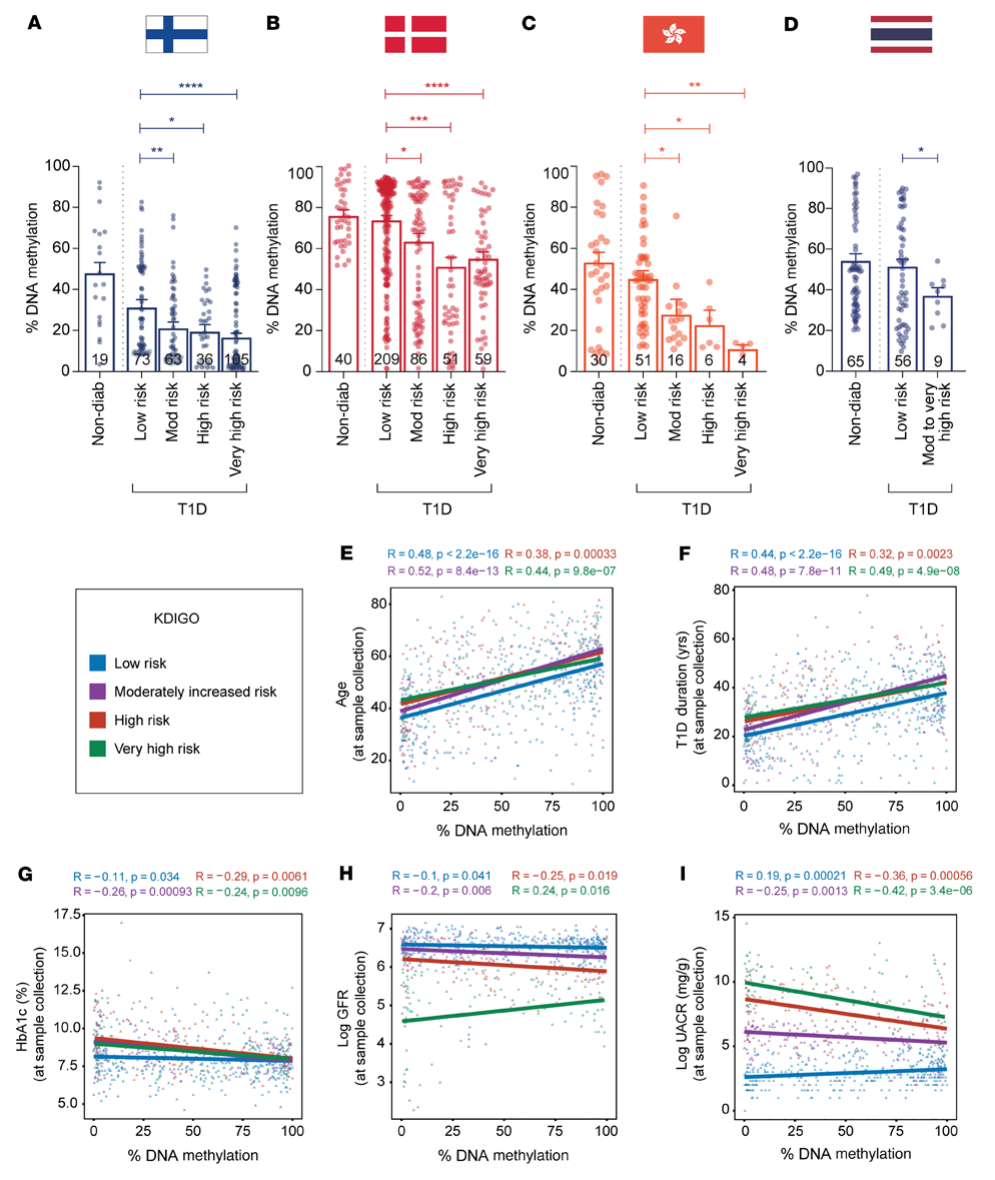
Differentially methylated genes associate with the progression of diabetic nephropathy in T1D cohorts based on KDIGO classification. DNA methylation analysis of genes associated with diabetic nephropathy (DN) identified in the discovery cohort was validated in replication cohorts (**A**) FinnDiane, (**B**) PROFIL, (**C**) HKT1D, and (**D**) Theptarin T1D using methyl-qPCR assay. Samples from the replication cohorts were separated into 5 groups: nondiabetic and individuals with diabetes with low risk, moderate risk, high risk, and very high risk of developing DN as defined by the KDIGO classification. Data show the percentage DNA methylation (combined core gene set) for the different groups presented as bar plots and SEM, with significance calculated by comparing diabetics with low risk to those with moderate risk, high risk, and very high risk using the Mann-Whitney *U* test. *P < 0.05, **P < 0.01, ***P < 0.001, ****P < 0.0001. The DDNs include *MTOR*, *RPTOR*, *IRS2*, *COL1A2*, *TXNRD1*, *LCAT*, and *SMPD3*. Correlation plot between the combined core gene set and clinical covariates such as (**E**) age, (**F**) T1D duration, and (**G**) hemoglobin A1c (HbA1c %) and 2 key markers of chronic kidney disease: (**H**) estimated glomerular filtration rate (eGFR) and (**I**) urine albumin-to-creatinine ratio (UACR). Regression lines are shown for DNA methylation (combined methylation of core genes) versus age, T1D duration, HbA1c, eGFR, and UACR. Pearson’s correlation coefficient (*R*) and *P* value are reported for each group colored by KDIGO classification: T1D individuals with low risk (blue), with moderately increased risk (purple), and T1D individuals with high (red) and very high risk of developing ESRD (green). Covariate regression analysis was conducted for participants from Finland, Denmark, Hong Kong, and Thailand T1D cohorts (*n* = 824 T1D individuals). Nondiabetic controls were excluded.

Differential methylation was also assessed using the KDIGO (Kidney Disease: Improving Global Outcomes) classification, as defined by the combination of eGFR and albuminuria (Supplemental Table 9) (29). Reduced methylation associated with increased DN risk (Figure 5, A–D). This primary finding was also confirmed for the 7 core genes in the Scandinavian and Asian T1D cohorts (Supplemental Figure 4, A–D). Weighted meta-analysis of the DDNs (Supplemental Table 10) shows that reduced methylation was tightly associated in the high- to very-high-risk groups. Combined *P* values of the core methylated genes (Fisher’s combined probability test) ranged from 2.39 × 10^–3^ to 7.75 × 10^–9^ in the high- to very-high-risk groups. The NF-κB target gene, *TRAPPC9*, served as a stable internal control for DNA methylation (Supplemental Figure 5A). Methylation of the *TXNIP* gene (thioredoxin-interacting protein) (21, 30) and *KCNQ1* (potassium voltage-gated channel subfamily Q member 1) (31) was previously shown to associate with the development of diabetes complications. We observe reduced DNA methylation of the *TXNIP* and *KCNQ1* genes in the FinnDiane and Asian registries, adding further support to their relevance as genetic markers of DN (Supplemental Figure 5, B and C).

### Reduced gene methylation associates with HbA1c but not with age and diabetes duration.

While there was no correlation for DNA methylation (renal complications when compared to no complications) using age and diabetes duration (Figure 5, E and F), we did observe an association for reduced DNA methylation with HbA1c levels (Figure 5G) and eGFR (Figure 5H). Furthermore, increased urinary albumin-to-creatinine ratio (UACR) was also associated with reduced methylation of core genes in the renal complication groups (Figure 5I). Reduced methylation of the 7 core genes in the Scandinavian and Asian T1D cohorts correlated with percentage HbA1c, eGFR, and UACR (Supplemental Figure 6, A–C).

### Reduced gene methylation predicts the progression of albuminuria and improves estimation of renal functional decline.

**Figure 6:**
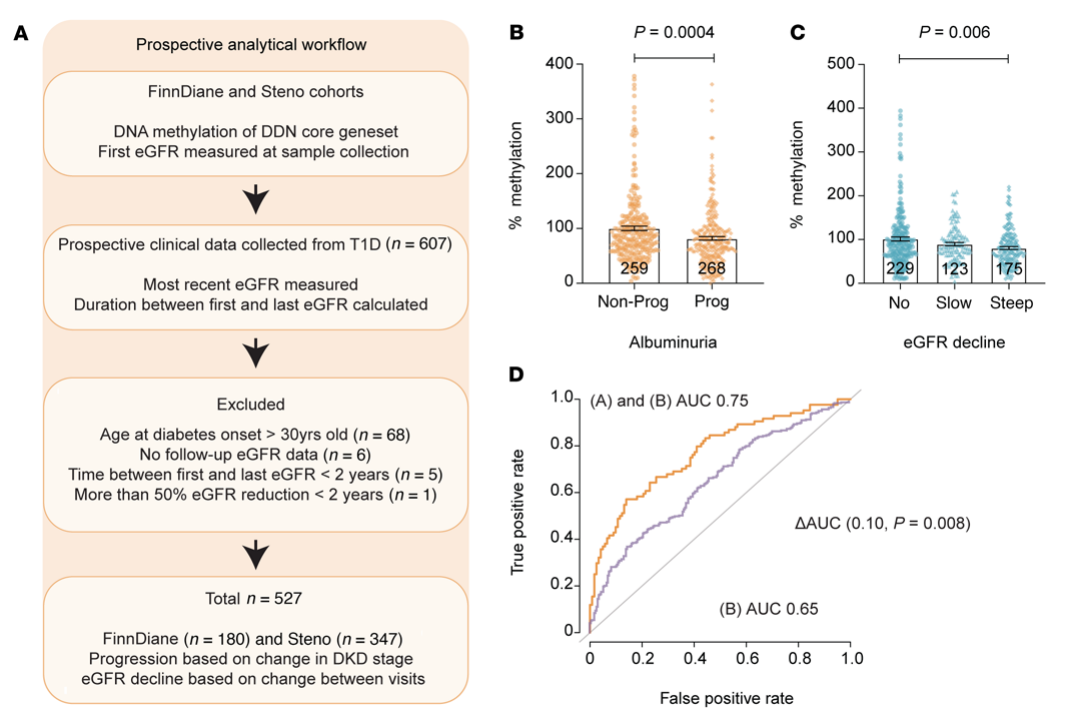
Prospective data analysis of FinnDiane replication and PROFIL validation cohorts. (**A**) Schema of prospective data analysis in FinnDiane and PROFIL cohorts — data were obtained prospectively to evaluate disease progression by also including follow-up eGFR from 527 individuals with T1D. (**B**) Reduced DNA methylation is a feature of albuminuria progression. DNA methylation analysis of genes associated with the DDNs, including *MTOR*, *RPTOR*, *IRS2*, *COL1A2*, *TXNRD1*, *LCAT*, and *SMPD3*. Samples from these T1D cohorts were classified as nonprogressors and progressors based on change in albuminuria stages (Normo to Micro; Micro to Macro; Macro to ESRD). Changes in DNA methylation for the different groups are presented as bar graphs (combined core genes for each sample in different groups). Error bars represent SEM, and the statistical significance was calculated by comparing nonprogressors and progressors using the Mann-Whitney *U* test. (**C**) Reduced DNA methylation is a feature of eGFR decline. DNA methylation analysis of the core genes in the FinnDiane and PROFIL cohorts based on eGFR decline in slope. eGFR decline is defined as the calculated estimated glomerular filtration slope by comparing the difference in the first (with matching DNA methylation readout) and last eGFR measurements as an index of follow-up time in years. No decline is defined as an eGFR slope of –1 mL/min/1.73 m^2^ or greater. Slow decline is defined by an eGFR slope of greater than –3 and less than –1 mL/min/1.73 m^2^. Steep decline is defined by an eGFR slope of less than –3 mL/min/1.73 m^2^. Error bars represent SEM and the significance was calculated by comparing no decline with slow and steep decline groups using the Mann-Whitney *U* test. Reduced DNA methylation associates with steep eGFR decline (*P* = 0.006). (**D**) DNA methylation index improves prediction of eGFR decline. ROC plot shows the AUC score for combined clinical factors (DM duration, HbA1c, UACR, smoking, and systolic blood pressure) 0.65, *P* = 4.08 × 10^–7^ (green). The inclusion of DNA methylation index improves the AUC score of combined clinical factors from 0.65 to 0.75 (*P* = 7.75 × 10^–7^) in predicting eGFR decline in the FinnDiane and PROFIL cohorts (Δ AUC 0.10; *P* = 0.008). The model for (A) combined core gene methylation and (B) clinical factors reports the combined result of gene methylation with individual covariates and shown in Table 1. *P* values were processed using bootstrap in R.

To build on the initial cross-sectional studies, which cannot specifically assess the trajectory of disease progression, prospective analyses of the relationship between baseline methylation and subsequent phenotypic changes were used to predict DN progression. We integrated prospective clinical data from the FinnDiane replication cohort (*n* = 180) combined with the validation registry from Denmark (PROFIL study *n* = 347), consisting in total of 527 individuals (Figure 6A). Reduced core gene methylation was associated with albuminuria progression, as shown in Figure 6B, with mean values of 99.98% for nonprogressors versus 81.76% for progressors. Furthermore, steep eGFR decline in the FinnDiane and PROFIL cohorts show reduced core gene methylation, as shown in Figure 6C, with mean values of 100% for no decline, 89.49% for slow decline, and 80.7% for steep decline. The predictive value of this methylation index was also examined using receiver operating characteristics (ROC). Table 1 shows core gene methylation and eGFR decline (AUC score) associated with diabetes duration, baseline HbA1c (mmol/mol), smoking, and systolic blood pressure (SBP). Figure 6D illustrates the prognostic value of the methylation index. The AUC score for the combined clinical features (DM duration, HbA1c, UACR, smoking, and SBP) was 0.65 (*P* = 4.08 × 10^–7^) and the inclusion of methylation improved the AUC score to 0.75 (*P* = 7.75 × 10^–7^, Δ AUC 0.1; *P* = 0.008). These results suggest methylation scores can improve the estimation of eGFR decline.

### Clinically relevant hyperglycemia influences methylation-dependent expression.

**Figure 7:**
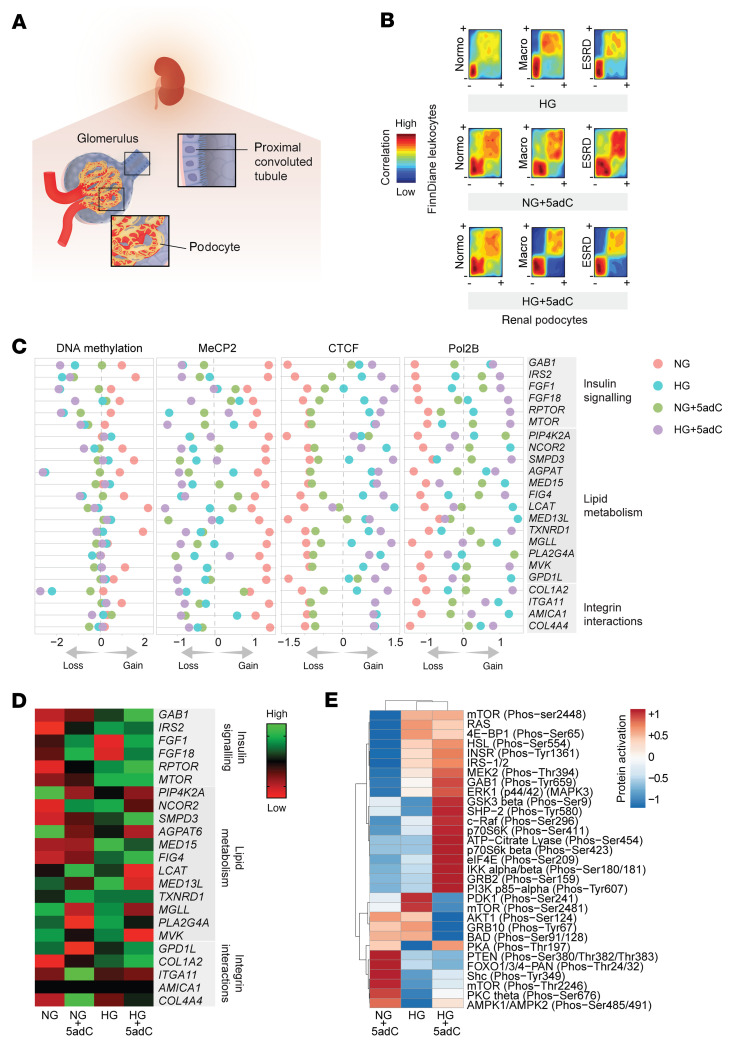
Integration of FinnDiane methylation and renal epigenomic indices. (**A**) Renal cell types (podocytes and proximal convoluted tubule) assessed for DNA methylation mediated expression. (**B**) Reduced leukocyte methylation is a feature of hyperglycemia-induced podocyte changes. Comparison of differential methylation (~80,000 DMRs) in FinnDiane with human podocytes using rank-versus-rank analysis. Density plots show regions with gain (+) and loss (–) of DNA methylation in the FinnDiane discovery cohort (*y* axis) compared with human podocytes (*x* axis). Podocyte methylation sequencing data derived from 3 biological experiments (*n* = 3 per group) that were maintained in NG (physiological glucose control), NG + 5adC (physiological glucose including 3-day treatment with 5-aza-2′-deoxycytidine), HG (15 days high glucose), and HG + 5adC (15 days high glucose including 3-day treatment with 5adC). Similar pattern observed between differential methylation in FinnDiane versus human podocytes stimulated by HG and/or 5adC. (**C**) Diabetic nephropathy is associated with pathways regulated by DNA methylation in FinnDiane leukocytes and human podocytes. Annotated gene name; DNA methylation intensity - scaled (–2, relative methylation loss; +2, relative methylation gain), showing relative gene methylation of human podocytes maintained in NG, NG + 5adC, HG, and HG + 5adC. ChIP-seq data show MeCP2, CTCF, and Pol2B binding intensities relative to inputs (–1, low binding; +1, high binding). (**D**) mRNA expression levels associated with the progression of diabetic nephropathy. These genes are involved in pathways including insulin signaling, lipid metabolism, and integrin-cell interactions. Log_2_ scale — relative expression (–1, reduced; +1, elevated). (**E**) Profiling of phosphoprotein activation in human podocytes (relative to control, NG) using antibody array for the insulin signaling pathway. Proteins (219) were assessed using site-specific and phospho-specific antibodies (*n* = 2), NG, NG + 5adC, HG, and HG + 5adC. Relative protein activation is normalized to control (NG). Heatmap rows and columns are clustered using correlation distance and average linkage.

The relevance of leukocyte methylation was also assessed in the human podocyte (Figure 7A), a glomerular cell considered to play a key role in the pathogenesis and progression of albuminuria, including diabetes (32, 33). We created a model of chronic high glucose (HG) using podocytes and examined DNA methylation–mediated gene expression, including phosphoprotein activity (Supplemental Figure 7A). To determine whether methylation regulates determinants implicated in insulin signaling, lipid metabolism, and fibrosis, human podocytes were also exposed to 5-aza-2′-deoxycytidine (5adC), a pharmacological inhibitor of DNA methylation (34). Reduced leukocyte DNA methylation — a feature of the Scandinavian and Asian cohorts — was confirmed in podocytes subjected to clinically relevant hyperglycemia (Figure 7B). Because DNA methylation is also specifically recognized by protein readers, we also assessed the binding of MeCP2, CTCF, and Pol2B using ChIP-seq and observe a direct regulatory association in podocytes exposed to HG and/or 5adC (Supplemental Figure 7B).

Next, we examined the 23 core genes defined from our cohort studies that were implicated in the regulation of insulin signaling, lipid metabolism, and fibrosis in human podocytes (Figure 7C). Reduced DNA methylation was inversely correlated with a gain of CTCF and Pol2B binding following HG and the pharmacological inhibition of DNA methylation by 5adC. We also assessed mRNA levels of the 23 core genes in human podocytes to show that HG and/or 5adC regulate gene expression (Figure 7D). Furthermore, we observed that the phosphorylation activity of 30 insulin signaling proteins, including mTOR and IRS1/2, by protein array was influenced by DNA methylation in human podocytes (Figure 7E and Supplemental Table 11). These results suggest that DNA methylation of the genes associated with the insulin signaling pathway influence mRNA and protein activation (Supplemental Figure 8).

**Figure 8:**
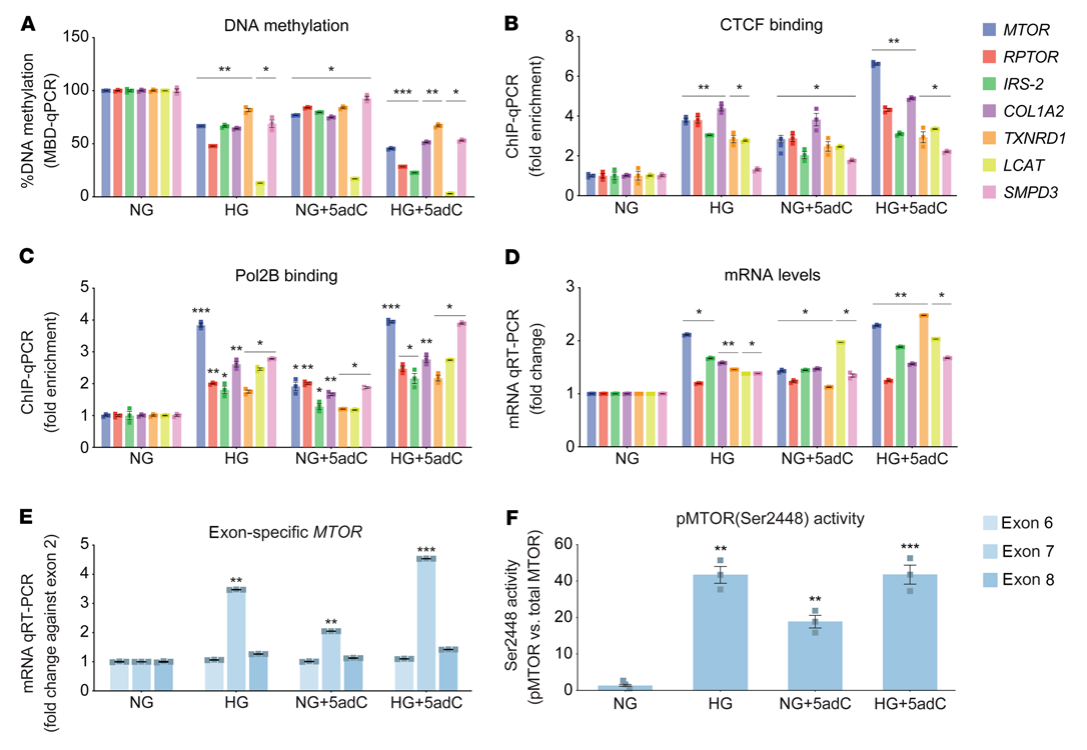
Human podocytes are subject to methylation-mediated changes in response to hyperglycemia. (**A**) DNA methylation of genes associated with progression of diabetic nephropathy using methyl–qPCR of human podocytes exposed to normal glucose (NG) for 15 days and NG for 15 days including 3-day treatment with 5-aza-2′-deoxycytidine (NG + 5adC), high glucose for 15 days (HG), and high glucose including 3-day treatment with 5adC (HG + 5adC). Significance was calculated by comparing NG vs. HG, NG vs. NG + 5adC, and NG vs. HG + 5adC using 2-tailed Student’s *t* test (*n* = 3). (**B**) CTCF and (**C**) Pol2B binding was assessed in podocytes using ChIP-qPCR and signals normalized to IgG control. Significance was calculated by comparing to NG control using 2-tailed Student’s *t* test (*n* = 3). (**D**) Expression of core genes associated with diabetic nephropathy assessed in human podocytes stimulated by chronic HG and 5adC. qRT-PCR data are shown relative to *H3F3A*. Significance was calculated by comparing NG vs. HG, NG vs. NG + 5adC, and NG vs. HG + 5adC using 2-tailed Student’s *t* test (*n* = 3). (**E**) *MTOR* exon–specific qRT-PCR. Relative expression relative to NG control and *MTOR* exon 2 expression (*n* = 3). (**F**) Quantification of phosphorylated MTOR protein in podocytes exposed to HG and 5adC. Bars represent the relative phosphorylation (Ser2448) of MTOR protein in cells exposed to HG and 5adC detected by Odyssey infrared imaging (*n* = 3). *P < 0.05; **P < 0.01; ***P < 0.001. Error bars are SEM.

Reduced DNA methylation was more pronounced in podocytes exposed to HG and/or 5adC (Figure 8A). At the same genomic regions, CTCF binding was significantly increased (Figure 8B) and closely associated with Pol2B enrichment (Figure 8C). Taken together, these experimental studies imply reduced gene methylation to be inversely correlated with the expression of mRNA levels in hyperglycemic podocytes (Figure 8D). Moreover, experimental studies have shown that CTCF binding pauses Pol2B by altering elongation and causing alternative transcript splicing at sites of DNA methylation (35). We examined pausing at *MTOR* exon junctions 6, 7, and 8 using a specific mRNA assay and confirmed enhanced expression of exon 7 in human podocytes (Figure 8E). Moreover, 5adC and HG increased exon 7 expression. These studies are consistent with the idea that reduced methylation could influence *MTOR* splicing. We therefore assessed phosphorylated MTOR (p-MTOR) at Ser2448 in human podocytes. When compared with physiological glucose conditions (NG), phosphorylation of Ser2448 was increased by HG and 5adC (Figure 8F). These results were supported by the primary findings of a phosphoprotein array (Figure 7E and Supplemental Figure 8).

### Reduced DNA methylation influences core gene expression in proximal convoluted tubules, including M1 macrophages.

**Figure 9:**
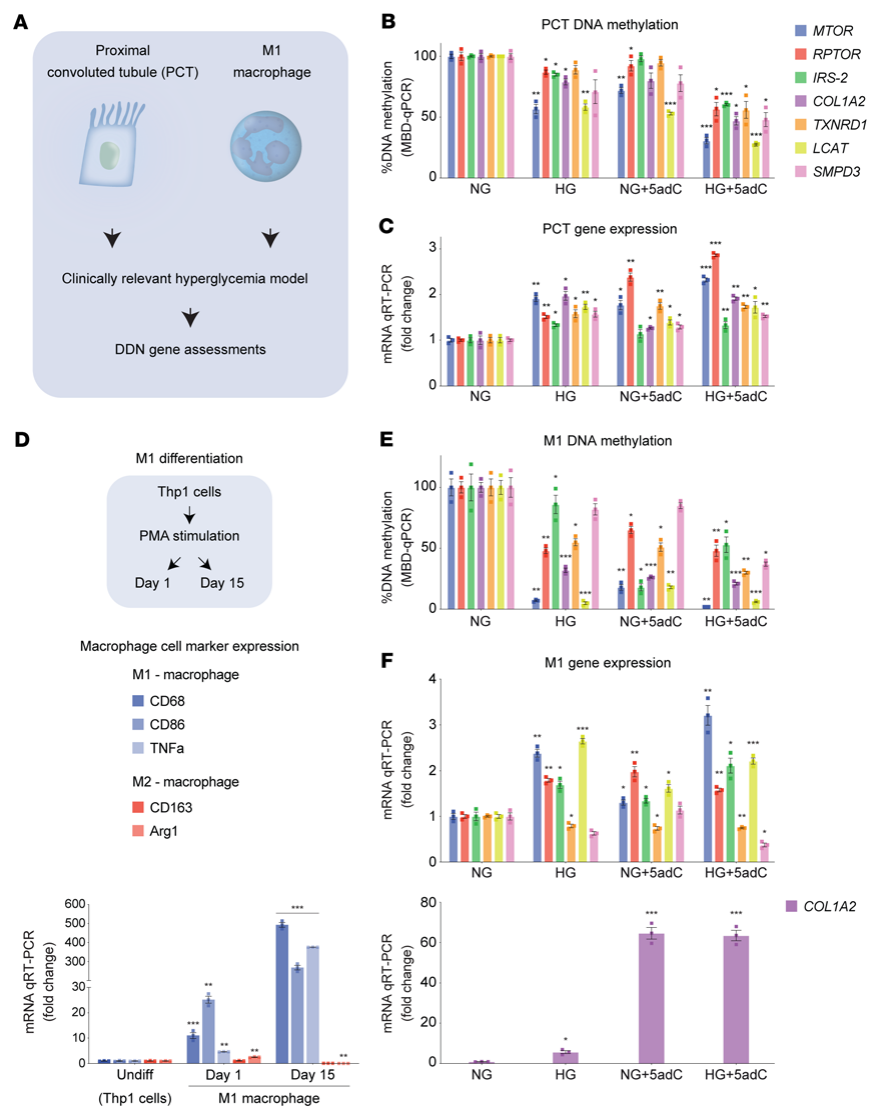
Hyperglycemia influences the DDNs in proximal tubule cells and macrophages. (**A**) Overview of experiments: culture conditions and experimental procedures used to assess core gene methylation and mRNA expression. Human proximal convoluted tubule cells and M1 macrophages (THP-1^+^ monocyte–derived) were cultured in physiological glucose conditions, high glucose (HG), and 5-aza-2′-deoxycytidine (5adC). (**B**) *MTOR*, *RPTOR*, *IRS2*, *COL1A2*, *TXNRD1*, *LCAT*, and *SMPD3* were assessed using methyl–qPCR in PCT cells exposed to normal glucose (NG) for 15 days or NG for 15 days including 3 days with 5adC (NG + 5adC), HG for 15 days (HG), and HG including 3-days with 5adC (HG + 5adC) (*n* = 3). (**C**) mRNA levels of core genes assessed in PCT cells stimulated by chronic HG and 5adC. qRT-PCR data are shown relative to *H3F3A* (*n* = 3). (**D**) Macrophage differentiation from THP-1^+^ monocytes treated with phorbol-12-myristate-13-acetate (PMA) for 1 day and 15 days. Expression of macrophage-specific markers *CD68*, *CD86*, and *TNFA* (M1 macrophages) and *CD163* and *ARG1* (M2 macrophages) assessed by qRT-PCR. Data are shown relative to *H3F3A* (*n* = 3). (**E**) Methylation analysis of core genes in M1 macrophages (differentiated THP-1 day 15) exposed to HG and/or 5adC (*n* = 3). (**F**) mRNA levels of core genes assessed in M1 macrophages stimulated by chromic HG and 5adC. Data are shown relative to *H3F3A*. Significance was calculated using 2-tailed Student’s *t* test by comparing NG vs. HG, NG vs. NG + 5adC, and NG vs. HG + 5adC (**B**, **C**, **E**, and **F**) or by comparing undifferentiated vs. day 1 and undifferentiated vs. day 15 (**D**). *P < 0.05, **P < 0.01, ***P < 0.001. Error bars are SEM.

Since macrophage infiltration is associated with fibrosis and renal tubular injury (36, 37), we prepared human cell models of HG and/or 5adC using proximal convoluted tubules (PCTs) and M1 macrophages (Figure 9A). We observed that reduced core gene methylation in PCTs (Figure 9B) was inversely associated with mRNA levels (Figure 9C). We also assessed the role of core gene methylation in differentiated human M1 macrophages (Figure 9D). Consistent with renal podocytes and PCTs, reduced DNA methylation (Figure 9E) was inversely correlated with mRNA levels in M1 cells (Figure 9F). These results suggest that observed changes in DNA methylation are likely to be coordinated and the cell type–specific patterns appear to be consistent for renal pathways that are associated with the progression of DN.

### Insulin pathway genes are transcriptionally regulated in vascular endothelial cells.

**Figure 10:**
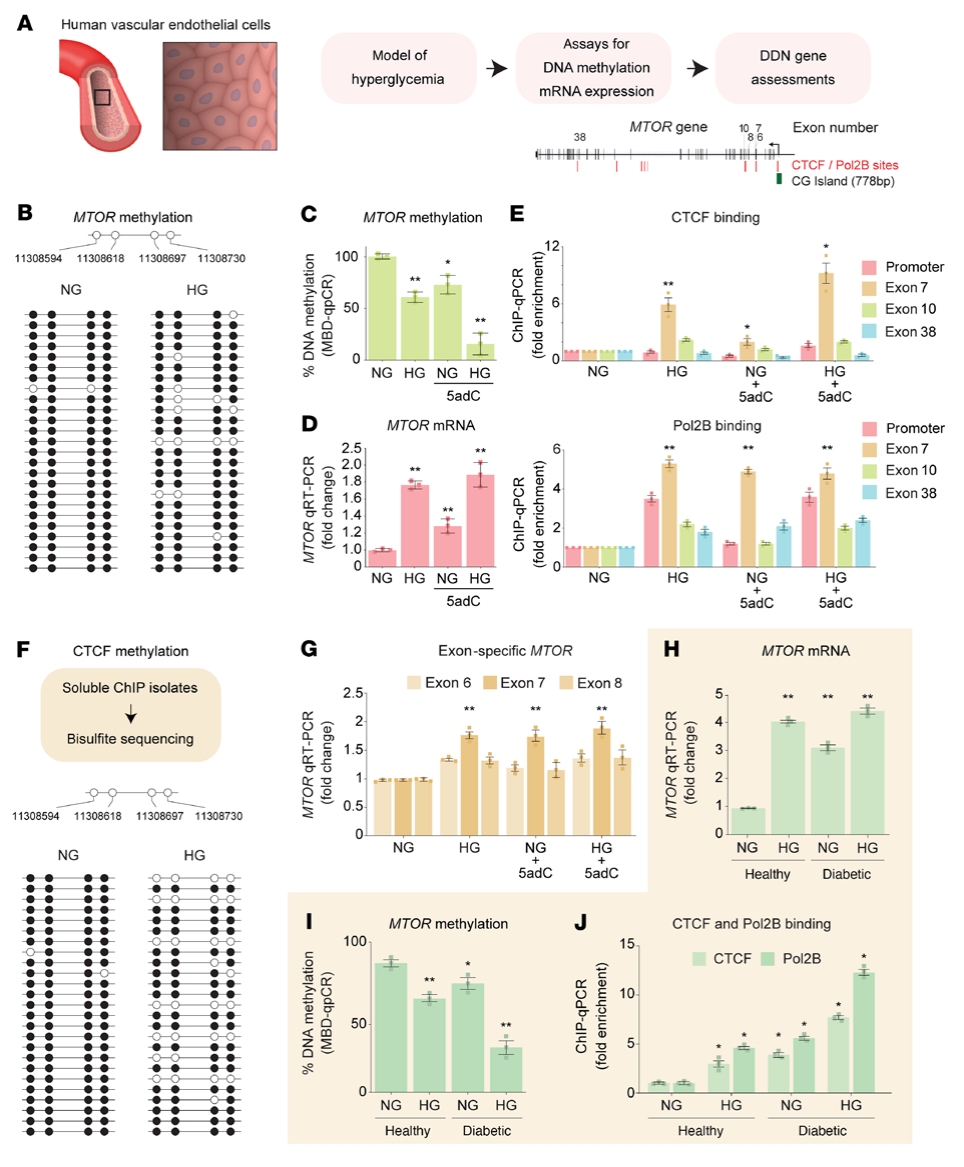
Methylation-mediated gene expression in human vascular endothelial cells. (**A**) Model of hyperglycemia using primary human vascular endothelial cells derived from nondiabetic and T1D individuals. (**B**) *MTOR* bisulfite sequencing. Data are represented as a single DNA molecule from 1 sample from each group. Open circles, unmethylated CG; solid circles, methylated CG. (**C**) *MTOR* methylation analysis using methyl–qPCR. (**D**) *MTOR* mRNA levels in human endothelial cells stimulated by chronic HG and 5adC. qRT-PCR data are shown relative to *H3F3A*. Significance in **C** and **D** was calculated by comparing normal glucose (NG) vs. high glucose (HG), NG vs. NG + 5-aza-2′-deoxycytidine (5adC), and NG vs. HG + 5adC (*n* = 3). (**E**) Schematic of DMRs validated in the FinnDiane cohort overlapping the CTCF and Pol2B binding motifs proximal to *MTOR* exon 7. CTCF and Pol2B binding was assessed by ChIP-qPCR and signals were adjusted to an IgG antibody control. Regions of interest amplified are the CTCF binding sites on *MTOR*. Significance was calculated by comparing to NG control (*n* = 3). (**F**) CTCF ChIP assay combined with bisulfite sequencing upstream of exon 7 of the *MTOR* gene. Open circles, unmethylated CG; solid circles, methylated CG. (**G**) *MTOR* exon–specific qRT-PCR assay in human vascular endothelial cells. mRNA levels reported relative to NG (*n* = 5). (**H**) *MTOR* qRT-PCR data from primary human aortic endothelial cells isolated from healthy and T1D individuals (*n* = 3). (**I**) *MTOR* methylation analysis in primary endothelial cells using methyl-qPCR. DNA methylation was further reduced in diabetic cells exposed to HG. (**J**) CTCF and Pol2B binding was reduced in hyperglycemic conditions. Significance in **I** and **J** was calculated by comparing healthy vs. healthy + HG, diabetic vs. diabetic + HG, and healthy vs. diabetic + HG (*n* = 3). Experiments were performed on cells from passages 4 to 7. *P < 0.05, **P < 0.01 by 2-tailed Student’s *t* test. Error bars are SEM.

To determine the generalizability to other cell populations directly exposed to hyperglycemia and vulnerable to diabetic complications, we examined *MTOR* regulation in human vascular endothelial cells (Figure 10A). We observed reduced methylation at 4 CG sites of the *MTOR* gene using bisulfite sequencing in hyperglycemic cells (*P* = 0.044; Figure 10B). Reduced DNA methylation (Figure 10C) was also inversely correlated with *MTOR* mRNA levels (Figure 10D). ChIP assays revealed that increased CTCF and Pol2B binding was associated with reduced DNA methylation (Figure 10E). Next, we developed a highly specific CTCF ChIP assay that combined an additional step to map DNA methylation upstream of exon 7 of the *MTOR* gene. Bisulfite sequencing of CTCF-bound chromatin showed a significant reduction in *MTOR* methylation in hyperglycemic endothelial cells (*P* = 0.035; Figure 10F). Consistent with these observations, we also report elevated *MTOR* exon 7 mRNA levels (Figure 10G).

Primary human cells have functional properties that can be used to assess signaling pathways and have emerged as important ex vivo models to study preclinical mechanisms (38). To investigate whether chronic hyperglycemia increases *MTOR* expression, we used primary human aortic endothelial cells (HAECs) derived from T1D individuals. *MTOR* expression was elevated in diabetic HAECs when compared with nondiabetic cells (Figure 10H). In our results, *MTOR* mRNA levels are significantly increased in hyperglycemic conditions. Indeed, hyperglycemia further reduced *MTOR* methylation in diabetic HAECs (Figure 10I) and associated with enriched CTCF and Pol2B binding on the *MTOR* gene (Figure 10J). These experimental results in vascular endothelial cells are consistent with human renal and macrophage cell types and leukocyte-derived observations derived from the Finland, Denmark, Hong Kong, and Thailand T1D cohorts. Taken together, these findings in tissue-specific cells show that hyperglycemia converges on major insulin, lipid, and fibrosis pathways by DNA methylation to regulate the expression of core genes that consist of but are unlikely to be limited to *MTOR*, *RPTOR*, *IRS2*, *TXNRD1*, *LCAT*, *SMPD3*, and *COL1A2*.

## Discussion

In this study, we have identified reduced methylation to be associated with the progression of DN. We believe this is novel for 2 reasons. First, to our knowledge there are no comprehensive DNA methylation data that characterize the biologic foundation of DN progression. While there are many ways to assess DNA methylation, initial forays into diabetes were made using array hybridization and endonuclease digestion assays (39, 40). In this study, to interpret the FinnDiane methylome, we considered that the genome coverage of various methodologies was critical to understanding the influence of methylation on gene function. A popular method using the BeadChip array characterizes approximately 10^3^–10^5^ CG sites, whereas methylation sequencing offers higher genome coverage in the range of 10^6^ to 10^7^. Another clear indication from the current study is that the overlap of methylation sites identified by sequencing on the 450K array was close to 23% and for the 850K array was close to 35%. Thus, CG site coverage was critical to understanding how DNA methylation outside promoter regions could regulate gene expression. The sequencing approach adopted in this study reduces the technical limitations associated with array composition. While it is known that methylation can silence gene expression, our data show these changes can also be associated with the progression of DN and were not restricted to promoters. This is also consistent with the hypothesis that the majority of DNA methylation of the adult vertebrate cell is located in gene bodies and a much stronger indicator of gene expression (41).

**Figure 11:**
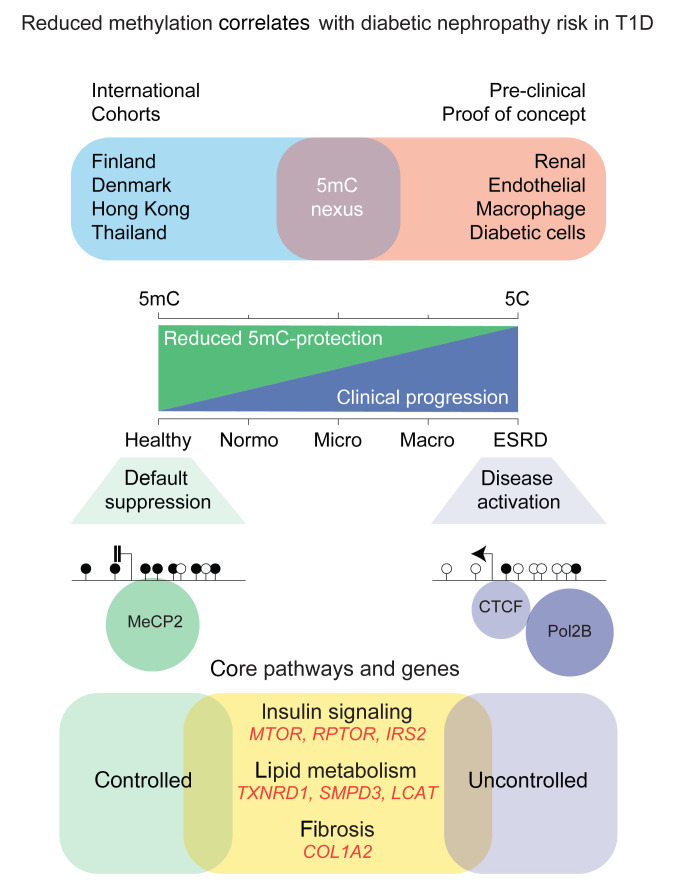
Reduced methylation correlates with diabetic nephropathy risk in type 1 diabetes. DNA methylation–dependent regulation of genes implicated in pathways that are clinically relevant to the progression of diabetic nephropathy. Erosion of DNA methylation is associated with the loss of protection and the activation of core pathways associated with DN risk in type 1 diabetes.

Second, and important clinically, this study to our knowledge is the first to demonstrate DNA methylation to be functionally associated with the regulation of *MTOR*, *RPTOR*, *IRS2*, *COL1A2*, *TXNRD1*, *LCAT*, and *SMPD3* gene expression. Indeed, these core genes were validated in the Scandinavian and Asian T1D cohorts and mechanistically examined using clinically relevant hyperglycemia in human cells. We propose the changes in methylation were neither random nor limited to 1 gene, but rather, organize the epigenome of the diabetic and hyperglycemic cell that converge by DNA methylation at multiple sites to coordinate expression of core pathways (Figure 11). The most direct mechanism by which methylation could influence gene expression is to regulate the binding of transcriptional machinery at consensus binding sites (42). Rather than focusing on a limited number of genes, which is neither an effective nor attractive mechanism of gene regulation, our study approach and experimental observations suggest DNA methylation could coordinate core pathways at consensus binding sites that are consistent with the polygenic nature of DN (43). The assembly of gene-activating events by CTCF and Pol2B helps to partly explain the high affinity for unmethylated regions in T1D individuals with progressive renal disease.

How DNA methylation could influence multiple pathways in diabetes remains poorly understood. To address this, we assayed the ability of specific transcription factors that assemble on CG sites sensitive to methylation. With 60% to 90% of the adult vertebrate cell methylated at CG sites, only a fraction of these can be assessed using BeadChip array (44) and this formed the rationale behind our methyl-seq approach. Furthermore, rather than querying a limited set of methylation sites, the reverse question — which biological processes were prime methylation candidates — was arguably a productive consideration. This strategy had the added advantage of being independent of probe set design. To illustrate this point, we compared methylated genes derived from our sequencing approach with published studies and observed poor convergence with BeadChip array probes. We found that 491 published CG sites are located within a 100-kb genomic window from any DMRs identified by sequencing. This number is reduced to 56 CG sites in a 10-kb window and 4 CG sites that directly overlap (30, 45–48). We consider the estimation of differential methylation by array probes and the determination by methyl-seq to be too large to carry real meaning for predicting DN. For example, when we assessed the genomic positions of core genes belonging to the DDNs with those published using BeadChip arrays, we found that the nearest CG site was mapped 118 kb away from the DMR annotated to *RPTOR*.

The most direct mechanism whereby DNA methylation and protein readers like MeCP2 may regulate gene expression is by altered binding of CTCF and Pol2B (42). The loss of methylation-dependent MeCP2 binding was closely associated with transcriptional regulation of core genes. While it is known that insulin resistance contributes to the progression of renal disease (49, 50), we observed that reduced DNA methylation of insulin signaling genes closely corresponded to gene-activating events. How methylation influences chromatin structure and gene function remains poorly characterized. Strongly conserved but of unknown function in DN, CTCF is a 11–zinc finger DNA-binding protein (51) sensitive to DNA methylation (52) and functions as a gene insulator (53). We observed that CTCF and Pol2B were enriched at sites of reduced methylation in the peripheral blood derived from T1D individuals with DN, whereas DNA methylation was more abundant at the same gene sequences in individuals with diabetes with improved or normal renal function. Nowhere is this complexity more evident than in pathway activation such as insulin signaling, lipid metabolism, and fibrosis.

While insulin signaling and fibrosis were previously linked with the development of DN (28, 33), recent studies have shown crosstalk with lipid metabolism (33, 54). Indeed, reduced DNA methylation was a prominent signature of lipid metabolism genes such as *LCAT* and *SMPD3*. Fibrosis is also a hallmark of DN (55, 56) characterized by the accumulation of extracellular matrix components and influenced by integrin interactions (57). Furthermore, GWAS of the T1D Nephropathy Collaborative Research Initiative of European descent identified *COL4A3* as one of the predisposing loci based on albuminuria and renal function (15). We observed a convergence of the biological mechanisms associated with integrin interactions, showing differential methylation of collagen gene family members such as *COL4A1*, *COL4A2*, and *COL4A4* in the high- and very-high-risk FinnDiane groups. This is consistent with the validation of *COL1A2* in the replication cohorts originating from Denmark, Hong Kong, and Thailand.

The findings of this study also confirm reduced DNA methylation of *TXNIP*, an important glucose-sensing gene associated with metabolic memory in podocyte injury (30) that is epigenetically regulated (21, 58, 59). We examined our FinnDiane methyl-seq with data from the DCCT BeadChip array, which compared mean HbA1c with diabetic complications (21). We observed no overlap with core gene methylation. Despite these differences, we did observe overlapping CG sites on other DMGs using sequences that exist on the BeadChip array. They include functional connectivity with blood glucose in diabetes: *TXNIP* (58–61), *TTC7B* (62), *ANKS3* (63), chromatin determinants *ARID5B* and *CUX1* (48), islet autoantigen *PTPRN2* (64), and metabolic regulators *HK1* (65), *PFKFB3* (66), and *NCOR2* (67).

This research has limitations. While our study shows close correlation between core gene methylation and DN risk in T1D using independent cohorts, the relevance to other populations is warranted. Reduced eGFR is more common among US Hispanics and African Americans and a recent EWAS identified trans-ethnic and ethnicity-specific differentially methylated positions from whole blood (68). The cross-sectional studies described here using independent Asian T1D registries show comparable methylation loss, as demonstrated by the meta-analysis of core genes. Furthermore, we observed a strong correlation of the effect estimates for core gene methylation in the high- and very-high-risk DN groups. The critical risk genes identified include *MTOR*, *RPTOR*, *COL1A2*, and *LCAT*, suggesting probable generalizability in the Scandinavian and Asian cohorts.

While changes in gene methylation have been shown to vary between populations (69, 70), a recent meta-analysis of diabetic kidney disease stratified for eGFR and UACR involving 33,605 participants of European, African American, Hispanic, and South Asian ancestry showed methylation to be closely associated in diverse ethnic cohorts (71). Furthermore, that study assessed common SNPs (minor allele frequency >0.05) in or within 50 bp of the replicated CG sites and showed no common traits for kidney damage. Nevertheless, further studies prioritizing T1D risk loci in diverse at-risk populations may help elucidate ethnic differences in DN prevalence, health outcomes, and therapeutic response. Further work is also required to understand whether the loss of methylation at core genes identified in the multinational cohorts could serve as a biomarker of clinical risk for the development, progression, or response to treatment of DN in diverse ethnic populations. While blood and renal biopsy pairing studies are beyond the scope of the primary findings presented here, such biomarker studies should be considered. Initial studies of FFPE renal biopsies (American University of Beirut Medical Center) show that reduced core gene methylation was inversely associated with UACR in DN (data not shown).

The influence on gene expression examined by mRNA-seq highlights a tight association with DNA methylation for the majority of core genes that were experimentally confirmed for insulin signaling genes and phosphoprotein activity, including *MTOR* (Ser2448), *IRS2*, and *GAB1* (Tyr580). While the data suggest high-confidence networks for methylation-mediated gene expression, there were biological exceptions. For example, mRNA levels of FGF family members such as *FGF1* and *FGF18* were not correlated with decreased gene methylation in podocytes stimulated by chronic hyperglycemia. Indeed, the inability to reactivate *FGF18* suggests hyperglycemia may not effectively influence transcription because of differential epigenetic modification of H3K27me3 by EZH2 (72). Despite these differences, comparison of human podocyte mRNA-seq data generated in this study closely correspond with published observations of the human DN transcriptome. High confidence was observed for core gene expression in PTCs and vascular endothelial cells. This is emphasized in data derived for the transcriptome analysis of human diabetic kidney disease (73), showing 16 of the 23 DDN genes we have identified in human podocyte experiments. More recent studies examining human diabetic renal cells by scRNA-seq (74) show some similarities in the expression of core genes in podocytes and PTCs in this study.

In summary, we propose that reduced methylation could represent diminished protection of core genes that may associate with DN progression. Furthermore, the same methylation indices derived from peripheral blood could possibly improve the predictive scores of eGFR decline and thus add to current clinical modeling within the context of DN.

## Methods

Additional methods are described in the Supplemental Methods.

### DN definition.

DN progression was measured using albumin excretion rate (AER) and serum creatinine to calculate eGFR in the FinnDiane study.

### FinnDiane study.

The discovery cohort in this study was grouped based on the urine AER in 10 T1D individuals with normoalbuminuria (Normo), 9 with macroalbuminuria (Macro), and 6 with ESRD. ESRD was defined as individual treated by dialysis or having received a kidney transplant. The majority of Normo T1D individuals (7 out of 10) included in this study were free of complications. However, 3 Normo individuals and 4 Macro individuals had severe retinopathy. Clinical characteristics of case and control groups are described in Supplemental Table 1. In addition to the discovery cohort initially sequenced, a larger subset of FinnDiane was included for validation experiments, including T1D with Micro. In total, the FinnDiane validation cohort consisted of 19 individuals without diabetes and 277 individuals with T1D divided into 65 Normo, 73 Micro, 66 Macro, and 73 ESRD individuals undergoing dialysis (Supplemental Table 5).

### Replication cohorts.

A cohort of 670 adults with T1D were recruited between 2009 and 2011 from the outpatient clinic at Steno Diabetes Center Copenhagen, defined as the PROFIL cohort. The details of the selection process have previously been described (75). A total of 405 participants with diabetes duration of 20 years or more with a wide range of albuminuria were included in our study. The UACR was calculated by spot urine test; hence, 170 Normo, 110 Micro, and 125 Macro individuals. A control group of 40 individuals without diabetes was also included in the study for comparison with individuals with diabetes (Supplemental Table 6).

The Hong Kong Diabetes Register was established in 1995 at the Prince of Wales Hospital, the teaching hospital of the Chinese University of Hong Kong, as part of a quality improvement program. Details of the cohort have been previously reported (76). Among 10,129 individuals enrolled into the registry, there were 374 with T1D and 77 of the participants were selected for this study. From the community-based healthy cohort, 30 nondiabetics were included in this study as controls (Supplemental Table 7).

A cross-sectional study of Thai individuals with T1D and disease duration of 25 years or more managed at the Theptarin Hospital, Bangkok, Thailand was performed. To be enrolled in this study, an individual must have had a clinical diagnosis of T1D made by a diabetologist on the basis of their clinical course and random plasma C-peptide levels of 0.6 ng/mL or lower. Written informed consent was obtained from participants prior to study inclusion (Supplemental Table 8).

### Methyl-seq.

Methyl-binding-domain enrichment sequencing was used to investigate DNA methylation in individuals with T1D and healthy controls from the FinnDiane discovery cohort. Genomic DNA (gDNA) was extracted from venous blood (white blood cell layer) using PureGene, Gentra method (Gentra systems).

### Bioinformatic analysis for methyl-seq.

Sequencing files were examined for quality assurance. Fastx quality trimmer (http://hannonlab.cshl.edu/fastx_toolkit/) was used to remove low-quality bases from the 3′ end of the sequence read at a base quality threshold of 20. Reads shorter than 40 bp were discarded. Sequenced reads were aligned to the human reference genome (hg19) using BWA-MEM (77) with default alignment parameters and sequence alignments for each contig were counted (Supplemental Table 2).

### KDIGO classification.

KDIGO is the most recent chronic kidney disease classification and according to KDIGO, chronic kidney disease is defined as abnormalities in the kidney structure and function, present for more than 3 months, with implications for health (78, 79). Supplemental Table 9 categorizes GFR (G1–G5) and albuminuria (A1–A3). Based on the KDIGO classification endorsed by the American Diabetes Association, we categorized cohorts into the 4 groups: low risk, moderate risk, high risk, and very high risk.

### FinnDiane SNP genotyping.

DNA methylation was not significantly associated with the 14 SNPs detected in the 7 core genes. The SNP allele frequencies were also similar in the DN groups (Supplemental Table 12).

### Human cell culture.

Conditionally immortalized human podocytes were maintained at 33°C in RPMI 1640 medium (GIBCO, 11879-020; made up to 5.5 mM glucose) with 10% fetal bovine serum (FBS), 1% penicillin/streptomycin (GIBCO, 15140122), and 1× insulin-transferrin supplement (Life Technologies, 41400-045) as previously described (80). Podocytes were then differentiated for 10 days at 37°C in RPMI (5.5 mM) containing 2% FBS and 1% penicillin/streptomycin. Following differentiation, human podocytes expressing nephrin and podocin underwent glucose treatment.

### Core DDN gene detection by methyl-qPCR assay.

We performed weighted meta-analysis using core gene methylation data obtained from 978 individuals that comprise the FinnDiane, PROFIL, Hong Kong, and Theptarin cohorts (Supplemental Table 10). This meta-analysis generated the median difference in percentage methylation, comparing the low-risk group with moderate-risk, high-risk, and very-high-risk groups, respectively. The *P* value reported for each cohort was calculated by Mann-Whitney *U* test. We used R packages metamedian (81) and survcomp (version 1.22.0) (82) to combine core gene methylation in the diabetes registries. Statistical significance for the meta-analysis was calculated by Fisher’s combined probability test.

### Data access.

The raw data have been deposited in the NCBI Gene Expression Omnibus database (GEO GSE77011: Mapping the human diabetic methylome).

### Primers.

All primer sequences used for methyl-qPCR, ChIP-qPCR, and gene expression experiments are included in Supplemental Table 13.

### Statistics.

For clinical studies, significance was determined by comparing T1D with no complications to T1D with DN using the Mann-Whitney *U* test. For glucose stimulation studies, statistical significance and *P* values were calculated by 2-tailed Student’s *t* tests by comparing normoglycaemia groups using Graphpad Prism. *P* values of less than 0.05 were considered significant.

### Study approval.

All participants provided written informed consent prior to the study. All data were collected in accordance with the Declaration of Helsinki and the study was approved by the local ethics committee at each study center. For the replication cohorts, the Ethics Committee E, Region Hovedstaden, Denmark, approved the original and follow-up research protocol. Written informed consent was obtained from all participants for the original study and the biobank. The Thai T1D study was approved by the Institutional Review Boards at Theptarin Hospital (EC 09/2018).

## Author contributions

IK performed the investigation, validation, and data visualization. IK, HK, SM, JO, MZ, AK, HR, AJ, KAH, RN, AE, and AEO contributed to data curation, software, and methodology. AS, C Forsblom, C Fogarty, and MCT provided resources and methodology for the discovery cohort. GJ, AOYL, HML, YH, RCWM, and JCNC provided the Hong Kong replication samples. YT, SN, and TH provided the Thai replication samples. SB, TWH, NT, ECO, and PR provided the PROFIL study replication samples. YT, TH, RCWM, JCNC, MEC, PR, PHG, and AEO contributed to conceptualization, supervision, project administration, and funding acquisition. IK and AEO wrote the original manuscript draft, which was reviewed and edited by all authors.

## Supplementary Material

Supplemental data

## Figures and Tables

**Table 1:**
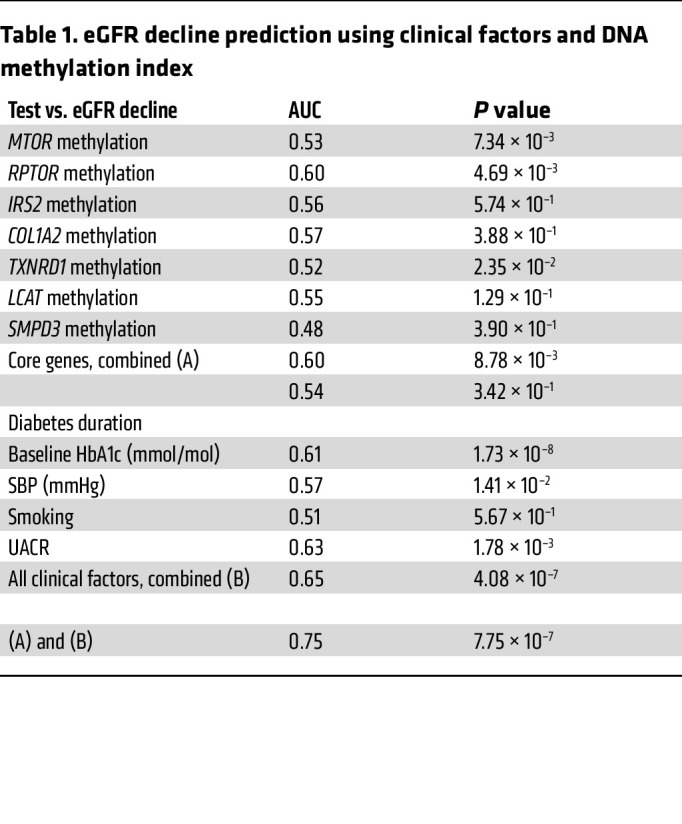
eGFR decline prediction using clinical factors and DNA methylation index

## Version 1 01/12/2023

In-Press Preview

## Version 2 02/15/2023

Electronic publication

## References

[1] Saran R (2017). US Renal Data System 2016 annual data report: epidemiology of kidney disease in the United States. Am J Kidney Dis.

[2] de Boer IH (2011). Long-term renal outcomes of patients with type 1 diabetes mellitus and microalbuminuria: an analysis of the Diabetes Control and Complications Trial/Epidemiology of Diabetes Interventions and Complications cohort. Arch Intern Med.

[3] Diabetes C (2009). Modern-day clinical course of type 1 diabetes mellitus after 30 years’ duration: the diabetes control and complications trial/epidemiology of diabetes interventions and complications and Pittsburgh epidemiology of diabetes complications experience (1983-2005). Arch Intern Med.

[4] Clustering of long-term complications in families with diabetes in the diabetes controlcomplications trial (1997). The Diabetes Control and Complications Trial Research Group. Diabetes.

[5] Quinn M (1996). Familial factors determine the development of diabetic nephropathy in patients with IDDM. Diabetologia.

[6] Seaquist ER (1989). Familial clustering of diabetic kidney disease. Evidence for genetic susceptibility to diabetic nephropathy. N Engl J Med.

[7] Ma RC, Cooper ME (2017). Genetics of diabetic kidney disease-from the worst of nightmares to the light of dawn?. J Am Soc Nephrol.

[8] Sandholm N (2017). The genetic landscape of renal complications in type 1 diabetes. J Am Soc Nephrol.

[9] Thorn LM (2005). Metabolic syndrome in type 1 diabetes: association with diabetic nephropathy and glycemic control (the FinnDiane study). Diabetes Care.

[10] Chaturvedi N (2001). Microalbuminuria in type 1 diabetes: rates, risk factors and glycemic threshold. Kidney Int.

[11] Lindholm E (2001). Classifying diabetes according to the new WHO clinical stages. Eur J Epidemiol.

[12] Amin R (2008). Risk of microalbuminuria and progression to macroalbuminuria in a cohort with childhood onset type 1 diabetes: prospective observational study. BMJ.

[13] Marcovecchio ML (2009). Ambulatory blood pressure measurements are related to albumin excretion and are predictive for risk of microalbuminuria in young people with type 1 diabetes. Diabetologia.

[14] Sandholm N (2012). New susceptibility loci associated with kidney disease in type 1 diabetes. PLoS Genet.

[15] Salem RM (2019). Genome-wide association study of diabetic kidney disease highlights biology involved in glomerular basement membrane collagen. J Am Soc Nephrol.

[16] Laird PW (2010). Principles and challenges of genomewide DNA methylation analysis. Nat Rev Genet.

[17] Sapienza C (2011). DNA methylation profiling identifies epigenetic differences between diabetes patients with ESRD and diabetes patients without nephropathy. Epigenetics.

[18] Bell CG (2010). Genome-wide DNA methylation analysis for diabetic nephropathy in type 1 diabetes mellitus. BMC Med Genomics.

[19] Stefan M (2014). DNA methylation profiles in type 1 diabetes twins point to strong epigenetic effects on etiology. J Autoimmun.

[20] Swan EJ (2015). Distinct methylation patterns in genes that affect mitochondrial function are associated with kidney disease in blood-derived DNA from individuals with type 1 diabetes. Diabet Med.

[21] Chen Z (2020). DNA methylation mediates development of HbA1c-associated complications in type 1 diabetes. Nat Metab.

[22] Rafehi H (2014). Vascular histone deacetylation by pharmacological HDAC inhibition. Genome Res.

[23] Medvedeva YA (2014). Effects of cytosine methylation on transcription factor binding sites. BMC Genomics.

[24] Brorsson CA (2015). Shared genetic basis for type 1 diabetes, islet autoantibodies, and autoantibodies associated with other immune-mediated diseases in families with type 1 diabetes. Diabetes Care.

[25] Consortium EP (2004). The ENCODE (ENCyclopedia Of DNA Elements) Project. Science.

[26] Wang H (2012). Widespread plasticity in CTCF occupancy linked to DNA methylation. Genome Res.

[27] Consortium EP (2011). A user’s guide to the encyclopedia of DNA elements (ENCODE). PLoS Biol.

[28] Su W (2017). Crosstalk of hyperglycemia and dyslipidemia in diabetic kidney disease. Kidney Dis (Basel).

[30] Chen Z (2016). Epigenomic profiling reveals an association between persistence of DNA methylation and metabolic memory in the DCCT/EDIC type 1 diabetes cohort. Proc Natl Acad Sci U S A.

[31] Shah UJ (2019). Differential methylation of the type 2 diabetes susceptibility locus KCNQ1 is associated with insulin sensitivity and is predicted by CpG site specific genetic variation. Diabetes Res Clin Pract.

[32] Toyoda M (2007). Podocyte detachment and reduced glomerular capillary endothelial fenestration in human type 1 diabetic nephropathy. Diabetes.

[33] Fornoni A (2014). Lipid biology of the podocyte — new perspectives offer new opportunities. Nat Rev Nephrol.

[34] Robertson KD (2005). DNA methylation and human disease. Nat Rev Genet.

[35] Shukla S (2011). CTCF-promoted RNA polymerase II pausing links DNA methylation to splicing. Nature.

[36] Zeni L (2017). A more tubulocentric view of diabetic kidney disease. J Nephrol.

[37] Chow FY (2004). Macrophages in streptozotocin-induced diabetic nephropathy: potential role in renal fibrosis. Nephrol Dial Transplant.

[38] Khamaisi M (2016). PKCδ inhibition normalizes the wound-healing capacity of diabetic human fibroblasts. J Clin Invest.

[39] Dedeurwaerder S (2014). A comprehensive overview of Infinium HumanMethylation450 data processing. Brief Bioinform.

[40] Ross JP (2022). Batch-effect detection, correction and characterisation in Illumina HumanMethylation450 and MethylationEPIC BeadChip array data. Clin Epigenetics.

[41] Lou S (2014). Whole-genome bisulfite sequencing of multiple individuals reveals complementary roles of promoter and gene body methylation in transcriptional regulation. Genome Biol.

[42] Ko YA (2013). Cytosine methylation changes in enhancer regions of core pro-fibrotic genes characterize kidney fibrosis development. Genome Biol.

[43] Thomas MC (2015). Diabetic kidney disease. Nat Rev Dis Primers.

[44] Harris RA (2010). Comparison of sequencing-based methods to profile DNA methylation and identification of monoallelic epigenetic modifications. Nat Biotechnol.

[45] Wing MR (2014). DNA methylation profile associated with rapid decline in kidney function: findings from the CRIC study. Nephrol Dial Transplant.

[46] Qiu C (2018). Cytosine methylation predicts renal function decline in American Indians. Kidney Int.

[47] Gluck C (2019). Kidney cytosine methylation changes improve renal function decline estimation in patients with diabetic kidney disease. Nat Commun.

[48] Smyth LJ (2021). Assessment of differentially methylated loci in individuals with end-stage kidney disease attributed to diabetic kidney disease: an exploratory study. Clin Epigenetics.

[49] Hung SC (2015). Metformin use and mortality in patients with advanced chronic kidney disease: national, retrospective, observational, cohort study. Lancet Diabetes Endocrinol.

[50] Lay AC, Coward RJM (2018). The evolving importance of insulin signaling in podocyte health and disease. Front Endocrinol (lausanne).

[51] Phillips JE, Corces VG (2009). CTCF: master weaver of the genome. Cell.

[52] Kanduri C (2000). Functional association of CTCF with the insulator upstream of the H19 gene is parent of origin-specific and methylation-sensitive. Curr Biol.

[53] Lewis A, Murrell A (2004). Genomic imprinting: CTCF protects the boundaries. Curr Biol.

[54] Mitrofanova A (2021). New insights into renal lipid dysmetabolism in diabetic kidney disease. World J Diabetes.

[55] Chen X (2004). Lack of integrin alpha1beta1 leads to severe glomerulosclerosis after glomerular injury. Am J Pathol.

[56] Bon H (2019). Spontaneous extracellular matrix accumulation in a human in vitro model of renal fibrosis is mediated by αV integrins. Nephron.

[57] Pozzi A, Zent R (2013). Integrins in kidney disease. J Am Soc Nephrol.

[58] Parikh H (2007). TXNIP regulates peripheral glucose metabolism in humans. PLoS Med.

[59] De Marinis Y (2016). Epigenetic regulation of the thioredoxin-interacting protein (TXNIP) gene by hyperglycemia in kidney. Kidney Int.

[60] Dayeh T (2016). DNA methylation of loci within ABCG1 and PHOSPHO1 in blood DNA is associated with future type 2 diabetes risk. Epigenetics.

[61] Schrader S (2022). Novel subgroups of type 2 diabetes display different epigenetic patterns that associate with future diabetic complications. Diabetes Care.

[62] Sanchez-Archidona AR (2021). Plasma triacylglycerols are biomarkers of β-cell function in mice and humans. Mol Metab.

[63] Kupers LK (2022). Maternal dietary glycemic index and glycemic load in pregnancy and offspring cord blood DNA methylation. Diabetes Care.

[64] Lee S (2019). The association of genetically controlled CpG methylation (cg158269415) of protein tyrosine phosphatase, receptor type N2 (PTPRN2) with childhood obesity. Sci Rep.

[65] Sundaram SM (2022). Location matters: hexokinase 1 in glucose metabolism and inflammation. Trends Endocrinol Metab.

[66] Vezza T, Victor VM (2021). The HIF1α-PFKFB3 pathway: a key player in diabetic retinopathy. J Clin Endocrinol Metab.

[67] Li JW (2016). Interactome-transcriptome analysis discovers signatures complementary to GWAS loci of type 2 diabetes. Sci Rep.

[68] Breeze CE (2021). Epigenome-wide association study of kidney function identifies trans-ethnic and ethnic-specific loci. Genome Med.

[69] Barfield RT (2014). Accounting for population stratification in DNA methylation studies. Genet Epidemiol.

[70] Galanter JM (2017). Differential methylation between ethnic sub-groups reflects the effect of genetic ancestry and environmental exposures. Elife.

[71] Schlosser P (2021). Meta-analyses identify DNA methylation associated with kidney function and damage. Nat Commun.

[72] Cai Y (2021). H3K27me3-rich genomic regions can function as silencers to repress gene expression via chromatin interactions. Nat Commun.

[73] Woroniecka KI (2011). Transcriptome analysis of human diabetic kidney disease. Diabetes.

[74] Wilson PC (2019). The single-cell transcriptomic landscape of early human diabetic nephropathy. Proc Natl Acad Sci U S A.

[75] Theilade S (2013). Arterial stiffness is associated with cardiovascular, renal, retinal, and autonomic disease in type 1 diabetes. Diabetes Care.

[76] Chan JC (2014). Premature mortality and comorbidities in young-onset diabetes: a 7-year prospective analysis. Am J Med.

[77] Li H, Durbin R (2009). Fast and accurate short read alignment with Burrows-Wheeler transform. Bioinformatics.

[78] Levey AS (2005). Definition and classification of chronic kidney disease: a position statement from kidney disease: improving global outcomes (KDIGO). Kidney Int.

[79] National Kidney F (2012). KDOQI clinical practice guideline for diabetes and CKD: 2012 update. Am J Kidney Dis.

[80] Saleem MA (2002). A conditionally immortalized human podocyte cell line demonstrating nephrin and podocin expression. J Am Soc Nephrol.

[81] McGrath S (2020). Meta-analysis of the difference of medians. Biom J.

[82] Schroder MS (2011). survcomp: an R/Bioconductor package for performance assessment and comparison of survival models. Bioinformatics.

