# Supplementary material for: Reduced methylation correlates with diabetic nephropathy risk in type 1 diabetes
Source: J Clin Invest. 2023 Feb 15;133(4):e160959. doi: 10.1172/JCI160959 (PMC9927943; doi:10.1172/JCI160959)
Supplement: Supplemental data [file jci-133-160959-s071.pdf]

Supplementary Materials and Data for

Reduced methylation correlates with diabetic nephropathy risk in type 1 diabetes.

Ishant Khurana, Harikrishnan Kaipananickal, Scott Maxwell, Sørine Birkelund, Anna Syreeni, Carol Forsblom, Jun Okabe, Mark Ziemann, Antony Kaspi, Haloom Rafehi, Anne Jørgensen, Keith Al-Hasani, Merlin C. Thomas, Guozhi Jiang, Andrea O.Y. Luk, Heung Man Lee, Yu Huang, Yotsapon Thewjitcharoen, Soontaree Nakasatien, Thep Himathongkam, Christopher Fogarty, Rachel Njeim, Assaad Eid, Tine Willum Hansen, Nete Tofte, Evy C. Ottesen, Ronald C.W Ma, Juliana C.N Chan, Mark E. Cooper, Peter Rossing, Per-Henrik Groop, and Assam El-Osta.

Correspondence to: sam.el-osta@monash.edu

This PDF file includes:

Methods

Figures. S1 to S8

Tables. S1 to S13

Methods

Diabetic nephropathy definition

Individuals with diabetes were categorised as follows; normal AER (normo - AER, <30mg/24h), microalbuminuria (micro - AER ≥30 and <300mg/24h), macroalbuminuria, (macro - AER ≥300mg/24h) and a group with End Stage Renal Disease (ongoing dialysis or kidney transplantation) (1). In addition to AER, urinary albumin-to-creatinine ratios (UACRs) were also available for the discovery and replication cohorts.

Kidney Disease: Improving Global Outcomes (KDIGO) classification

Kidney Disease: Improving Global Outcomes (KDIGO) classification as recommended by American diabetes association (ADA) (**Supplementary Table 9)**.

FinnDiane Study

Individuals with ESRD had a high burden of other diabetic complications such as retinopathy and cardiovascular disease. None of the healthy controls had a family history of diabetes or kidney disease or reported any other serious medical conditions. The protocol for AER measurements and clinical definition has been described previously and (2). Renal function (estimated glomerular filtration rate, eGFR) was calculated using the Chronic Kidney Disease Epidemiology Collaboration (CKD-EPI) formula (3). HbA1c values were obtained at the same time as venous blood and clinical characteristics. Power calculations were performed using modified analysis from previous publications (4-7). We calculated >90% statistical power to detect methylation difference at nominal significance (*P* value $<1\times10{-}6$), with an average reads per sample of 30 million and experimental coefficient of variation 0.12, the minimum detectable methylation difference between samples is 18%.

FinnDiane SNP genotyping

Previously published SNP data (8) of 6019 FinnDiane participants genotyped with the Human Core Exome Bead arrays (versions 12-1.0, 12-1.1 or 24-1.0 - Illumina, San Diego, CA, USA) was used to assess SNP-CpG site associations. In brief, 316,899 genotyped SNPs were used for imputation with 1000 Genomes European Phase 3 version 5 reference panel (Minimac3/Minimac3-omp version 1.0.14) resulting in 8.3 million SNPs (info ≥0.70). DNA methylation of the DDN gene set was assessed for SNPs (**Supplementary Table 12)**. SNP data was available from 265 T1D individuals from the FinnDiane replication cohort, using minor allele frequency of ≥1% at genomic locations of DDN gene set (*MTOR, RPTOR, IRS2, COL1A2, TXNRD1, LCAT* and *SMPD3*).

Replication cohorts

All individuals with diabetes from Hong Kong Diabetes Register attended after an overnight 8-hour fast and underwent structured comprehensive assessments, including eye, feet, urine, and blood examinations. Eye examination included visual acuity and fundoscopy through dilated pupils or retinal photography. A sterile, spot urine sample was used to measure the albumin-to-creatinine ratio (UACR). Microalbuminuria was defined as UACR of 25 to 300mg/g in females and 30 to 300 mg/g in males. Macroalbuminuria was defined as UACR > 300mg/g. eGFR was calculated according to the CKD-EPI equation. Study participants were classified with type 1 diabetes if they presented with diabetic ketoacidosis or required continuous use of insulin within 1 year of diagnosis. Healthy participants were recruited from the community in a community-based health screening program (9). DNA was extracted from peripheral blood leukocytes using standard procedures. Ethical approval was obtained from the Chinese University of Hong Kong Clinical Research Ethics Committee. All study participants gave written informed consent for data analysis and research purpose at the time of recruitment.

For Thai individuals with T1D, demographic data, random plasma C-peptide levels, and blood samples were collected. Individuals with T1D who developed diabetic nephropathy (n=9, micro - UACR ≥30<300mg/g and macro - UACR >300mg/g) were compared with individuals without renal complications (n=56) and age-matched healthy controls (n=65). C-peptide was measured by a chemiluminescent immunometric assay (IMMULITE ®, Siemens) which had inter-assay coefficient of variation 3.3 % at plasma C-peptide 0.6 ng/mL. Diabetic nephropathy staging was classified and aligned using albuminuria range (mg/g) measured by urinary albumin-to-creatinine ratio (UACR) in all samples in the discovery and replication cohorts.

Methylation sequencing (methyl-seq)

Purified gDNA was fragmented by sonication using the BioRuptor (Diagenode); fragmentation was confirmed by capillary electrophoresis on the MultiNA (Shimadzu). 500 ng of fragmented genomic DNA was used for methyl-CpG enrichment using MethylMiner (Life Technologies) according to the manufacturer's instructions (positive control methylated DNA spike-in option used). DNA was eluted from the Methyl-CpG Binding Domain-coupled magnetic beads with 0.6 M NaCl, protocol described in more detail (10). Eluted DNA was quantified, and 5 ng of this methylated DNA was used to generate Illumina sequencing libraries using the NEB-Next DNA Library Preparation Kit (New England Biolabs) following the manufacturer's protocol. Cluster generation was performed on an Illumina cluster station at a concentration of 10pM per library, using version 2 Cluster generation kits (single end sequencing) and the flow cell was processed on a Genome Analyzer IIx (Illumina) with 36 cycles using version 2 SBS kits (Illumina). Base calling was performed by the Illumina RTA software version 1.6. Methyl-seq was also performed for human podocytes maintained in culture using the following conditions; NG (normal glucose control), and HG (15 days high glucose) and the demethylating agent 5-Aza-2′-deoxycytidine NG+5adC (normal glucose including 3-day treatment with 5-aza-2′-deoxycytidine, and HG+5adC (15 days glucose including 3-day treatment with 5-aza-2′-deoxycytidine) (n=3 per group). Enriched methylated DNA libraries were prepared using the NEB-Next DNA Library Preparation Kit (New England Biolabs) following the manufacturer's protocol. Sequencing was conducted on the Illumina HiSeq2500 platform, paired-end 150 bp. The data in this article are deposited in NCBI Gene Expression Omnibus and accessible as GSE77011.

Bioinformatic analysis for methyl-seq

DNA methylation were compared between all pairwise combinations of samples using the MACS peak calling software (version 1.4.0) with a fixed shift size of 75 bp and a significance cut-off of 10E-05 (11, 12). Genomic regions showing different methylation patterns between pairs of samples were merged using Bedtools (13, 14). Duplicate reads which aligned to the same location in each sample were removed from further analysis. The number of reads aligning to each region (peaks) were counted using Bedtools multicov, producing a count matrix (reads per region per sample). For the genomic annotation of regions, a custom python script was used. The human (h19) gene annotation file was obtained from Genecode (release 14) (15) and was used to assign regions to genes based on the nearest genebody overlap.

Differentially Methylated Regions (DMRs) were identified by using edgeR (16) with a Generalized Linear Model(17). Biological variation caused by differences in age and gender were included as model factors. The batch differences were adjusted by including library cluster concentrations as part of the modelling. To account for differences in cell heterogeneity, we utilized reference-based cell-type deconvolution algorithm (in R) (18). Reference-based cell-type deconvolution uses cell-type specific differentially methylated regions (DMRs) to infer cell type proportions, we performed this type of analysis for our data and show the methylation variance associated with cell-type markers for three major white blood cells (B-cell; CD19, T-cell; CD3D, Monocyte; CD14). B cell marker showed the greatest variance among the sequenced samples (**Fig. S1C**), therefore the methylation status of the B cell marker, CD19, was used to adjust heterogeneity. To validate the modelling, the ranked beta values of variables were tested against related gene sets. Statistical tests were based on a 6-way comparison between controls and cases with different renal status. The following contrasts were included; Healthy vs Normo, Healthy vs Macro, Healthy vs ESRD, Normo vs Macro, Normo vs ESRD and Macro vs ESRD. DMR clusters were identified by using supervised heatmap analysis was performed using ComplexHeatmap (19) in R split by k-means clustering (minimum row_km=4).

The data imputed was DMRs identified at *P* value <0.01 for the following comparisons; Healthy vs All cases, Healthy vs Normo, Healthy vs. Macro, and Healthy vs. ESRD.

Next, differentially methylated regions identified by edgeR were integrated with the publicly available databases EpiExplorer (20) and ENCODE genome project (21). EpiExplorer is a web tool for exploring genome and epigenome data on a genomic scale and was used to expand the association of DMRs with regulatory elements such as TSS, gene promoters, exons, introns, and Transcription Factor Binding Sites (TFBSs). All EpiExplorer analyses compared identified DMRs with a randomly generated background set of regions to give insights into enrichment. Regions of reduced methylation localise in or around CpG islands (CGIs) (22). To examine this, DMRs identified were integrated with coordinates (Bedfile) of CpG islands from the human genomes (hg19), downloaded from UCSC Genome Browser. Then, DMRs were integrated with regions around CGIs defined as CGI shores and shelves, which are the regions immediately flanking and up to 5kb away from CpG islands (CpG shores; ± 1kb from CGIs, CpG shelves; ± 1-5kb from CGIs). These regions are interesting because they are variably methylated in cancer and development (23). To generate a list of coordinates for CGI shores and shelves, we used Bedtools to extend genomic regions by 1kb for shores and 1-5kb in either direction from CGIs. Then subtractBed was used to remove CGIs coordinates, and therefore coordinates for only shores and shelves remained. Further analysis on TFBS association with differentially methylated genes was performed by intersecting Encode TFBS data selected from experiments conducted in human tissues including heart, kidney, retina, immune cells and the vasculature.

Pathway analysis for differentially methylated regions annotated to genes (DMGs) was performed using Reactome (24, 25) and Gene set enrichment analysis (GSEA software) utilizing predefined gene sets from the Molecular Signatures Database (MSigDB v5.0) (26). A false discovery rate (FDR) threshold of 0.05 was used for selection of gene sets derived from the Reactome pathway database. Gene network analysis and visualization was performed using enrichment map (27) within Cytoscape software (28).

Human podocytes Methyl-seq data analysis was performed using a similar workflow to the FinnDiane Leukocyte data. Briefly, sequenced reads were aligned to a human reference genome (hg19) using BWA-MEM with default alignment parameters and then the sequence alignments for each read were counted. Profiles of DNA methylation were compared between each sample and its respective input, using the MACS peak calling software. The numbers of reads aligning to each region were extracted, producing a matrix of counts (reads per region per sample). Differentially Methylated Regions (DMRs) were identified by using edgeR with a Generalized Linear Model with FDR threshold of 0.05.

Prospective analysis for progression of diabetic nephropathy

A subset of the participants from the FinnDiane and PROFIL validation cohorts were evaluated prospectively for disease progression by change in albuminuria and also follow-up eGFR (estimated glomerular filtration rate). Samples from the diabetes cohorts were classified as non-progressors and progressors based on change in albuminuria stages (Normo to Micro; Micro to Macro; Macro to ESRD). Decline in eGFR was calculated as follows: (last eGFR – first eGFR)/ (years between measurements). No decline was defined as an eGFR slope of > -1ml/min/1.73 m^2, a slow decline as an eGFR slope of > -3 and < -1 ml/min/1.73 m^2 and steep decline was defined by eGFR slope of < -3ml/min/1.73 m^2. Some samples were excluded from the analysis based on age at diabetes diagnosis (>30 yrs. old; n=68), no follow-up eGFR data (n=6), time between baseline and last eGFR <2 years (n=5), more than 50% eGFR reduction in 2 years (n=1). In total, we collected prospective data from 527 individuals from the FinnDiane (n=180) and PROFIL (n=347) cohorts. The predictive value of the DNA methylation index was also evaluated in the FinnDiane and PROFIL cohorts by Receiver Operating Characteristics (ROC) analysis.

ROC analysis (ROCR package in R) was used to assess the accuracy of the DDN gene set methylation for predicting eGFR decline as a binary outcome (No decline and Steep decline). The AUC (Area Under the Curve) was computed to measure the performance of DNA methylation for the estimation of eGFR decline. Higher the AUC, the better the model is at identifying individuals with future decline in glomerular filtration rate. Clinical covariate modelling performance for ROC analysis was examined using Modern Applied Statistics with S (MASS) package in R. The initial GLM model for eGFR decline prediction included Age, Sex, DM Duration, Baseline HbA1c, Baseline eGFR, Baseline UACR, Systolic Blood Pressure, BMI and Smoking (yes/no). Covariate selection using stepAIC function reported that DM duration, baseline HbA1c, baseline UACR, SBP, and current smoking remained in the GLM model, therefore these clinical indices were assessed for eGFR decline prediction by ROC analysis.

Human cell culture

Human proximal convoluted tubule cells, PCT cells (ATCC, Cat#CRL-2190), were cultured in DMEM/F12 media (GIBCO, Cat# 11330099) containing 10% FBS, 1% Antibiotic-Antimycotic (GIBCO) and 1 % GlutaMAX (GIBCO). We set up a model of macrophage cells, starting from THP-1 monocytes differentiated into macrophages using 30nM Phorbol 12-myristate 13-acetate (PMA, Sigma-Aldrich, Cat# P1585) for 72hours with RPMI1640, 10% FBS and 1% Antibiotic-Antimycotic (GIBCO). Most of the cells were differentiated after 24hour PMA treatment. After 72 hours, the cells were cultured without PMA in RPMI 1640 medium containing (GIBCO, Cat# 11875-093), 2% FBS and 1% Antibiotic-Antimycotic (GIBCO).

Immortalized human vascular endothelial cells (HMEC-1; ATCC, Cat# CRL-3243) were maintained in MCDB-131 medium (GIBCO, Cat # 10372019) supplemented with 10% FBS (GIBCO), 1% Antibiotic-Antimycotic (GIBCO, Cat# 15240062) and 1% GlutaMAX (GIBCO, Cat# 35050061) as previously described (29). Primary non-diabetic and T1D diabetic human aortic endothelial cells (HAECs) were obtained from Lonza (Cat# CC2535; CC2919, n=3 healthy and n=3 T1D donors) and cultured in EBM-2 medium (Lonza) containing EGM-2 growth factors and supplements (Lonza, Cat# 3202) and 10% FBS (GIBCO).

At confluence all cells models were serum starved for 24h and FBS supplementation was reduced to 2% before glucose treatment. The glucose treatment response was performed for 15 days to a medium containing either 30 mM high glucose (HG) or 5.5 mM normal glucose (NG), medium was changed every three days. For PCT glucose treatment was 40 mM high glucose (HG) or 17.5 mM normal glucose (NG). At day 12, cells were cultured for 3 days in HG or NG glucose medium treated with 8µM of the DNA methylation inhibitor 5-aza-2′-deoxycytidine (Sigma, Cat# A3656; HG-5adC/NG-5adC) and DMSO (vehicle control).

Chromatin immunoprecipitation (ChIP)

Human podocytes, HMEC-1 and HAECs post glucose exposure were washed and cross-linked with 1% formaldehyde (in phosphate buffered saline without calcium and magnesium) for 10 min at room temperature. Quenching for excess formaldehyde was performed with 0.125 M glycine for 10 min. Fixed chromatin was suspended in SDS lysis buffer containing 1% SDS, 10 mM EDTA, 50 mM Tris-HCl pH8.1 supplemented with a protease inhibitor cocktail (Roche). Chromatin shearing was performed by sonication (Diagenode bioruptor) for 30 min with 30 sec on and off intervals. Sheared chromatin size (range 250-300 bp) was checked with the MultiNA (Shimadzu).

One quarter of the sheared chromatin was kept as input material. Sheared chromatin was diluted 10 times in ChIP dilution buffer (0.01% SDS, 1% Triton-X, 1.2 mM EDTA, 16.7 mM Tris-HCl pH 8.0, 167 mM NaCl, and protease inhibitor) and incubated overnight at 4°C with ChIP-grade antibodies, including CTCF (Cell Signalling Technology: CST, Cat#2899), Pol2B (Abcam, Cat#AB817) and MeCP2 (Sigma, Cat#9317). An IgG ChIP-grade antibody was used as a negative control for non-specific background enrichment. Antibody-bound chromatin complex was captured with Dynabeads® Protein A magnetic beads (Invitrogen) and 5 washes of each beginning with low salt, high salt, lithium chloride, TE buffer (pH 8.0) followed with TE + 0.01% SDS were performed. Bound DNA was eluted with ChIP elution buffer (20 mM Tris-HCl pH7.5, 5 mM EDTA, 50 mM NaCl, 1% SDS) and reverse cross-linked for 2 h at 62°C on a thermomixer (Eppendorf) set to 1,400 rpm. DNA was recovered and purified with NucleoSpin® PCR Clean-up Columns (Macharey-Nagel) following the manufacturer's instructions. Quantitative PCR was performed and percentage input (% input) was calculated for each ChIP experiment, and results expressed as relative fold enrichment/ratio for the target sequences compared between the case and control groups. Chromatin immunoprecipitation assays were performed five times per sample for CTCF/Pol2B and three times per sample for MeCP2. 10ng of ChIP eluted DNA was retained for ChIP-seq workflow.

Chromatin immunoprecipitation analysis by quantitative real-time (ChIP-qPCR)

Gene-specific chromatin enrichment was assessed using Chromatin immunoprecipitation followed by quantitative PCR (ChIP-qPCR). Target gene threshold cycle numbers (C^t) values were converted to the final relative values of % input enrichment. To compare between ChIP enrichment in case compared to control samples, enrichment was calculated against NG-controls for the respective cell lines. Data are shown as mean ± standard error of the mean (SEM). Statistical significance and *P*-values were calculated by 2-tailed Student's *t*-tests for in-vitro experiments (Graphpad Prism) by comparing groups.

Human podocyte ChIP-seq

Purified ChIP DNA sequences (CTCF, Pol2B and MeCP2 antibodies) and their input controls were sequenced on the Illumina platform (n=2, per ChIP antibody). ChIP-seq libraries were prepared using the NEB Next DNA Library Prep Reagent Set for Illumina (New England Biolabs, Frankfurt/Main, Germany), according to the manufacturer's protocol. Briefly, 10 ng of immunopurified DNA or genomic DNA from an input sample was end-repaired, followed by the 3′ addition of an adenosine nucleotide and ligated to universal library adaptors. Libraries were sequenced at a concentration of 13 pM on the Illumina HiSeq2500 (Illumina, San Diego, CA, USA) with a 150 bp single read length. Sequenced tags were aligned to the human reference genome hg19 using BWA-MEM with default settings. Uniquely mapped reads with no more than one mismatch were used for binding peak detection. Profiles of ChIP-seq peaks were compared between each sample and its respective input, using the MACS peak calling software. The numbers of read tags aligning to each region were extracted, producing a matrix of counts (tags per region per sample). Differentially Methylated Regions (DMRs) were identified by using edgeR with a Generalized Linear Model with FDR threshold of 0.05.

Human podocyte mRNA-seq

One million human podocyte cells (n=3, per group) were used for total RNA extraction using Trizol reagent (Thermo Fisher Scientific, MA), followed by Direct-zol column purification (Zymo Research). RNA quality was verified on the Shimadzu MultiNA capillary electrophoresis system (Shimadzu, Japan). Following Dynabead Oligo(dT) enrichment (Invitrogen), mRNA was prepared into sequence ready libraries with the NEBNext mRNA Library Prep Reagent Set for Illumina (New England Biolabs). Libraries were sequenced as described above with a 150 bp single read length. Sequence tags were aligned to the human reference genome hg19 and Ensembl transcript reference (Homo Sapiens 75) using STAR aligner (30). The numbers of read tags aligning to each region were extracted producing a matrix of counts (tags per gene per sample). Genes with less than 100 sequence reads across all samples were removed from further analysis.

Data integration

We explored the relationship between DNA methylation and transcription factor binding using rank-rank density analysis, comparing clinical samples with human podocytes. We plotted the signed rank of the -log10(p-value) of the differentially methylated peaks detected in FinnDiane vs signed rank of the -log10(p-value) of the same peaks measured for DNA methylation and transcription factor enrichment in human podocytes. This was transformed into a density distribution using a 2-dimensional kernel. This density distribution was then coloured as a heatmap for visualization. The rank-rank analysis was repeated for podocyte data only, comparing DNA methylation and Transcription factor enrichment in human podocytes exposed to chronic HG and 5adC.

Phosphorylation profiling of insulin signalling proteins

Cell lysates isolated from human podocytes exposed to chronic HG and 5adC (n = 2) were lysed using Protein Extraction Buffer (Full Moon BioSystems, Sunnyvale, CA, USA). A total of 2µg of total protein per sample was used to hybridize the PIG219 Ab Array (Full Moon Biosystems) covering major signalling molecules of the insulin signalling and mammalian target of rapamycin (*MTOR*) pathways. Labelling, detection, and array scanning were performed by Crux biolabs (Melbourne, Australia), according to standard protocols. Data were normalized to the average signal intensity on each array and are depicted as fold change between samples.

Infrared protein detection and quantification

Human podocytes cultured in high and normal glucose medium for 15 days (HG/NG), were treated with 8µM of 5-Aza-2′-deoxycytidine (Sigma) and DMSO (vehicle control) at day 12 for 3 days. Protein isolated from podocytes were preserved with Protease Inhibitor Cocktail Mix (Roche) and phosphatase inhibitor cocktail (Thermofisher). Protein was denatured in NuPage® LDS Sample buffer 4X (Invitrogen) at 98°C and 30µg of protein loaded in duplicate (except for the protein dilution series) and run on pre-cast 4–12% Bis-Tris gels (Invitrogen). Gel was transferred by electroblotting to polyvinylidene fluoride (PVDF) membranes overnight. Membranes were incubated with Odyssey blocking buffer (LI-COR Biosciences) prior to incubation with rabbit polyclonal antibodies directed against β-actin (1∶1000, Abcam ab8227), β-tubulin (1∶1000, CST 2128), mTOR (1:1000, CST 2983) and Phospho-mTOR (Ser2448) (1:1000, CST 5536) overnight at 4°C. Donkey anti-rabbit IRDye (680 RD) was applied for 90 minutes at room temperature (1∶5000, LI-COR) prior to washing with PBS (x5). Visualisation and quantification were carried out with the LI-COR Odyssey® scanner and software (LI-COR Biosciences). Western blots experiments were repeated five times per sample.

Core DDN gene detection by methyl-qPCR assay

Methyl-binding-domain capture quantitative PCR (denoted as methyl-qPCR) was used to calculate percentage methylation by comparing amplification of the target sequences (DMRs identified from FinnDiane discovery cohort) in unbound (unmethylated) and bound (methylated) fractions enriched after Methylminer assay. Methylated-control primer was used to amplify the methylated DNA spike-in added to each sample, assisting in normalizing any technical variability between qPCR experiments (housekeeping control). DDN core gene primer sequences are detailed in **Supplementary Table 13**.

Bisulfite sequencing

In total, 100 ng of genomic DNA (five samples of each group) was Bisulfite converted using the EpiTect FAST Bisulfite kit (QIAGEN) following the manufacturer's standard protocol. Bisulfite specific primers were designed so that they contained a high number CpGs within a 500bp amplicon limit, for DMRs detected on genes of interest using MethPrimer(31). PCR was performed in a total volume of 20 µl using Hot start DNA Polymerase (QIAGEN) including 10 pmol each of forward and reverse primers and 4 µl of converted DNA. Reactions were incubated in a thermal cycler under the following conditions: a) 96°C for 15 min, b) 38 cycles of 95°C for 30 sec, 55°C for 1 min and 72°C for 1 min and c) 72°C for 5 min, where Tm°C is the annealing temperature. The amplicons were visualized on a 2% agarose gel under UV light and correct sizes were excised. Purified amplicons were cloned into a pCRII-TOPO vector using TOPO cloning kit (Life technologies) according to the manufacturer's instructions. Plasmids containing the amplicons were sequenced by the Australian Genome Research Facility (AGRF, Melbourne, Australia). Bisulfite sequencing results were analysed by QUMA (32) and DNA methylation level for each region and group was obtained.

Gene expression analysis by quantitative real-time PCR (qPCR)

Total RNA was isolated using Trizol (Invitrogen) and RNEASY kit (QIAGEN; manufacturer's protocol). First-strand cDNA synthesis was performed using a high-capacity cDNA Reverse Transcription Kit (Applied Biosystems) according to the manufacturer's instructions. cDNA primers were designed using oligoperfect designer (Thermo Fisher Scientific). SYBR Green quantitative PCR was performed with the Applied Biosystems 7500 Fast Real-Time PCR System. 5 pmoles of forward and reverse primer, cDNA template and FAST SYBR® Green Master Mix (Roche) were mixed to a final volume of 20 µl. Reactions were incubated at 95°C for 10 min, followed by 40 cycles of 95°C for 10 sec and 60°C for 30 sec. For qPCR data analysis, threshold cycle numbers (C^t) were measured in the exponential phase for all samples and analysed genes were normalized to the level of *H3F3A*. Data are shown as mean ± standard error of the mean (SEM). Statistical significance and *P*-values were calculated by 2-tailed Student's *t*-tests for in-vitro experiments (Graphpad Prism) by comparing groups.

Figure S1. **DNA methylation clustering before and GLM for age, gender and cell heterogeneity**.

(**A**) Principal component analysis (PCA) of unadjusted FinnDiane discovery cohort methylation data. Males (triangle) and females (circle). Each symbol represents DNA methylation profile of each sample categorized by renal complications (ESRD; patients with End stage renal disease n=6, Macro; patients with macroalbuminuria n=9, Normo; patients with no renal complications n=10, healthy controls n=14). (**B**) PCA variances captured by each principal component which highlights majority of the variance occurs between principal component dimensions 1-2. (**C**) Box and whisker (showing the median, min and max values) plot of the DNA methylation variance associated with cell-type markers for three major white blood cells (B-cell; CD19, T-cell; CD3D, Monocyte; CD14) in the FinnDiane discovery cohort. (**D**) Correlation of the top principal components (PC dimension 1-3) with covariates. (**E**) PCA plots of adjusted FinnDiane methylation data clearly shows the importance of using generalised linear modelling (GLM) analyses to adjust age variability, gender and cell heterogeneity.

Supplemental Figure 1

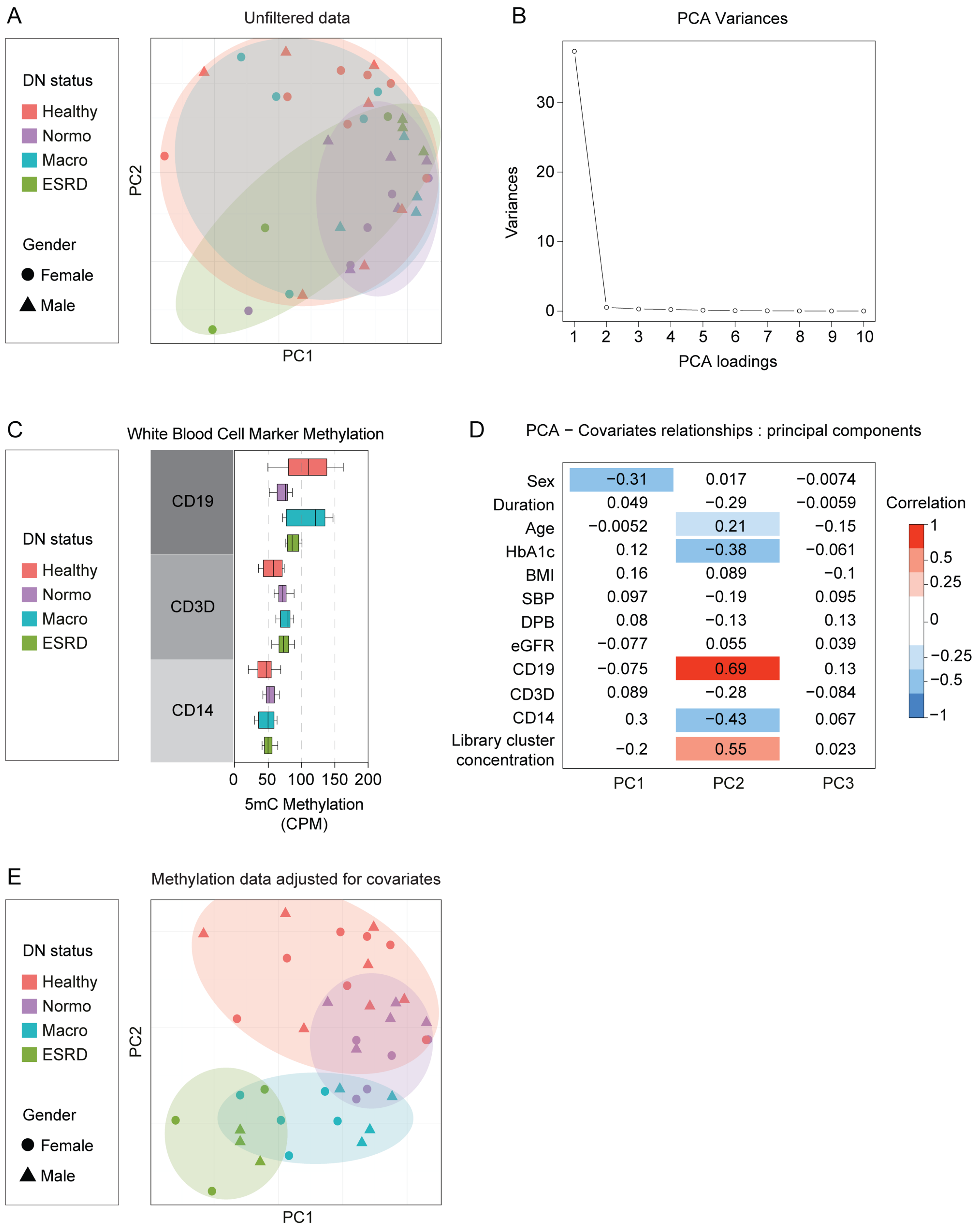

Figure S2. **Differentially methylated regions associated with diabetic nephropathy.**

(**A**) Schematic of the diabetic nephropathy (DN) phenotypic comparisons on the basis of measured Albuminuria (UACR), encompassing different stages and severities of DN; 'Severe DN' comparison targets the DNA methylation affecting the presence of DN, or with severe effects on the phenotype macroalbuminuria compared to T1D with no renal complications (normo). The two ESRD-based case definitions are expected to capture DNA methylation related to the late progression of DN, or end organ injury. Although the 'Diabetes' phenotype may reveal epigenetic factors effected by T1D irrespective of the presence of albuminuria, this comparison also improves the differential analysis because it helps stratify any epigenetic factors related to T1D from those related to DN. (**B**) Multiple scatter plots showing log fold change and abundance of increased and reduced methylated regions are labelled in black text and the percentage of reduced and increased methylated regions labelled in red text. Differentially methylated regions (DMRs) were defined as having a signal in all groups; Healthy vs Normo, Healthy vs Macro, Healthy vs ESRD, Normo vs Macro, Normo vs ESRD and Macro vs ESRD. Top DMRs are shown in red filled circles ($P < 0.01$). A total 10,078 differentially methylated regions passed significance threshold in all groups ($P < 0.01$).

Supplemental Figure 2

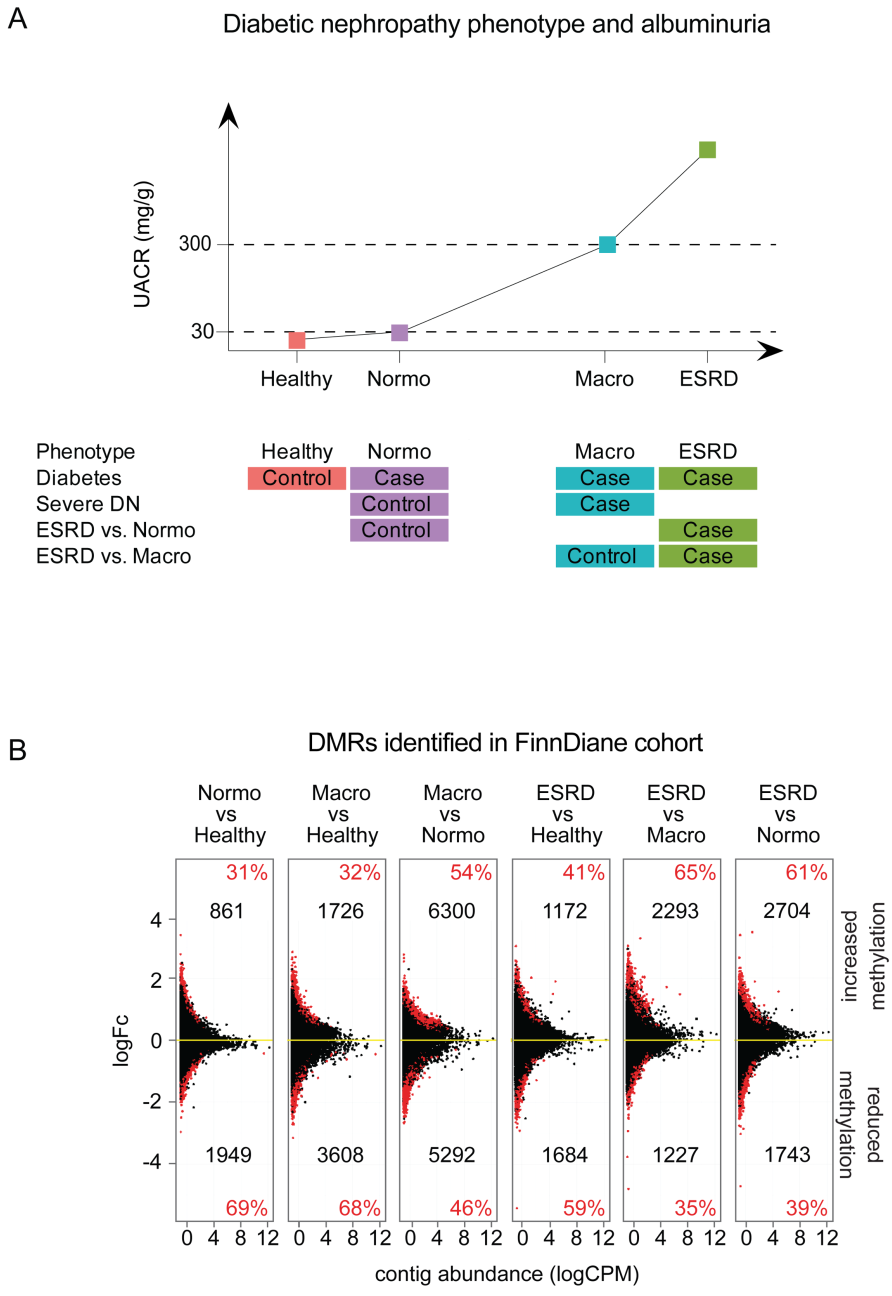

Figure S3. **Functional analysis of FinnDiane methylome**.

(A) Significant differentially methylated pathway networks in the FinnDiane discovery cohort (FDR <0.05). Node size represents individual reactome pathway and gene-set size and colours depicts gain in methylation (red), loss in methylation (green) and connecting common pathways (grey). The interactions (edges) are shown by the curved lines with the arrow showing the direction of the interaction. Many nodes interact and the labels describe the function of the clustered nodes. **(B)** Differentially methylated reactome pathways further categorized by diabetic nephropathy, showing decreased methylation in Normo (light blue), Macro (dark blue) and ESRD (black). The network also shows methylated genes with CTCF binding sites (yellow boxed nodes) in functionally relevant pathways including insulin signalling, lipid metabolism and integrin interactions.

Supplemental Figure 3

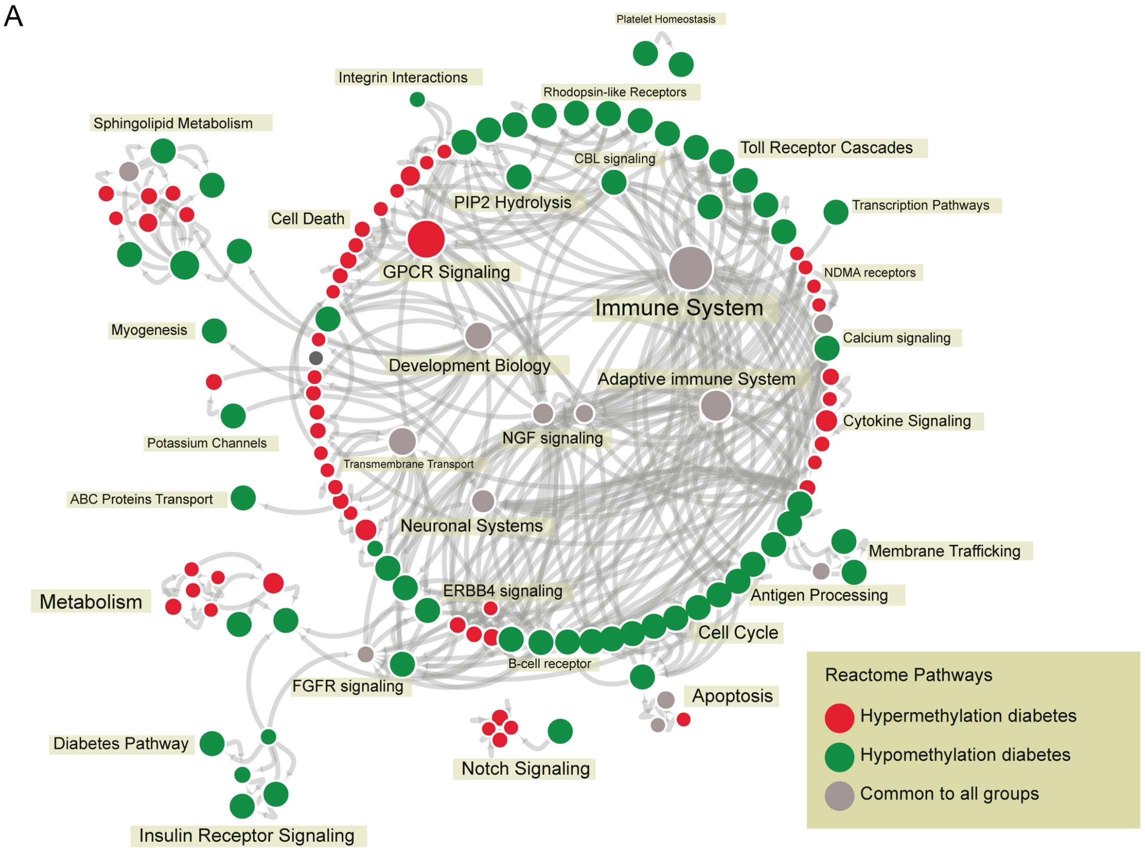

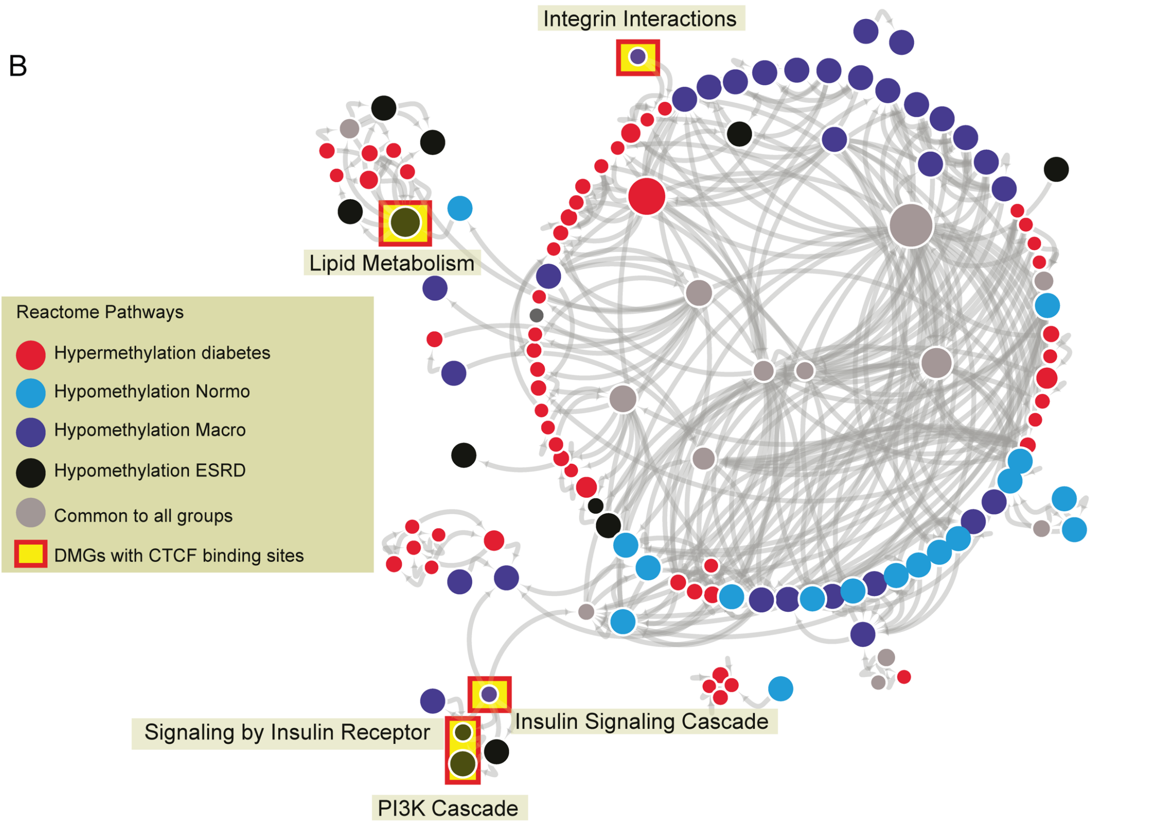

Figure S4. **Key differentially methylated genes associated with progression of diabetic nephropathy in T1D cohorts**.

DNA methylation analysis of the DDN gene set; *MTOR* (chr1:11307862-11309107), *RPTOR* (chr17:78549200-78549666), *IRS2* (chr13:110443347-110443786), *COL1A2* (chr7:94025128-94026206), *TXNRD1* (chr12:104687048-104687537), *LCAT* (chr16:67975360-67975797) and *SMPD3* (chr16:68399936-68400660) were assessed in replication cohorts including **(A)** Finland (n=296), **(B)** Denmark PROFIL, n=445) **(C)** Hong Kong (n=107), **(D)** Thailand (n=130) cohorts using methyl-qPCR. Samples from the replication cohorts were separated into 5 groups, Non-diabetic, and individuals with diabetes were further classified into Low risk, moderate risk, high risk, and very high-risk of developing DN as defined by the KDIGO classification (33, 34). Data show the percentage DNA methylation for individual genes in the different groups presented as box plots and SEM error bars with significance calculated by comparing diabetics with low risk to moderate risk, high risk, and very high-risk using the Mann-Whitney U Test (**P* <0.05, ** *P* <0.01, *** *P* <0.001, **** *P* <0.0001).

Supplemental Figure 4

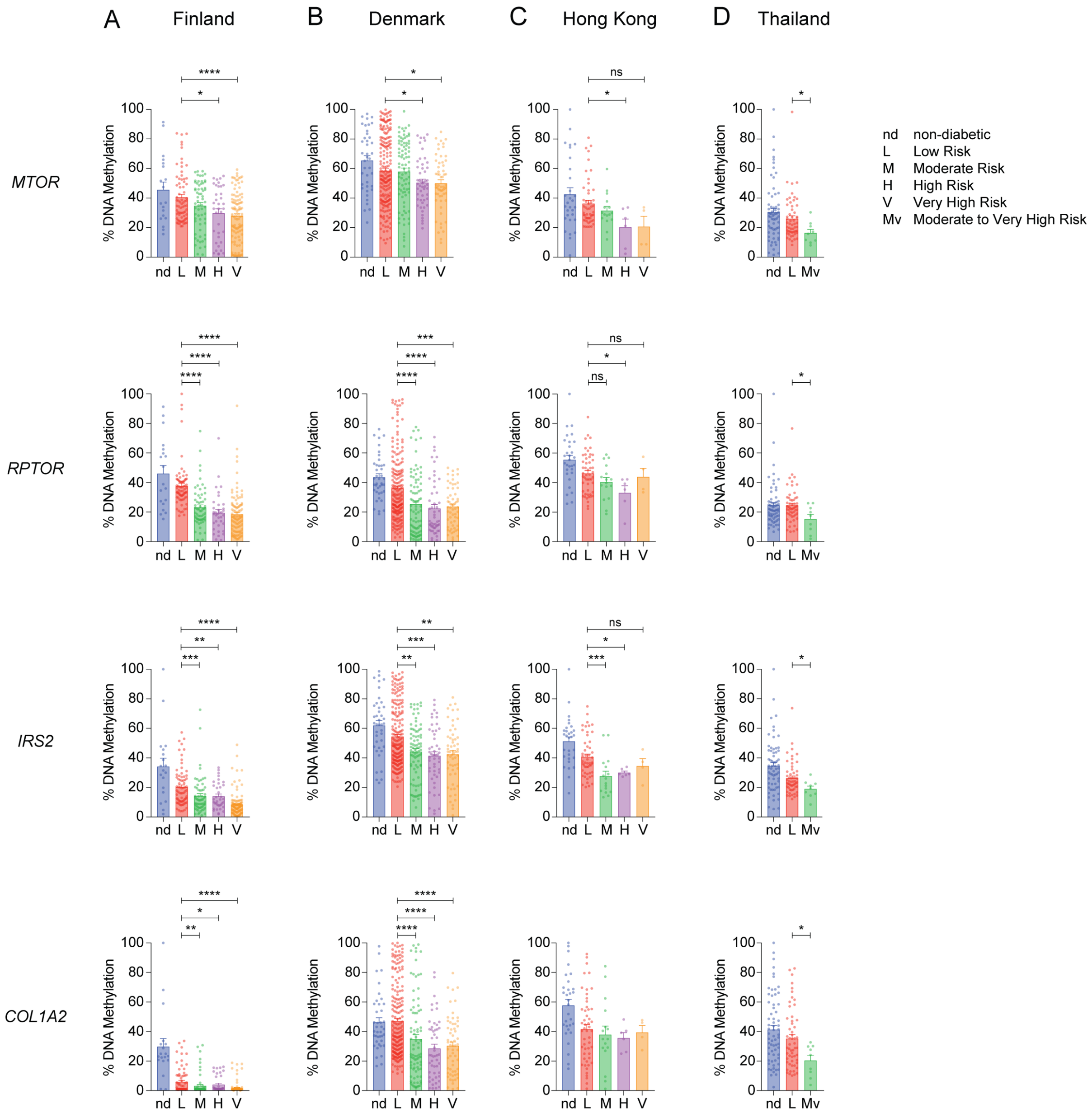

continued over the page

Supplemental Figure 4 continued

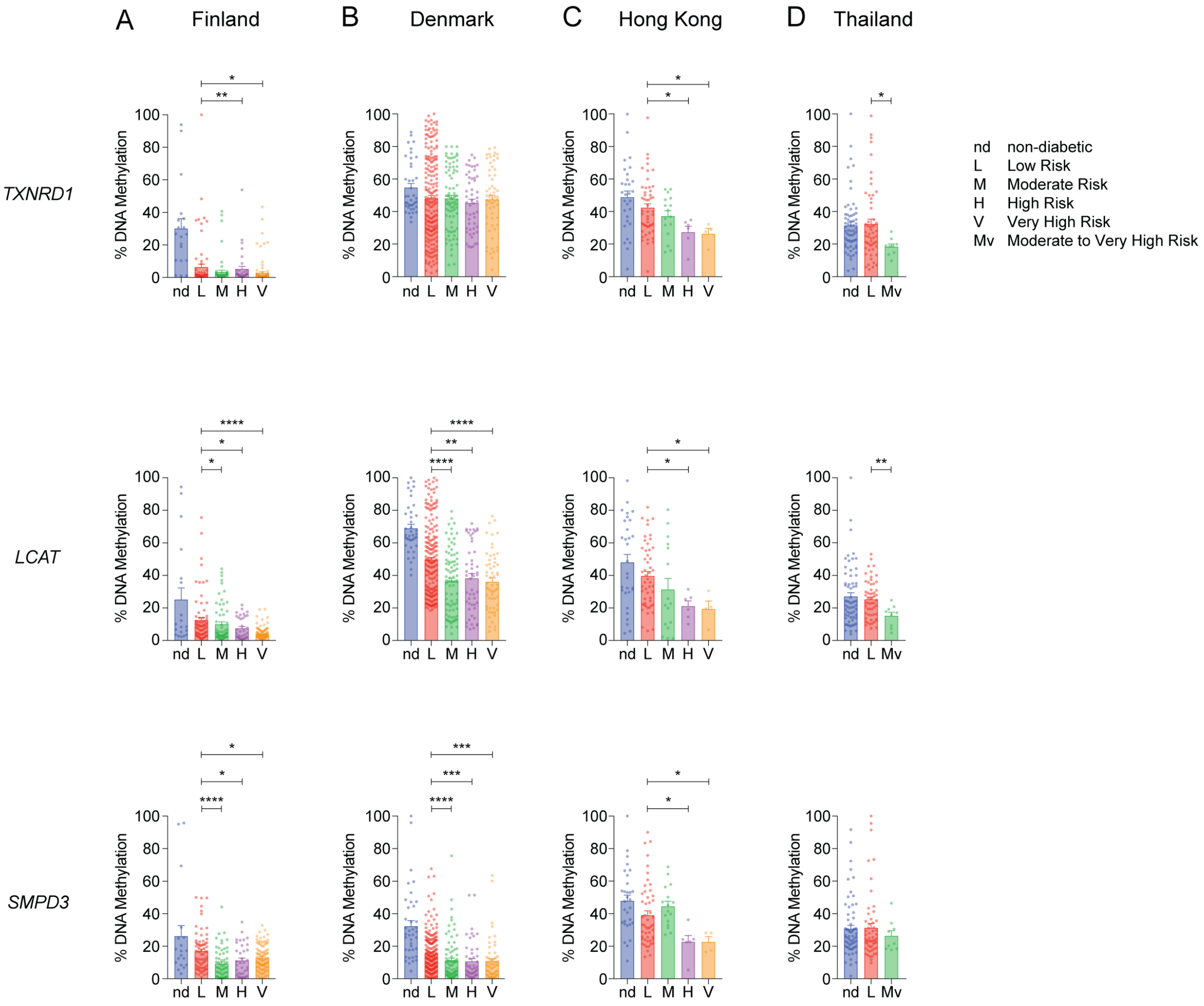

Figure S5. **Previously studied genes associated with progression of diabetic nephropathy in T1D cohorts**.

(A) Data also includes evaluation of methylation control region on *TRAPPC9* (chr8:141286379-141289356) using methyl-qPCR (error bars are SEM) in diabetic cohorts. Data shows no change in DNA methylation in FinnDiane (n=296), PROFIL (n=445), Hong Kong (n=107) and Thailand T1D (n=130). (**B**) Methylation analysis of the Thioredoxin –Interacting Protein gene (*TXNIP*, chr1:145441335-145441734) a glucose sensing gene has been shown to contribute to podocyte injury (35-40), in replication cohorts. Significance calculated by comparing diabetics with low risk to moderate risk, high risk, and very high-risk using the Mann-Whitney U Test (*P <0.05, ** P <0.01, error bars are SEM). **(C)** Methylation analysis of *KCNQ1* (Potassium voltage-gated channel subfamily Q member 1, chr11:2553086-2554119) which is associated with T2D (41), in replication cohorts. Significance calculated by comparing diabetics with low risk to moderate risk, high risk, and very high-risk using the Mann-Whitney U Test (*P <0.05, *** P <0.001, error bars SEM). *TXNIP* and *KCNQ1* could not be assessed in PROFIL cohort due to insufficient amount of methylated DNA.

Supplemental Figure 5

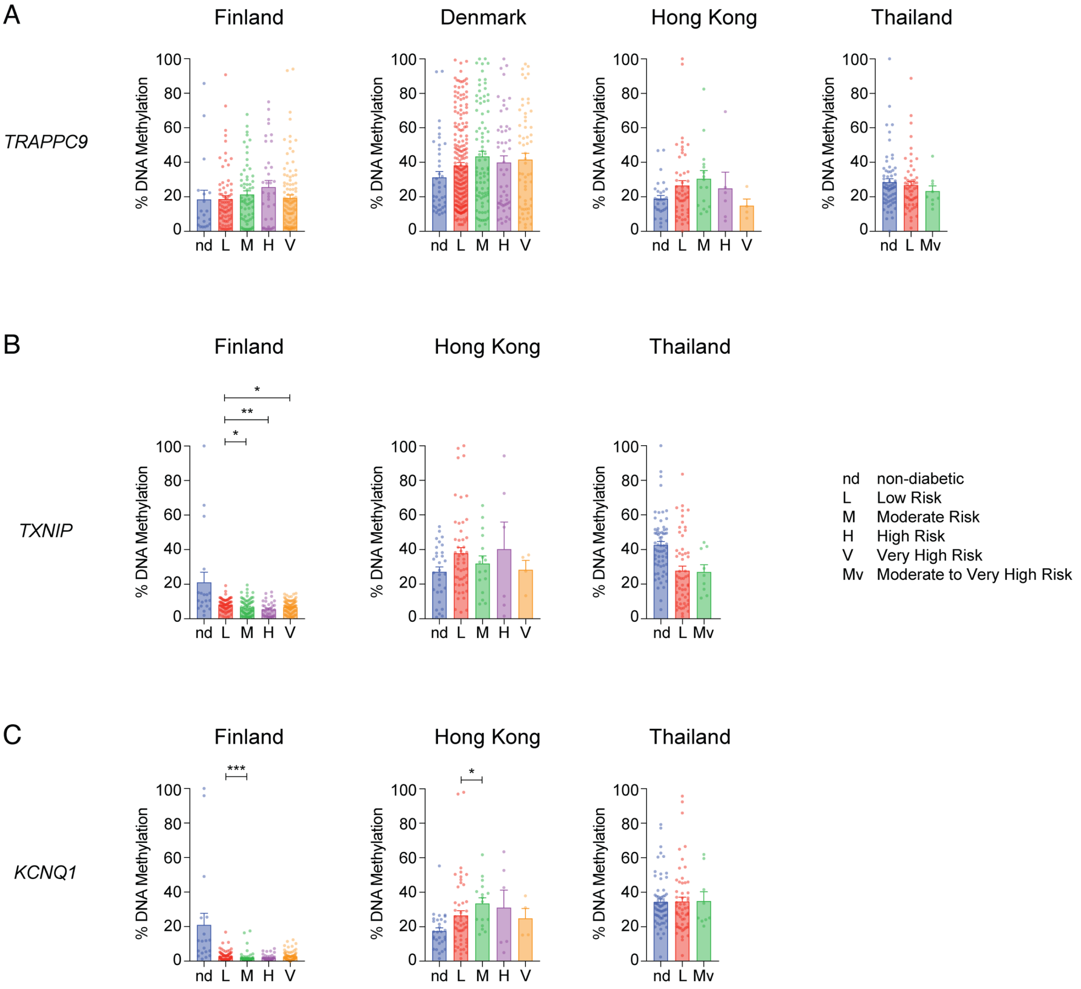

Figure S6: DDN core genes associated with progression of diabetic nephropathy in T1D cohorts based on KDIGO classification versus HbA1c, eGFR and UACR.

Correlation plots of individual DNA methylation profiles of *MTOR*, *RPTOR*, *IRS2*, *COL1A2, TXNRD1*, *LCAT*, and *SMPD3* compared with clinical covariates such as (**A)** HbA1c, **(B)** estimated Glomerular Filtration Rate (eGFR – log2 scale) and **(C)** Urine Albumin-to-Creatinine Ratio (UACR – log2 scale). Regression line are shown for DNA methylation versus HbA1c, eGFR and UACR. Pearson correlation coefficient (*R*) and *P* value (*p*) is reported for each group coloured by KDIGO classification; T1D individuals with low risk (Blue), with moderately increased risk (Purple) and T1D individuals with high (Red) and very high risk of developing ESRD (Green). Covariate regression analysis was conducted for participants from Finland T1D, Denmark T1D, Hong Kong T1D and Thailand T1D cohorts (n=824 - T1D individuals and non-diabetic controls have been omitted).

Supplemental Figure 6

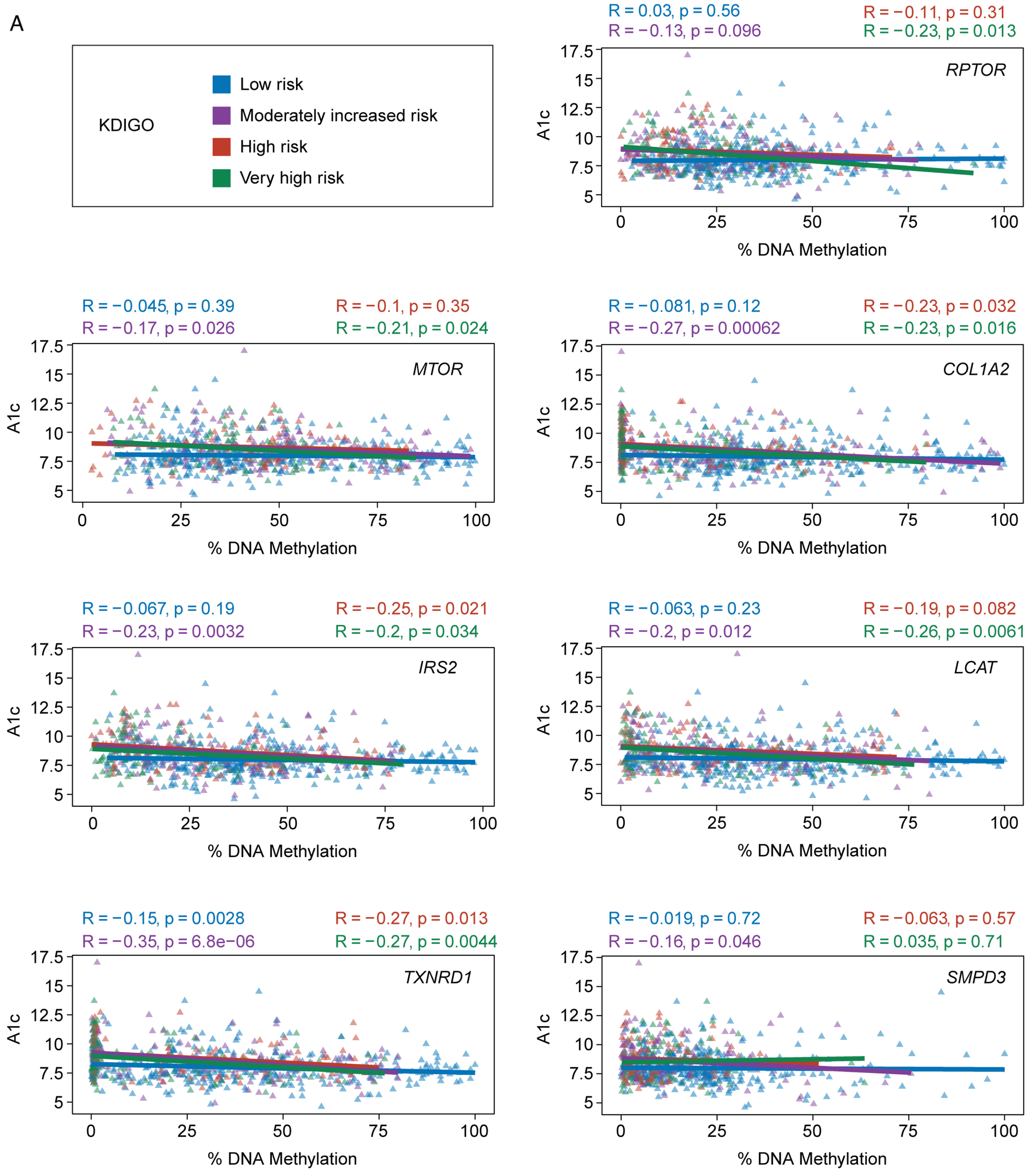

continued over the page

Supplemental Figure 6 continued

B

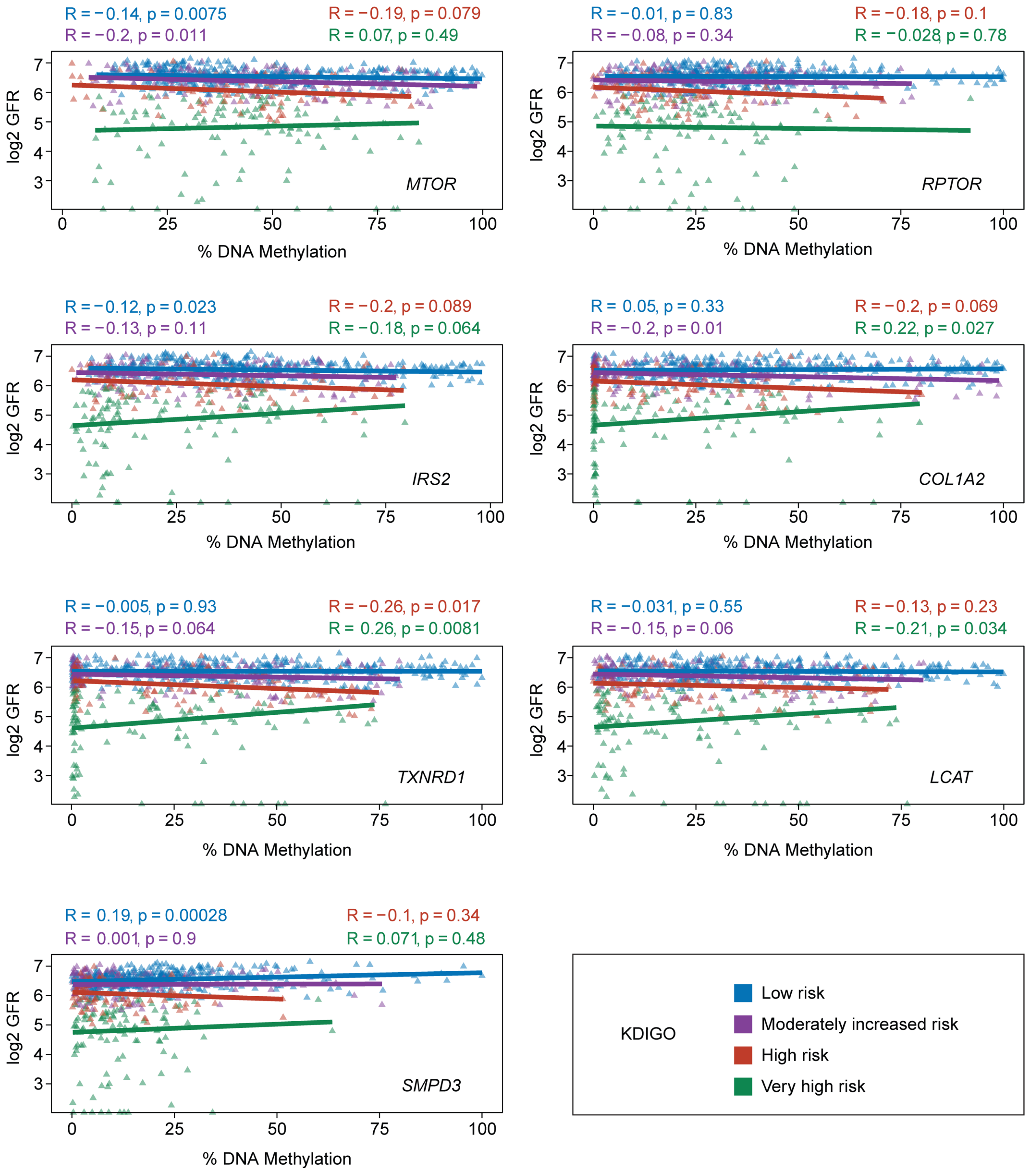

continued over the page

Supplemental Figure 6 continued

C

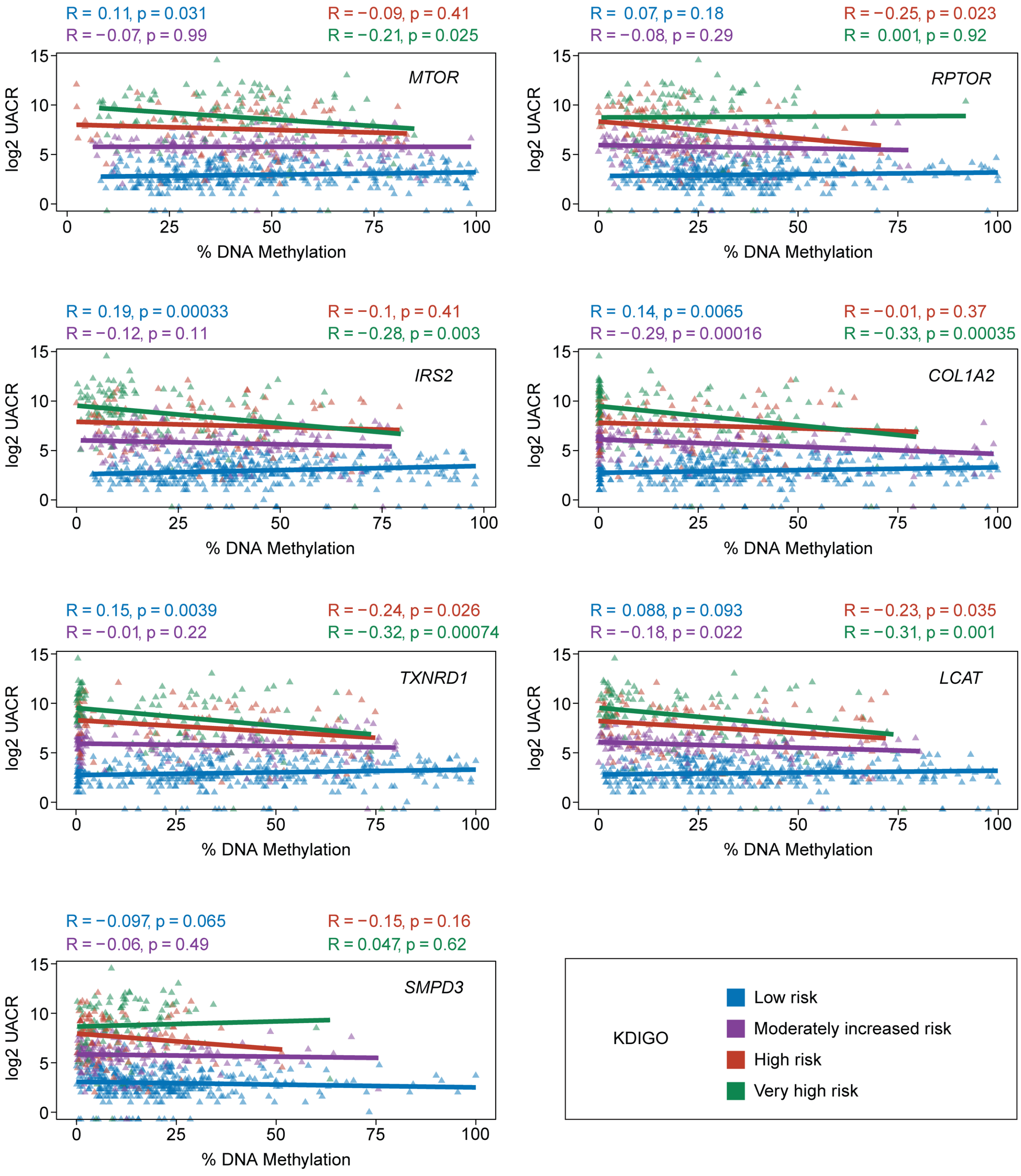

Figure S7. **DNA methylation is inversely correlated with CTCF and Pol2B binding in human podocytes**.

(A) Overview of human podocyte experiments methods, culture conditions, and omics data generated and analysed. Mature human podocytes were cultured in normo-glucose, high-glucose and demethylating agent 5-Aza-2′-deoxycytidine (5adC), more details in supplemental appendix, Experimental Procedures. Human podocyte genomic DNA was assessed for genome-wide DNA methylation using Methyl-seq (n=3). Chromatin immunoprecipitation sequencing (ChIP-seq) in cultured podocytes was preformed using antibodies for CTCF, Pol2B and the DNA methylation reader MeCP2 (n=2). Human podocytes mRNA data was generated using ribosomal-depleted mRNA sequencing (n=3). To complete the multi-omics approach we also performed a Phospho-antibody array for qualitative protein phosphorylation profiling (n=2). **(B)** Comparison of differential methylation and transcription binding in human podocytes. Density plots show regions with gain (+) and loss (-) of DNA methylation (Y-axis) compared to gain (+) and loss (-) binding of MeCP2, Pol2B and CTCF in human podocytes (X-axis). Reduced methylation (in all groups) correlates with increased binding of CTCF and Pol2B (at the same regions) in podocytes after high glucose and/or 5adC exposure, whereas MeCP2 binding is consistent with changes in methylation.

Supplemental Figure 7

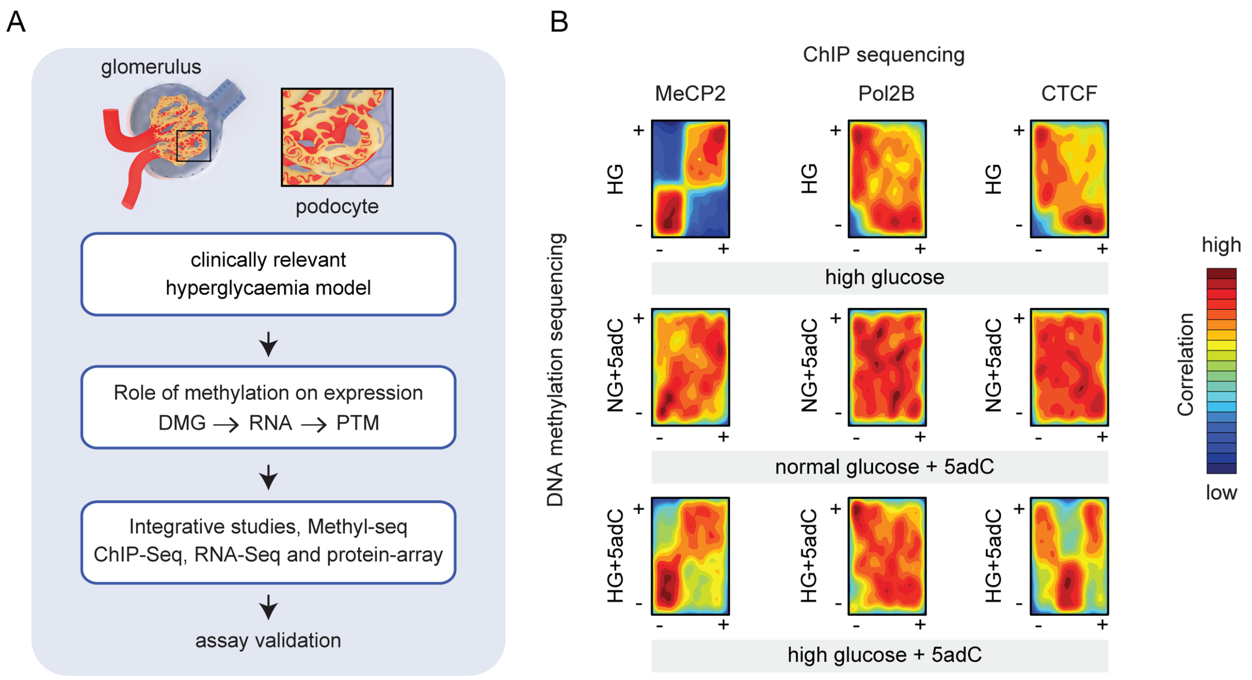

Figure S8. **DNA methylation dependent insulin pathway post-transitional modifications in human podocytes**.

Site-specific protein phosphorylation profiling and screening of insulin pathway showed changes in protein function in human podocytes. Upon increased glucose uptake in podocytes insulin signalling is regulated by DNA independent and dependent protein activation as shown. PTM, post-transitional modification. Digital image adapted from ABCAM.

Supplemental Figure 8

DNA methylation dependent insulin pathway PTM activity in human podocytes

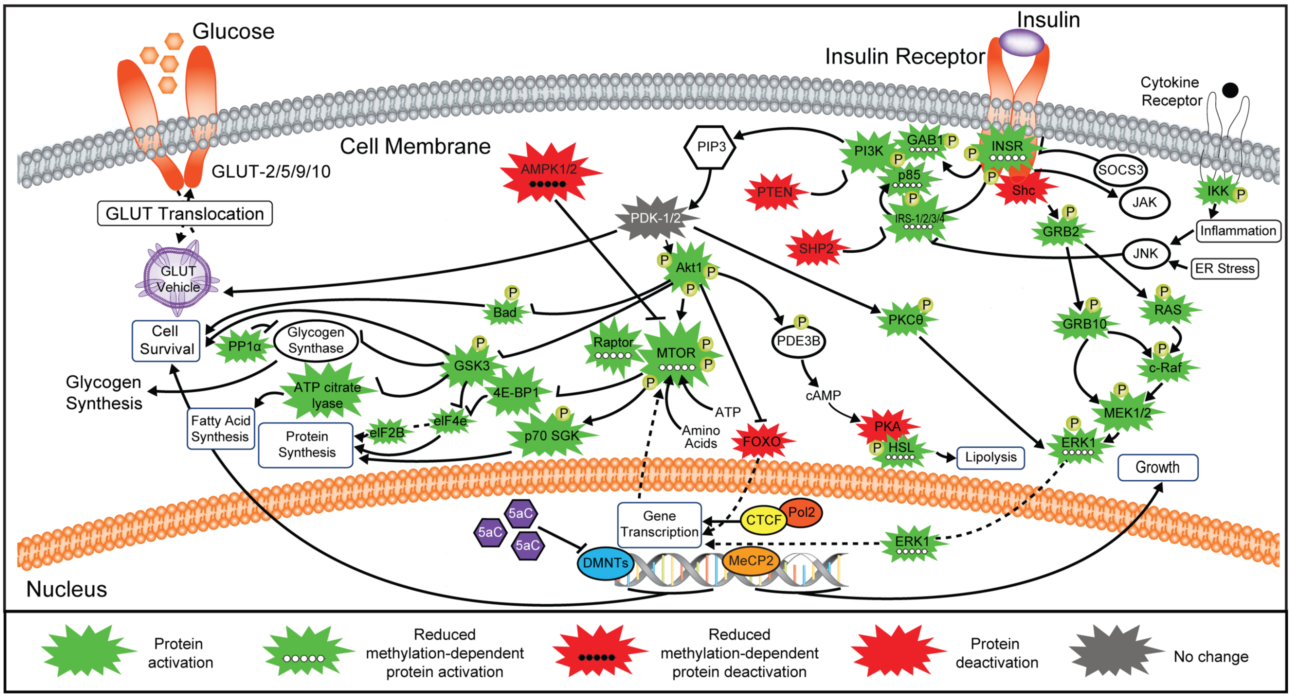

Supplementary Tables

Supplementary Table 1 FinnDiane diabetic nephropathy study - Information on the Finland Discovery cohort

			**Type 1 Diabetes**					
	**Healthy subjects**		**Normoalbuminuria**		**Macroalbuminuria**		**ESRD**	
Sex	Male	Female	Male	Female	Male	Female	Male	Female
Number of individuals	7	7	5	5	4	5	3	3
Age (yrs)	41±7	39±9	46±13	38±8	41±14	40±4	50±5	48±8
Duration of diabetes (yrs)	na	na	24.0±15	27.0±12.8	33.1±16	31.0±14.9	26.5±0.7	41.2±5.5
Age at diabetes onset (yrs)	na	na	22.4±11.3	11.1±7.7	8.0±3.8	9.2±1.9	23.5±4.8	7.0±3.2
Other complications								
- Retinopathy	0	0	2	1	2	2	2	3
- Cardiovascular disease	0	0	0	0	0	0	0	0
Hemoglobin A1c (%)*	5.3±0.3	5.2±0.3	8.1±0.3	8.3±1.5	8.2±1.2	7.7±0.6	8.2±1.0	8.2±0.4
eGFR (ml/min/1.73m^2)**	103.6±12.1	95.9±17.9	100.2±21.6	104±10.7	63.0±39.9	55.2±37.0	27.1±33.0	39.0±26.0
Urinary AER (mg/24h)***	<30	<30	<30	<30	≥300	≥300	na	na

*HbA1c (at sample collection - mean for the group)
**eGFR=estimated glomerular filtration rate calculated with the CKD-EPI formula
***Urinary AER clinical range, healthy and normo <30mg/24h, macro ≥300mg/24h and ESRD >300mg/24h and dialysis
Data are reported as mean ± Standard deviation or median (interquartile range)

Supplementary Table 2 Data quality and methyl-seq metrics

	Healthy subjects		Type 1 Diabetes					
			Normoalbuminuria		Macroalbuminuria		ESRD	
Sex	Male	Female	Male	Female	Male	Female	Male	Female
Number of individuals	7	7	5	5	4	5	3	3
Mean Read Count (M)	30.04	29.11	29.71	31.40	27.10	27.95	31.33	25.95
Mean Mapped Reads (M)	27.27	27.31	28.65	30.40	26.22	26.72	30.04	24.99
Mean Mapped Q20 Count (M)	18.43	18.49	21.02	22.91	19.29	18.21	20.25	17.78
Whole genome coverage^	59%	59%	79%	82%	77%	64%	73%	72%
CpG coverage*	9,028,484	9,986,785	9,754,130	9,696,875	9,129,593	10,533,971	11,274,882	9,530,343
CpG count[#]	4.04	4.01	2.82	2.60	2.83	4.38	3.57	3.27
%GC	47%	46%	44%	44%	44%	48%	45%	46%
%CpG	2%	2%	1%	1%	1%	2%	1%	1%

(M) Million
^12,382,790 segments 250bp bins
* 28 Million CpG sites in human genome
# per 250bp

Supplementary Table 3 DMRs associate with diabetic nephropathy

Cluster	Methylation	DMRs[1]	%DMGs[2]	Genes[3]	%DMGs[4]	Reactome pathways enriched[5]
1	Reduced in ESRD	1697	16.8	1305	76.9	12
2	Reduced in Macro	3227	32.0	2427	75.2	32
3	Reduced in Normo	1792	17.8	1459	81.4	17
4	Increased all cases	3362	33.3	2718	80.8	64

DMRs (differential methylated regions)[1],
% DMRs per cluster[2]
Genes annotated within 20kb[3],
% Differential methylated genes(DMGs)[4]
Number of enriched reactome pathways identified at FDR <0.05 using GSEA[5]

Supplementary Table 4 DMRs associated with type 1 Diabetic Nephropathy

DMR Location	Region length (bp)	Gene	Ensembl ID	P value	EPIC CG site Location*	CG Name
chr4:144404420-144404861	441	GAB1	ENST00000262995	1.69E-04	chr4:144404543-144404545	cg01883784
chr13:110443347-110443786	439	IRS2	ENST00000375856	1.58E-03	chr13:110443739-110443741	cg14196263
chr5:142078293-142078636	343	FGF1	ENST00000494579	6.39E-03		
chr5:170909699-170911557	1858	FGF18	ENST00000274625	6.63E-03	chr5:170909866-170911103	cg13997576, cg09009881, cg26841049
chr17:78549200-78549666	466	RPTOR	ENST00000537330	7.71E-03	chr17:78549279-78549372	cg01991785, cg25514328, cg11329058, cg18576374
chr1:11307862-11309107	1245	MTOR	ENST00000361445	8.79E-03	chr1:11308251-11308253	cg02717678
chr10:22874619-22875118	499	PIP4K2A	ENST00000376573	9.05E-05		
chr12:124864792-124865295	503	NCOR2	ENST00000405201	2.82E-04	chr12:124864912-124865131	cg07954091, cg00765705
chr16:68399936-68400660	724	SMPD3	ENST00000219334	5.86E-04		
chr8:41470262-41470706	444	GPAT4	ENST00000396987	4.00E-03		
chr22:20918886-20919334	448	MED15	ENST00000425759	4.69E-03		
chr6:110104322-110104932	610	FIG4	ENST00000441478	5.14E-03	chr6:110104632-110104634	cg16019558
chr16:67975360-67975797	437	LCAT	ENST00000264005	5.37E-03		
chr12:116716013-116716425	412	MED13L	ENST00000281928	5.99E-03	chr12:116716082-116716359	cg21219443, cg12113819, cg08311322, cg18378267
chr12:104687048-104687537	489	TXNRD1	ENST00000525566	6.44E-03		
chr3:127416330-127417102	772	MGLL	ENST00000434178	7.76E-03	chr3:127416579-127416581	cg13050716
chr1:186955914-186956703	789	PLA2G4A	ENST00000367466	7.77E-03		
chr12:110033681-110035595	1914	MVK	ENST00000539696	8.82E-03	chr12:110034951-110034953	cg12495807
chr3:32185089-32185514	425	GPD1L	ENST00000282541	9.67E-03		
chr7:94025128-94026206	1078	COL1A2	ENST00000297268	1.34E-04		
chr15:68608377-68609157	780	ITGA11	ENST00000315757	5.44E-03	chr15:68608582-68608584	cg01148276
chr11:118069845-118070106	261	JAML	ENST00000356289	6.90E-03		
chr2:227972698-227973120	422	COL4A4	ENST00000396625	8.02E-03		

* DMRs detected by methyl-seq overlapping Infinium MethylationEPIC BeadChip Array Probes

Supplementary Table 5 FinnDiane diabetic nephropathy study - Information on the Finland Replication Cohort

	Healthy subjects		Type 1 Diabetes							
			Normoalbuminuria		Microalbuminuria		Macroalbuminuria		ESRD	
Sex	Male	Female	Male	Female	Male	Female	Male	Female	Male	Female
Number of individuals	7	12	31	34	36	37	35	31	40	33
Age years	45±6.2	43.5±10	38±10	37±11	38±12	38±13	44±11	36±7	46±8	43±9
Duration of diabetes (yrs)	na	na	26.5±10.4	24.6±9.6	26.1±10	23±10.1	27.3±7.6	25.2±6.7	32.2±8.2	30.4±8.1
Age at diabetes onset (yrs)	na	na	12.7±6.4	12.5±5.3	11.6±7.2	15±8.9	15.6±9	10.5±5.7	14.6±8.6	12.3±7.3
Other complications										
- Retinopathy	0	0	13	9	17	19	27	26	37	31
- Cardiovascular disease	0	0	3	0	1	1	5	1	19	12
Hemoglobin A1c (%)*	5.7±0.3	5.6±0.2	8.5±1.2	8.4±1.3	9.5±2.1	9.1±1.4	9.2±1.7	9.7±1.5	8.9±1.6	8.1±1.6
eGFR (ml/min/1.73m^2)**	105±7.7	97.8±11.7	95.1±15.3	88.9±17.9	91.2±18.1	78.6±25.1	50.7±28.3	56.5±27.2	<20	<20
Urinary ACR (mg/g)***	5.4 (2.0-3.3)	13.2 (8.1-13.9)	4.8 (3.7-7.8)	6.3 (4.4-8.3)	73.4 (41.6-97.4)	79.9 (41.0-113)	624 (404-1350)	663 (289-1460)	1763 (913-3414)	1627 (697-2194)
Urinary AER (mg/24h)	<30	<30	<30	<30	≥30,<300	≥30,<300	≥300	≥300	>300	>300

*HbA1c (at sample collection - mean for the group)
**eGFR=estimated glomerular filtration rate calculated with the CKD-EPI formula
***Urinary ACR clinical range, healthy and normo <30mg/g, micro ≥30<300mg/g, macro≥300mg/g and ESRD >300mg/g and dialysis
Data are reported as mean ± Standard deviation or median (interquartile range)

Supplementary Table 6 PROFIL Study - Steno Diabetes Center Copenhagen (SDCC), Denmark

	Healthy subjects		Type 1 Diabetes					
			Normoalbuminuria		Microalbuminuria		Macroalbuminuria	
Sex	Male	Female	Male	Female	Male	Female	Male	Female
Number of individuals	20	20	88	82	67	43	69	56
Age (yrs)	49±14	47±13	55±13	57±10	60±11	59±11	57±10	51±9
Duration of diabetes (yrs)	na	na	38.7±9.4	39.4±9.9	39.2±11.1	44.3±11	38.9±11.5	40.8±7.8
Age at diabetes onset (yrs)	na	na	18.7±9.8	18.0±8.7	21.2±12.1	14.8±9.1	17.9±12.7	10.6±6.0
Other complications								
- Retinopathy	0	0	79	60	61	40	66	55
- Neuropathy	0	0	2	0	0	1	0	1
- Cardiovascular disease	2	0	12	10	22	17	26	12
Hemoglobin A1c (%)*	5.4±0.2	5.4±0.3	7.8±1.0	7.6±0.8	7.9±1.0	8.1±1.0	8.4±1.2	8.2±1.3
eGFR (ml/min/1.73m^2)**	94.0±18.4	94.0±8.0	92.5±15.3	82.7±17.6	81.8±23.0	71.3±23.8	61.6±28.1	60.5±27.1
Urinary ACR (mg/g)***	<30	<30	<30	<30	>30<300	>30<300	>300	>300

*HbA1c (at sample collection - mean for the group)
**eGFR=estimated glomerular filtration rate calculated with the CKD-EPI formula
***Urinary ACR clinical range, healthy and normo <30mg/g, micro ≥30<300mg/g, and macro ≥300mg/g
Data are reported as mean ± Standard deviation or median (interquartile range)

Supplementary Table 7 Hong Kong T1D Registry

		Type 1 Diabetes		
	Healthy subjects	Normoalbuminuria	Microalbuminuria	Macroalbuminuria
Number of individuals	30	39	30	8
Age years	45.8 ± 6.0	37.7 ± 17	37.1 ± 17.3	44.4 ± 23.3
Duration of diabetes (yrs)	na	8 (1-12.5)	10 (2-15.5)	13.5 (10.8-17.5)
Age at diabetes onset (yrs)	na	29.4 ± 19.1	25.9 ± 16.7	29.9 ± 18
Other complications				
- Retinopathy	0	11	4	3
- Neuropathy	0	7	5	2
- Cardiovascular disease	0	2	2	2
Hemoglobin A1c (%)*	na	7.8 ± 1.8	9 ± 2.5	8.5 ± 1.2
eGFR (ml/min/1.73m^2)**	na	107.7 (93.3-120.4)	96.1 (82.1-115.6)	90.8 (62.8-110.7)
Urinary ACR (mg/g)***	5.84±3.4	7.1±4.4	68±31.4	924.8±566.5

*HbA1c (at sample collection - mean for the group)
**eGFR=estimated glomerular filtration rate calculated with the CKD-EPI formula
***Urinary ACR unit mg/g - clinical range, healthy and normo <30mg/g, micro ≥30<300mg/g, and macro ≥300mg/g
Data are reported as mean ± Standard deviation or median (interquartile range)

Supplementary Table 8 Thailand T1D Registry

			Type 1 Diabetes					
	Healthy subjects		Normoalbuminuria		Microalbuminuria		Macroalbuminuria	
Sex	Male	Female	Male	Female	Male	Female	Male	Female
Number of individuals	29	36	23	33	5	2	1	1
Age (yrs)	42±8	38±11	43±7	38±11	42±15	44±16	65	73
Duration of diabetes (yrs)	na	na	22.3±12.3	15.8±9.3	24.8±8.6	31.5±7.8	24	35
Age at diabetes onset (yrs)	na	na	21.4±9.8	22.6±10.9	13±3.4	22.5±6.4	41	38
Other complications								
- Retinopathy	0	0	4	5	3	2	1	1
- Neuropathy	0	0	2	0	0	1	0	1
- Cardiovascular disease	0	0	0	0	0	0	0	0
Hemoglobin A1c (%)*	5.3±0.3	5.2±0.3	7.6±0.3	7.7±1.1	8.2±1.2	7.2±2.6	9	10.9
eGFR (ml/min/1.73m^2)**	102.2±19.2	112.9±22.5	100.7±17.6	113.5±15.2	100.2±12.4	109.0±11.3	45	43
Urinary ACR (mg/g)***	<30	<30	<30	<30	>30<300	>30<300	>300	>300

*HbA1c (at sample collection - mean for the group)
**eGFR=estimated glomerular filtration rate calculated with the CKD-EPI formula
***Urinary ACR clinical range, healthy and normo <30mg/g, micro ≥30<300mg/g, and macro ≥300mg/g
Data are reported as mean ± Standard deviation or median (interquartile range)

Supplementary Table 9 Human Chronic Kidney Disease Classification and Staging

CKD Classification and Staging

- **Low risk (LR)** (red)
- **Moderate risk (MR)** (green)
- **High risk (HR)** (purple)
- **Very high risk (VHR)** (orange)

Kidney function stage eGFR (ml/min/1.73m^2) Description and range				**Kidney damage stage** Urine albumin/creatine ratio (UACR) Description and range		
				A1	A2	A3
				Normal to mild increase <30mg/g	Moderate increase 30-300mg/g	Severe increase >300mg/g
	**G1**	Normal or High	≥ 90	**LR**	**MR**	**HR**
	**G2**	Mild decrease	60-89	**LR**	**MR**	**HR**
	**G3a**	Mild to moderate decrease	45-59	**MR**	**HR**	**VHR**
	**G3b**	Moderate to severe decrease	30-44	**HR**	**VHR**	**VHR**
	**G4**	Severe decrease	15-29	**VHR**	**VHR**	**VHR**
	**G5**	Kidney failure	<15	**VHR**	**VHR**	**VHR**

Table adapted from Levey *et al.* Kidney Int. 2011 Jul;80(1):17-28

Supplementary Table 10 Meta-anlaysis of DDN core genes in T1D registries

Gene; DMR location (hg19)	Replication cohorts (n=978)	Low risk vs. Moderately risk: DNAm difference*	Low risk vs. Moderately risk: P value**	Low risk vs. High risk: DNAm difference*	Low risk vs. High risk: P value**	Low risk vs. Very high risk: DNAm difference*	Low risk vs. Very high risk: P value**
**Meta-analysis of DDN genes in FinnDiane, PROFIL, HKT1D and Theptarin T1D cohorts** — Diabetic nephropathy groups							
***MTOR;* DMR in chr1:11,307,861–11,309,108**	FinnDiane	-3.3250	3.00E-01	-9.6940	3.91E-02	-11.1200	1.00E-04
	PROFIL	-0.4713	8.91E-01	-7.665	2.62E-02	-7.92	2.41E-02
	HKT1D	-1.061	5.87E-01	-13.44	4.72E-02	-15.67	1.55E-01
	ThT1D	-7.296	1.71E-02	-7.296	1.71E-02	-7.296	1.71E-02
	Combined #	***-1.06***	***1.58E-01***	***-7.67***	***4.72E-04***	***-7.92***	***8.39E-06***
***RPTOR;* DMR in chr17:78,549,199–78,549,667**	FinnDiane	-14.4200	1.00E-04	-17.69	1.00E-04	-18.97	1.00E-04
	PROFIL	-10.26	1.00E-04	-12.48	1.00E-04	-9.042	3.00E-04
	HKT1D	-4.715	1.25E-02	-11.91	3.51E-02	-0.9262	1.20E-01
	ThT1D	-7.609	3.78E-02	-7.609	3.78E-02	-7.609	3.78E-02
	Combined #	***-10.26***	***1.57E-08***	***-12.48***	***3.92E-08***	***-9.04***	***3.05E-07***
***IRS2*; DMR in chr13:110,443,346–110,443,787**	FinnDiane	-5.43	5.00E-04	-4.665	9.80E-03	-9.848	1.00E-04
	PROFIL	-8.396	3.30E-03	-10.86	5.00E-04	-9.947	1.30E-03
	HKT1D	0.0006	6.00E-04	-7.675	3.11E-02	-4.193	5.01E-01
	ThT1D	-5.378	3.97E-02	-5.378	3.97E-02	-5.378	3.97E-02
	Combined #	**-5.43**	**1.02E-07**	**-7.68**	**8.04E-06**	**-9.85**	**3.89E-06**
***COL1A2;* DMR in chr7:94,025,127–94,026,207**	FinnDiane	-0.2837	2.60E-03	-0.2115	4.93E-02	-0.501	1.00E-04
	PROFIL	-13.1	1.00E-04	-16.72	1.00E-04	-14.6	1.00E-04
	HKT1D	-3.695	5.38E-01	-4.728	5.99E-01	-0.2256	9.87E-01
	ThT1D	-11.47	4.59E-02	-11.47	4.59E-02	-11.47	4.59E-02
	Combined #	***-11.47***	***8.45E-06***	***-11.47***	***1.09E-04***	***-11.47***	***8.67E-07***
***TXNRD1;* DMR in chr12:104687047–104687538**	FinnDiane	0.06569	6.17E-01	0.5531	9.20E-03	-0.2241	3.60E-02
	PROFIL	1.027	7.39E-01	-1.693	6.43E-01	0.7286	8.44E-01
	HKT1D	-3.636	4.29E-01	-13.9	3.93E-02	-14.93	3.96E-02
	ThT1D	-7.879	2.79E-02	-7.879	2.79E-02	-7.879	2.79E-02
	Combined #	***0.07***	***2.36E-01***	***-1.69***	***2.39E-03***	***-0.22***	***8.27E-03***
***LCAT;* DMR in chr16:67,975,360–67,975,798**	FinnDiane	-1.988	3.91E-02	-2.286	4.65E-02	-4.254	1.00E-04
	PROFIL	-12.08	1.00E-04	-11.21	2.90E-03	-11.85	1.00E-04
	HKT1D	-10.98	1.45E-01	-16.42	1.85E-02	-17.95	2.71E-02
	ThT1D	-9.039	7.90E-03	-9.039	7.90E-03	-9.039	7.90E-03
	Combined #	***-10.98***	***6.21E-06***	***-11.21***	***2.18E-05***	***-11.85***	***7.75E-09***
***SMPD3;* DMR in chr16: 68,399,935– 68,400,661**	FinnDiane	-6.524	2.00E-04	-4.939	1.14E-02	-2.788	4.84E-02
	PROFIL	-4.616	1.00E-04	-4.785	2.00E-04	-4.748	1.00E-04
	HKT1D	8.016	9.53E-02	-12.02	2.93E-02	-11.74	4.73E-02
	ThT1D	-1.143	7.16E-01	-1.143	7.16E-01	-1.143	7.16E-01
	Combined #	***-4.62***	***2.25E-06***	***-4.79***	***4.58E-05***	***-4.75***	***1.27E-04***

*% DNA Methylation Difference: Hodges-Lehmann method

***P* Value using Mann–Whitney U test

#Weighted Meta analysis using R packages Metamedian (version 0.1.6) -
- pool.med and survcomp (version 1.22.0)
- combine.test for Fisher's combined probability test

Supplementary Table 11 Insulin pathway protein activation in human podocytes after high glucose and 5adC

Pathway	Protein	Detection Ab	Protein sequence	DNA sequence	Protein Activation in Podocytes			CpG Methylation in Podocytes		
					NG+ 5adc	HG	HG + 5adc	NG+ 5adc	HG	HG + 5adc
**Insulin signalling**	INSR (Phospho-Tyr1361)	Ab-1361	syEEHIPytHMNGGK	N/A	0.02	0.54	0.70	Unaltered	Unaltered	Unaltered
	GAB1 (Phospho-Tyr659)	Ab-659	VADERVDyVVVDQQK	AGCAGTGTAGCAGATGAGAGAGTGGATTATGTT GTTGTTGACCAACAGAAGACCTTG	0.21	0.30	0.38	Down	Down	Down
	IKK alpha/beta (Phospho-Ser180/181)	Ab-180/181	N/A	N/A	0.92	0.93	0.98	Unaltered	Unaltered	Unaltered
	GRB10 (Phospho-Tyr67)	Ab-67	N/A	N/A	0.50	0.53	0.34	Unaltered	Unaltered	Unaltered
	GRB2 (Phospho-Ser159)	Ab-159	N/A	N/A	0.14	0.17	0.38	Unaltered	Unaltered	Unaltered
	RAS	p21 H and K	N/A	N/A	-0.07	0.31	0.26	Unaltered	Unaltered	Unaltered
	MEK2 (Phospho-Thr394)	Ab-286	LRLNQPGtPtRtAV	Unknown	0.38	0.43	0.46	Unaltered	Unaltered	Unaltered
	ERK1 (p44/42) (MAPK3)	Ab-204	HDHtGFLtEyVAtRW	GAGCATGACCACACCGGCTTCCTGACGGAGTA TGTGGCTACGCGCTGGTAC	0.29	0.51	0.66	Down	Down	Down
	IRS-1/2	Ab-307	TRRsRtEsltAtsPA	Unknown	0.47	0.55	0.56	Down	Down	Down
	c-Raf (Phospho-Ser296)	Ab-296	N/A	N/A	0.23	0.41	0.73	Down	Down	Down
	PI3K p85-alpha (Phospho-Tyr607)	Ab-4	NENtEDQysLVEDDE	GGCAATGAAAACACTGAAGACCAATATTCACTG GTGGAAGATGATGAAGAT	0.30	0.30	0.32	Down	Down	Down
	BAD (Phospho-Ser91/128)	Ab-91/128	N/A	N/A	0.46	0.46	0.36	Unaltered	Unaltered	Unaltered
	GSK3 beta (Phospho-Ser9)	Ab-9	N/A	N/A	0.56	0.44	0.75	Unaltered	Unaltered	Unaltered
	PTEN (Phospho-Ser380/Thr382/Thr383)	Ab-380/382/383	N/A	N/A	-0.42	-0.66	-0.68	Unaltered	Unaltered	Unaltered
	SHP-2 (Phospho-Tyr580)	Ab-580	N/A	N/A	-0.65	-0.82	-0.30	Unaltered	Unaltered	Unaltered
	Shc (Phospho-Tyr349)	Ab-349	N/A	N/A	-0.39	-1.31	-1.06	Unaltered	Unaltered	Unaltered
	ATP-Citrate Lyase (Phospho-Ser454)	Ab-454	N/A	N/A	0.15	0.15	0.36	Unaltered	Unaltered	Unaltered
	PKC theta (Phospho-Ser676)	Ab-676	N/A	N/A	0.79	0.41	0.60	Unaltered	Unaltered	Unaltered
	FOXO1/3/4-PAN (Phospho-Thr24/32)	Ab-24/32	N/A	N/A	-0.07	-0.60	-0.07	Unaltered	Unaltered	Unaltered
	PKA (Phospho-Thr197)	Ab-197	N/A	N/A	0.58	0.41	0.60	Unaltered	Unaltered	Unaltered
	AMPK1/AMPK2 (Phospho-Ser485/491)	Ab-485/491	N/A	N/A	-0.35	-0.99	-0.55	UP	UP	UP
	HSL (Phospho-Ser554)	Ab-554	N/A	N/A	1.03	1.21	1.25	Unaltered	Unaltered	Unaltered
**mTOR signalling**	PDK1 (Phospho-Ser241)	Ab-241	skQARANsFVGtAQy	N/A	-0.08	0.11	-0.19	Unaltered	Unaltered	Unaltered
	AKT1 (Phospho-Ser124)	Ab-124	EMDFRsGsPsDNsGA	N/A	0.14	0.12	0.01	Unaltered	Unaltered	Unaltered
	mtTOR (Phospho-ser2448)	Ab-2448	RsRtRtDsysAGQsV	AGCGATCCCGAACGAGGACGGATTCCTACTCT GCTGGCCAGTCAGTCGAAATTTTGG	0.24	0.52	0.52	Down	Down	Down
	mTOR (Phospho-Ser2481)	Ab-2481	tVPEsIHsFIGDGLV	N/A	-0.06	0.12	-0.18	Unaltered	Unaltered	Unaltered
	mTOR (Phospho-Thr2246)	Ab-2246	NKRsRtRtDsysAGQ	N/A	-0.48	-0.71	-0.63	Unaltered	Unaltered	Unaltered
	p70S6K (Phospho-Ser411)	Ab-411	DDStLSEsANQVFLG	N/A	0.35	0.55	1.01	Unaltered	Unaltered	Unaltered
	p70S6k beta (Phospho-Ser423)	Ab-423	N/A	N/A	0.22	0.23	0.68	Unaltered	Unaltered	Unaltered
	4E-BP1 (Phospho-Ser65)	Ab-65	FLMECrNsPVtktPP	N/A	0.40	0.86	0.79	Unaltered	Unaltered	Unaltered
	eIF4E (Phospho-Ser209)	Ab-209	DtATksGsttKNRFV	N/A	0.74	0.79	0.98	Unaltered	Unaltered	Unaltered

Supplementary Table 12 DNA methylation and common SNP allele frequencies comparison in the FinnDiane replication cohort.

Gene; DMR location (hg19)	SNP ID* and position (hg19)		SNP creates or disrupts a potential methylation site (CpG)	Alleles (A1>A2)	A2 (minor) allele frequency						
					Whole cohort (n =265)	Diabetic nephropathy groups				*P**	*P***
						Normo-albuminuria *n*=62	Micro-albuminuria *n*=71	Macro-albuminuria *n*=65	ESRD *n*=69		
***TXNRD1;* DMR in chr12:104687047–104687538**											
	rs10735394	chr12:104,687,142	no	A>C	0.146	0.145	0.169	0.131	0.138	0.82	0.398
	rs10861186	chr12:104,687,212	yes	G>A	0.406	0.427	0.415	0.408	0.377	0.855	0.76
	rs143922410	chr12:104,687,360	no	C>T	0.013	0.024	0.014	0.008	0.007	0.627	0.398
***MTOR;* DMR in chr1:11,307,861–11,309,108**											
	rs56797473	chr1:11,308,509	no	G>A	0.049	0.033	0.063	0.023	0.072	0.184	0.697
	rs6670821	chr1:11,308,762	yes	C>T	0.256	0.303	0.211	0.231	0.283	0.275	0.354
***RPTOR;* DMR in chr17:78,549,199–78,549,667**											
	rs76584516	chr17:78,549,237	no	A>G	0.062	0.097	0.077	0.038	0.037	0.117	0.39
	rs4890047	chr17:78,549,439	no	C>T	0.344	0.344	0.317	0.331	0.384	0.672	0.564
	rs4890048	chr17:78,549,602	no	G>A	0.346	0.355	0.317	0.331	0.384	0.662	0.479
***LCAT;* DMR in chr16:67,975,360–67,975,798**											
	rs140576021	chr16:67,975,539	yes	C >T	0.024	0.025	0.022	0.026	0.023	1	0.775
***COL1A2;* DMR in chr7:94,025,127–94,026,207**											
	rs201691876	chr7:94,025,132	no	A>C	0.017	0.033	0.007	0	0.03	0.071	0.523
	rs151000015	chr7:94025217	no	T>C	0.027	0.025	0.022	0.039	0.022	0.85	0.302
	rs117782609	chr7:94025671	no	T>G	0.009	0.025	0.007	0	0.007	0.226	0.438
	rs79174778	chr7:94025737	no	A>G	0.015	0.016	0.014	0.015	0.014	1	0.49
	rs388625	chr7:94025924	yes	G>A	0.365	0.403	0.324	0.377	0.362	0.594	0.951

No SNP with minor allele frequency of ≥0.01 was available in our imputed SNP data for *SMPD3* gene differentially methylated region (DMR) at chr16: 68,399,935– 68,400,661 or *IRS2* gene DMR at chr13:110,443,346–110,443,787

*χ^2 test or Fisher's exact test *P* value comparing A_2 allele frequencies in the diabetic nephropathy groups

***P* value from a linear regression analysis of DNA methylation levels of the corresponding DMR and the SNP. SNP genotype was coded by the number of minor (A2) alleles (0,1,2, and additive model).

Supplementary Table 13 Primer sequences used for Methyl-qPCR and *In vitro* studies

Assay and Primer name	Sequence (hg19)	Amplicon size (bp)
DNA_MTOR_region_promoter_F	ACCTCAGTCACTTCCACTCA	231
DNA_MTOR_region_promoter_R	ATCCATAAAGAGCGCTAGCC	
DNA_MTOR_region_exon7_F	GCATACTGCTGTGACTACTGACCT	263
DNA_MTOR_region_exon7_R	GTATTTTTAATAGGGACGGGG	
DNA_MTOR_region_exon10_F	GGACCCATCACATCACAATTAC	195
DNA_MTOR_region_exon10_R	ACTGCCTTCTTGTCCCTGAT	
DNA_MTOR_region_exon38_F	TCAAGGAGTAAGTTCACAGC	260
DNA_MTOR_region_exon38_R	GGCTTGTTTCTCTGCTTCCA	
DNA_RPTOR_DMR_F	GTCTGTCAGCAGGGCAGTAG	176
DNA_RPTOR_DMR_R	TAAGGGACAGAGCAGCTGAAAT	
DNA_IRS2_DMR_F	TGTGTTGCTTCATGTTGCTATC	224
DNA_IRS2_DMR_R	CCCACTTCATAACATCTGTACTCT	
DNA_COL1A2_DMR_F	CACTGATGTCCACCTTGTAAAACT	277
DNA_COL1A2_DMR_R	GAAAGGCTCCAACCAGAAATG	
DNA_TXNRD1_DMR_F	GATATTGGCTCATTGCAACCTT	251
DNA_TXNRD1_DMR_R	GCTGCCTTTTCTCTTATCTCTTTG	
DNA_LCAT_DMR_F	AGATTGAGACTGCGGCTATGAT	206
DNA_LCAT_DMR_R	CACACAAGGAGCAGAAAGACAC	
DNA_SMPD3_DMR_F	CCAAACCTTAGCCTAACAGCAT	243
DNA_SMPD3_DMR_R	TTAGTCGAGATGGGGTTTCATC	
DNA_Controlregion_TRAPPC9_DMR_F	CCTGTGCAGTGCATCTCTGTAT	221
DNA_Controlregion_TRAPPC9_DMR_R	CAGAGGAGCAGAGCTGGTAGG	
mRNA_MTOR_F	TTGCTTGAGGTGCTACTG	183
mRNA_MTOR_R	CTGACTTGACTTGGATTCTG	
mRNA_MTOR_exon6_F	GCAAGTGCAACCCTTCTTTG	284
mRNA_MTOR_exon6_R	CATTTCTTCTCTCAGACGCTCTC	
mRNA_MTOR_exon7_F	GCAAGTGCAACCCTTCTTTG	268
mRNA_MTOR_exon7_R	ACTGGCACACCTGATCAAA	
mRNA_MTOR_exon8_F	AGAGCGTCTGAGAGAAGAAATG	552
mRNA_MTOR_exon8_R	ACTGGCACACCTGATCAAA	
mRNA_RPTOR_F	GATCGATCCAGCATTCCAAG	131
mRNA_RPTOR_R	TCAGAGCTGGAGGATGAAGG	
mRNA_IRS2_F	CGGTGAGTTCTACGGGTACAT	194
mRNA_IRS2_R	TCAGGGTGTATTCATCCAGCG	
mRNA_COL1A2_F	GCCCCCCAGGCAGAGA	118
mRNA_COL1A2_R	CCAACTCCTTTTCCATCATACTGA	
mRNA_TXNRD1_F	GCCCTGCAAGACTCTCGAAATTA	163
mRNA_TXNRD1_R	GCCCATAAGCATTCTCATAGACGA	
mRNA_H3F3A_F	GGTGTCTTCAAAAAGGCCAA	109
mRNA_H3F3A_R	GCGAGAAATTGCTCAGGACT	
Bisulfite_MTOR_region_exon7_F	AACAATAAAGCAGCAGCAGAGGG	396
Bisulfite_MTOR_region_exon7_R	CTGTAGTCCTGGCTACTTGGG	

*DNA primers were used for Methyl-qPCR and ChIP-qPCR validation experiments

References

1. Gross JL, de Azevedo MJ, Silveiro SP, Canani LH, Caramori ML, and Zelmanovitz T. Diabetic nephropathy: diagnosis, prevention, and treatment. *Diabetes Care.* 2005;28(1):164-76.
2. Thorn LM, Forsblom C, Fagerudd J, Thomas MC, Pettersson-Fernholm K, Saraheimo M, et al. *Diabetes Care*. 2005:2019-24.
3. Levey AS, Stevens LA, Schmid CH, Zhang YL, Castro AF, 3rd, Feldman HI, et al. A new equation to estimate glomerular filtration rate. *Ann Intern Med.* 2009;150(9):604-12.
4. Jones SR, Carley S, and Harrison M. An introduction to power and sample size estimation. *Emerg Med J.* 2003;20(5):453-8.
5. Li CI, Samuels DC, Zhao YY, Shyr Y, and Guo Y. Power and sample size calculations for high-throughput sequencing-based experiments. *Brief Bioinform.* 2018;19(6):1247-55.
6. Tsai PC, and Bell JT. Power and sample size estimation for epigenome-wide association scans to detect differential DNA methylation. *Int J Epidemiol.* 2015;44(4):1429-41.
7. Zuo C, and Keles S. A statistical framework for power calculations in ChIP-seq experiments. *Bioinformatics.* 2014;30(6):753-60.
8. Syreeni A, Sandholm N, Cao J, Toppila I, Maahs DM, Rewers MJ, et al. Genetic Determinants of Glycated Hemoglobin in Type 1 Diabetes. *Diabetes.* 2019;68(4):858-67.
9. Tam CH, Ma RC, So WY, Wang Y, Lam VK, Germer S, et al. Interaction effect of genetic polymorphisms in glucokinase (GCK) and glucokinase regulatory protein (GCKR) on metabolic traits in healthy Chinese adults and adolescents. *Diabetes.* 2009;58(3):765-9.
10. Ziemann M, Harikrishnan K, Khurana I, Kaspi A, Keating ST, De Silva DA, et al. Clinical applicability of methylome sequencing in a pilot study of ischemic stroke individuals. *Methods in Next Generation Sequencing.* 2015;2(1).
11. Feng J, Liu T, and Zhang Y. Using MACS to identify peaks from ChIP-Seq data. *Curr Protoc Bioinformatics.* 2011;Chapter 2:Unit 2 14.
12. Zhang Y, Liu T, Meyer CA, Eeckhoute J, Johnson DS, Bernstein BE, et al. Model-based analysis of ChIP-Seq (MACS). *Genome Biol.* 2008;9(9):R137.
13. Quinlan AR. BEDTools: The Swiss-Army Tool for Genome Feature Analysis. *Curr Protoc Bioinformatics.* 2014;47:11 2 1-34.
14. Quinlan AR, and Hall IM. BEDTools: a flexible suite of utilities for comparing genomic features. *Bioinformatics.* 2010;26(6):841-2.

15. Harrow J, Frankish A, Gonzalez JM, Tapanari E, Diekhans M, Kokocinski F, et al. GENCODE: the reference human genome annotation for The ENCODE Project. *Genome Res.* 2012;22(9):1760-74.
16. Robinson MD, McCarthy DJ, and Smyth GK. edgeR: a Bioconductor package for differential expression analysis of digital gene expression data. *Bioinformatics.* 2010;26(1):139-40.
17. Myers RH, Montgomery DC, Vining GG, and Robinson TJ. *Generalized Linear Models*. John Wiley & Sons, Inc.; 2012:202-71.
18. Houseman EA, Accomando WP, Koestler DC, Christensen BC, Marsit CJ, Nelson HH, et al. DNA methylation arrays as surrogate measures of cell mixture distribution. *BMC Bioinformatics*. 2012;13:86.
19. Gu Z, Eils R, and Schlesner M. Complex heatmaps reveal patterns and correlations in multidimensional genomic data. *Bioinformatics.* 2016;32(18):2847-9.
20. Halachev K, Bast H, Albrecht F, Lengauer T, and Bock C. EpiExplorer: live exploration and global analysis of large epigenomic datasets. *Genome Biol.* 2012;13(10):R96.
21. Consortium EP. The ENCODE (ENCyclopedia Of DNA Elements) Project. *Science.* 2004;306(5696):636-40.
22. Lister R, Pelizzola M, Dowen RH, Hawkins RD, Hon G, Tonti-Filippini J, et al. Human DNA methylomes at base resolution show widespread epigenomic differences. *Nature.* 2009;462(7271):315-22.
23. Barrera V, and Peinado MA. Evaluation of single CpG sites as proxies of CpG island methylation states at the genome scale. *Nucleic Acids Res.* 2012;40(22):11490-8.
24. Croft D, Mundo AF, Haw R, Milacic M, Weiser J, Wu G, et al. The Reactome pathway knowledgebase. *Nucleic Acids Res.* 2014;42(Database issue):D472-7.
25. Fabregat A, Sidiropoulos K, Garapati P, Gillespie M, Hausmann K, Haw R, et al. The Reactome pathway Knowledgebase. *Nucleic Acids Res.* 2016;44(D1):D481-7.
26. Subramanian A, Tamayo P, Mootha VK, Mukherjee S, Ebert BL, Gillette MA, et al. Gene set enrichment analysis: a knowledge-based approach for interpreting genome-wide expression profiles. *Proc Natl Acad Sci U S A.* 2005;102(43):15545-50.
27. Merico D, Isserlin R, Stueker O, Emili A, and Bader GD. Enrichment map: a network-based method for gene-set enrichment visualization and interpretation. *PLoS One.* 2010;5(11):e13984.

28. Shannon P, Markiel A, Ozier O, Baliga NS, Wang JT, Ramage D, et al. Cytoscape: a software environment for integrated models of biomolecular interaction networks. *Genome Res.* 2003;13(11):2498-504.
29. Okabe J, Orlowski C, Balcerczyk A, Tikellis C, Thomas MC, Cooper ME, et al. Distinguishing hyperglycemic changes by Set7 in vascular endothelial cells. *Circ Res.* 2012;110(8):1067-76.
30. Dobin A, Davis CA, Schlesinger F, Drenkow J, Zaleski C, Jha S, et al. STAR: ultrafast universal RNA-seq aligner. *Bioinformatics.* 2013;29(1):15-21.
31. Li LC, and Dahiya R. MethPrimer: designing primers for methylation PCRs. *Bioinformatics.* 2002;18(11):1427-31.
32. Kumaki Y, Oda M, and Okano M. QUMA: quantification tool for methylation analysis. *Nucleic Acids Res.* 2008;36(Web Server issue):W170-5.
33. Levey AS, Eckardt KU, Tsukamoto Y, Levin A, Coresh J, Rossert J, et al. Definition and classification of chronic kidney disease: a position statement from Kidney Disease: Improving Global Outcomes (KDIGO). *Kidney Int.* 2005;67(6):2089-100.
34. National Kidney F. KDOQI Clinical Practice Guideline for Diabetes and CKD: 2012 Update. *Am J Kidney Dis.* 2012;60(5):850-86.
35. Chen Z, Miao F, Paterson AD, Lachin JM, Zhang L, Schones DE, et al. Epigenomic profiling reveals an association between persistence of DNA methylation and metabolic memory in the DCCT/EDIC type 1 diabetes cohort. *Proc Natl Acad Sci U S A.* 2016;113(21):E3002-11.
36. De Marinis Y, Cai M, Bompada P, Atac D, Kotova O, Johansson ME, et al. Epigenetic regulation of the thioredoxin-interacting protein (TXNIP) gene by hyperglycemia in kidney. *Kidney Int.* 2016;89(2):342-53.
37. Houshmand-Oeregaard A, Hjort L, Kelstrup L, Hansen NS, Broholm C, Gillberg L, et al. DNA methylation and gene expression of TXNIP in adult offspring of women with diabetes in pregnancy. *PLoS One.* 2017;12(10):e0187038.
38. Parikh H, Carlsson E, Chutkow WA, Johansson LE, Storgaard H, Poulsen P, et al. TXNIP regulates peripheral glucose metabolism in humans. *PLoS Med.* 2007;4(5):e158.
39. Shah A, Xia L, Masson EA, Gui C, Momen A, Shikatani EA, et al. Thioredoxin-Interacting Protein Deficiency Protects against Diabetic Nephropathy. *J Am Soc Nephrol.* 2015;26(12):2963-77.
40. Soriano-Tarraga C, Jimenez-Conde J, Giralt-Steinhauer E, Mola-Caminal M, Vivanco-Hidalgo RM, Ois A, et al. Epigenome-wide association study identifies TXNIP gene

associated with type 2 diabetes mellitus and sustained hyperglycemia. *Hum Mol Genet.* 2016;25(3):609-19.

41. Shah UJ, Xie W, Flyvbjerg A, Nolan JJ, Hojlund K, Walker M, et al. Differential methylation of the type 2 diabetes susceptibility locus KCNQ1 is associated with insulin sensitivity and is predicted by CpG site specific genetic variation. *Diabetes Res Clin Pract.* 2019;148:189-99.
